# Supplementary material for: Community engagement for birth preparedness and complication readiness in the Community Level Interventions for Pre-eclampsia (CLIP) Trial in India: a mixed-method evaluation
Source: BMJ Open. 2022 Dec 20;12(12):e060593. doi: 10.1136/bmjopen-2021-060593 (PMC9772674; doi:10.1136/bmjopen-2021-060593)

BMJ Publishing Group Limited (BMJ) disclaims all liability and responsibility arising from any reliance placed on this supplemental material which has been supplied by the author(s)

SUPPLEMENTARY FILES

**Table S1:** Consort 2020 Checklist	Page 2
**Table S2:** Standards for Reporting Qualitative Research (SRQR) Checklist	Page 6
**Table S3:** Summary of pre-eclampsia knowledge by cluster [n (%)]	Page 9
**Table S4:** Summary of birth preparedness by cluster [n (%)]	Page 11
**Figure S1:** Informational poster on danger signs of hypertensive disorders of pregnancy	Page 12

Kavi A, *et al. BMJ Open* 2022; 12:e060593. doi: 10.1136/bmjopen-2021-060593

Table S1 Consort 2020 Checklist

CONSORT 2010 checklist of information to include when reporting a randomised trial*

Section/Topic	Item No	Checklist item	Reported on page No
**Title and abstract**			
	1a	Identification as a randomised trial in the title	Page 1
	1b	Structured summary of trial design, methods, results, and conclusions (for specific guidance see CONSORT for abstracts)	Page 2
**Introduction**			
Background and objectives	2a	Scientific background and explanation of rationale	Page 4-5
	2b	Specific objectives or hypotheses	Page 5
**Methods**			
Trial design	3a	Description of trial design (such as parallel, factorial) including allocation ratio	Page 5
	3b	Important changes to methods after trial commencement (such as eligibility criteria), with reasons	N/A*
Participants	4a	Eligibility criteria for participants	Page 5-6
	4b	Settings and locations where the data were collected	Page 5
Interventions	5	The interventions for each group with sufficient details to allow replication, including how and when they were actually administered	Page 6

Kavi A, et al. BMJ Open 2022; 12:e060593. doi: 10.1136/bmjopen-2021-060593

Outcomes	6a	Completely defined pre-specified primary and secondary outcome measures, including how and when they were assessed	Page 7
	6b	Any changes to trial outcomes after the trial commenced, with reasons	N/A*
Sample size	7a	How sample size was determined	N/A*
	7b	When applicable, explanation of any interim analyses and stopping guidelines	N/A*
Randomisation:			
Sequence generation	8a	Method used to generate the random allocation sequence	Page 5
	8b	Type of randomisation; details of any restriction (such as blocking and block size)	Page 5
Allocation concealment mechanism	9	Mechanism used to implement the random allocation sequence (such as sequentially numbered containers), describing any steps taken to conceal the sequence until interventions were assigned	Page 5
Implementation	10	Who generated the random allocation sequence, who enrolled participants, and who assigned participants to interventions	Page 5
Blinding	11a	If done, who was blinded after assignment to interventions (for example, participants, care providers, those assessing outcomes) and how	N/A*
	11b	If relevant, description of the similarity of interventions	N/A*
Statistical methods	12a	Statistical methods used to compare groups for primary and secondary outcomes	Page 7-8
	12b	Methods for additional analyses, such as subgroup analyses and adjusted analyses	
**Results**			
Participant flow (a diagram is	13a	For each group, the numbers of participants who were randomly assigned, received intended treatment, and were analysed for the primary outcome	Page 5*

strongly recommended)	13b	For each group, losses and exclusions after randomisation, together with reasons	N/A*
Recruitment	14a	Dates defining the periods of recruitment and follow-up	Page 6
	14b	Why the trial ended or was stopped	Page 6
Baseline data	15	A table showing baseline demographic and clinical characteristics for each group	Page 5*
Numbers analysed	16	For each group, number of participants (denominator) included in each analysis and whether the analysis was by original assigned groups	N/A*
Outcomes and estimation	17a	For each primary and secondary outcome, results for each group, and the estimated effect size and its precision (such as 95% confidence interval)	Page 9-11
	17b	For binary outcomes, presentation of both absolute and relative effect sizes is recommended	Page 5*
Ancillary analyses	18	Results of any other analyses performed, including subgroup analyses and adjusted analyses, distinguishing pre-specified from exploratory	N/A*
Harms	19	All important harms or unintended effects in each group (for specific guidance see CONSORT for harms)	N/A*
**Discussion**			
Limitations	20	Trial limitations, addressing sources of potential bias, imprecision, and, if relevant, multiplicity of analyses	Page 14
Generalisability	21	Generalisability (external validity, applicability) of the trial findings	Page 13-14
Interpretation	22	Interpretation consistent with results, balancing benefits and harms, and considering other relevant evidence	Page 13-14

Kavi A, *et al. BMJ Open* 2022; 12:e060593. doi: 10.1136/bmjopen-2021-060593

**Other information**			
Registration	23	Registration number and name of trial registry	Page 5
Protocol	24	Where the full trial protocol can be accessed, if available	N/A*
Funding	25	Sources of funding and other support (such as supply of drugs), role of funders	Page 15

*For more details on the methodology, implementation and results of the full CLIP Trial in India, please see: Bellad MB, Goudar SS, Mallapur AA, Sharma S, Bone J, Charantimath US, et al. Community level interventions for pre-eclampsia (CLIP) in India: A cluster randomised controlled trial. Pregnancy Hypertension. 2020 Jul 1;21:166–75.

Kavi A, *et al. BMJ Open* 2022; 12:e060593. doi: 10.1136/bmjopen-2021-060593

Table S2: Standards for Reporting Qualitative Research (SRQR) Checklist

Title and abstract	Page/line no(s).
**Title** - Concise description of the nature and topic of the study Identifying the study as qualitative or indicating the approach (e.g., ethnography, grounded theory) or data collection methods (e.g., interview, focus group) is recommended	Page 1
**Abstract** - Summary of key elements of the study using the abstract format of the intended publication; typically includes background, purpose, methods, results, and conclusions	Page 2

Introduction

**Problem formulation** - Description and significance of the problem/phenomenon studied; review of relevant theory and empirical work; problem statement	Page 4-5
**Purpose or research questio**n - Purpose of the study and specific objectives or questions	Page 5

Methods

**Qualitative approach and research paradigm** - Qualitative approach (e.g., ethnography, grounded theory, case study, phenomenology, narrative research) and guiding theory if appropriate; identifying the research paradigm (e.g., postpositivist, constructivist/ interpretivist) is also recommended; rationale**	Page 8
**Researcher characteristics and reflexivity** - Researchers' characteristics that may influence the research, including personal attributes, qualifications/experience, relationship with participants, assumptions, and/or presuppositions; potential or actual interaction between researchers' characteristics and the research questions, approach, methods, results, and/or transferability	Page 8

**Context** - Setting/site and salient contextual factors; rationale**	Page 5-6
**Sampling strategy** - How and why research participants, documents, or events were selected; criteria for deciding when no further sampling was necessary (e.g., sampling saturation); rationale**	Page 6-7
**Ethical issues pertaining to human subjects** - Documentation of approval by an appropriate ethics review board and participant consent, or explanation for lack thereof; other confidentiality and data security issues	Page 8
**Data collection methods** - Types of data collected; details of data collection procedures including (as appropriate) start and stop dates of data collection and analysis, iterative process, triangulation of sources/methods, and modification of procedures in response to evolving study findings; rationale**	Page 8
**Data collection instruments and technologies** - Description of instruments (e.g., interview guides, questionnaires) and devices (e.g., audio recorders) used for data collection; if/how the instrument(s) changed over the course of the study	Page 8
**Units of study** - Number and relevant characteristics of participants, documents, or events included in the study; level of participation (could be reported in results)	Page 6
**Data processing** - Methods for processing data prior to and during analysis, including transcription, data entry, data management and security, verification of data integrity, data coding, and anonymization/de-identification of excerpts	Page 8
**Data analysis** - Process by which inferences, themes, etc., were identified and developed, including the researchers involved in data analysis; usually references a specific paradigm or approach; rationale**	Page 8
**Techniques to enhance trustworthiness** - Techniques to enhance trustworthiness and credibility of data analysis (e.g., member checking, audit trail, triangulation); rationale**	Page 8

Results/findings

Kavi A, *et al. BMJ Open* 2022; 12:e060593. doi: 10.1136/bmjopen-2021-060593

**Synthesis and interpretation** - Main findings (e.g., interpretations, inferences, and themes); might include development of a theory or model, or integration with prior research or theory	Page 11-13
**Links to empirical data** - Evidence (e.g., quotes, field notes, text excerpts, photographs) to substantiate analytic findings	Page 11-13

Discussion

**Integration with prior work, implications, transferability, and contribution(s) to the field -** Short summary of main findings; explanation of how findings and conclusions connect to, support, elaborate on, or challenge conclusions of earlier scholarship; discussion of scope of application/generalizability; identification of unique contribution(s) to scholarship in a discipline or field	Page 13-14
**Limitations** - Trustworthiness and limitations of findings	Page 14

Other

**Conflicts of interest** - Potential sources of influence or perceived influence on study conduct and conclusions; how these were managed	Page 15
**Funding** - Sources of funding and other support; role of funders in data collection, interpretation, and reporting	Page 15

Table S3:Summary of pre-eclampsia knowledge by cluster [n (%)]

	Intervention Clusters						Control Clusters						Total
	**Hosur**	**Kanagala**	**Sangolli**	**Pattadaka**	**Amingad**	**Sutugundar**	**Aihole**	**Galagali**	**Katageri**	**Belavadi**	**Chachadi**	**Ammangi**	
Composite	122 (7.5)	17 (1.6)	62 (5.9)	4 (0.3)	221 (13.2)	13 (1.3)	6 (0.6)	94 (6.9)	12 (1.1)	3 (0.2)	26 (2.0)	10 (1.0)	590 (3.4)
Can name at least 4 symptoms	122 (7.0)	17 (1.6)	62 (5.9)	4 (0.3)	223 (13.3)	13 (1.3)	6 (0.6)	96 (7.0)	12 (1.1)	3 (0.2)	26 (2.0)	10 (1.0)	594 (4.0)
Aware women can have abnormal bleeding in pregnancy	656 (40.3)	325 (30.5)	406 (38.8)	296 (21.3)	171 (10.2)	127 (12.3)	36 (3.9)	1034 (75.4)	268 (24.1)	494 (39.0)	442 (34.2)	94 (9.5)	4349 (29.4)
Aware women can have seizure in pregnancy	624 (38.3)	316 (29.7)	345 (32.9)	276 (19.8)	145 (8.6)	108 (10.5)	19 (2.1)	810 (59.1)	260 (23.4)	334 (26.4)	236 (18.3)	32 (3.2)	3505 (23.7)
Aware women can have high blood pressure in pregnancy	817 (50.1)	584 (54.8)	553 (52.8)	264 (19.0)	732 (43.6)	193 (18.8)	38 (4.1)	1000 (72.9)	339 (30.5)	718 (56.7)	512 (39.7)	466 (47.2)	6216 (42.1)

Aware high blood pressure in pregnancy can be life threatening	793 (48.7)	584 (54.8)	552 (52.7)	232 (16.7)	736 (43.8)	189 (18.4)	37 (4.0)	968 (70.6)	232 (20.9)	626 (49.5)	511 (39.6)	466 (47.2)	5926 (40.1)

Kavi A, *et al. BMJ Open* 2022; 12:e060593. doi: 10.1136/bmjopen-2021-060593

Table S4: Summary of birth preparedness by cluster [n (%)]

	Intervention Clusters						Control Clusters						Total
	**Hosur**	**Kanagala**	**Sangolli**	**Pattadakallu**	**Amingad**	**Sutugundar**	**Aihole**	**Galagali**	**Katageri**	**Belavadi**	**Chachadi**	**Ammangi**	
Composite	1556 (95.5)	1013 (95.1)	1044 (99.7)	869 (62.5)	466 (27.8)	639 (62.1)	702 (76.5)	878 (64.0)	827 (74.4)	1241 (98.0)	1289 (99.9)	932 (94.4)	11456 (77.5)
Arranged transport	1548 (95.0)	995 (93.4)	1044 (99.7)	1160 (83.5)	475 (28.3)	887 (86.2%)	720 (78.4)	1065 (77.7)	859 (77.3)	1262 (99.7)	1291 (100.0)	848 (85.9)	12154 (82.2)
Has permission for emergency care	1626 (99.8)	1063 (99.8)	1045 (99.8)	1372 (98.7)	1634 (97.3)	855 (83.0)	909 (99.0)	1362 (99.3)	1015 (91.4)	1264 (99.8)	1291 (100.0)	984 (99.7)	14420 (97.5)
Has money saved for emergency	1324 (81.2)	1008 (94.6)	907 (86.6)	127 (9.1)	385 (22.9)	118 (11.4)	481 (52.4)	327 (23.9)	364 (32.8)	723 (57.1)	1168 (90.5)	977 (99.0)	7909 (53.5)
Identified facility for delivery	1572 (96.5)	1058 (99.3)	1002 (95.7)	1011 (72.7)	1377 (82.0)	836 (81.2)	745 (81.2)	997 (72.7)	874 (78.7)	1210 (95.6)	1266 (98.1)	903 (91.5)	12851 (86.9)

Kavi A, *et al. BMJ Open* 2022; 12:e060593. doi: 10.1136/bmjopen-2021-060593

Figure S1: Informational poster on danger signs of hypertensive disorders of pregnancy

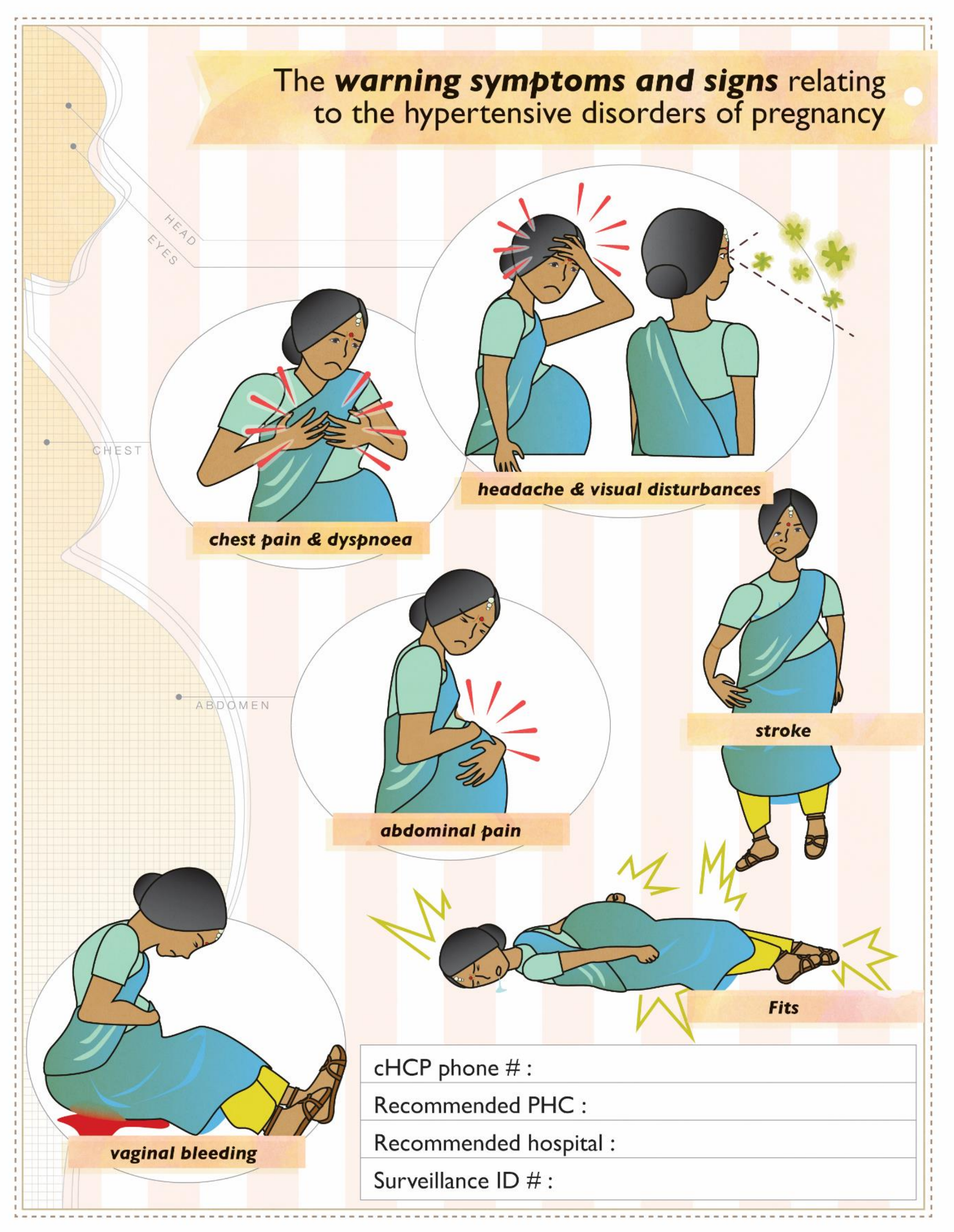

Supplement: Supplementary data [file bmjopen-2021-060593supp001.pdf]
