# Supplementary material for: Community engagement for birth preparedness and complication readiness in the Community Level Interventions for Pre-eclampsia (CLIP) Trial in India: a mixed-method evaluation
Source: BMJ Open. 2022 Dec 20;12(12):e060593. doi: 10.1136/bmjopen-2021-060593 (PMC9772674; doi:10.1136/bmjopen-2021-060593)
Supplement: Author's manuscript [file bmjopen-2021-060593.draft_revisions.pdf]

BMJ Open is committed to open peer review. As part of this commitment we make the peer review history of every article we publish publicly available.

When an article is published we post the peer reviewers' comments and the authors' responses online. We also post the versions of the paper that were used during peer review. These are the versions that the peer review comments apply to.

The versions of the paper that follow are the versions that were submitted during the peer review process. They are not the versions of record or the final published versions. They should not be cited or distributed as the published version of this manuscript.

BMJ Open is an open access journal and the full, final, typeset and author-corrected version of record of the manuscript is available on our site with no access controls, subscription charges or pay-per-view fees (http://bmjopen.bmj.com).

If you have any questions on BMJ Open's open peer review process please email
info.bmjopen@bmj.com

BMJ Open

Evaluating the effect of community engagement on the birth preparedness and complication readiness in the Community Level Interventions for Pre-eclampsia (CLIP) randomised trial in northern Karnataka, India

Journal:	*BMJ Open*
Manuscript ID	bmjopen-2021-060593
Article Type:	Original research
Date Submitted by the Author:	06-Jan-2022
Complete List of Authors:	Kavi, Avinash; KLE Academy of Higher Education and Research<br>Kinshella, Mai-Lei Woo; The University of British Columbia Faculty of Medicine, Department of Obstetrics and Gynaecology<br>Ramadurg, Umesh Y.; S Nijalingappa Medical College, Women's and Children's Health Research Unit<br>Charantimath, Umesh; KLE Academy of Higher Education and Research, Women's and Children's Health Research Unit<br>Katageri, Geetanjali M.; S Nijalingappa Medical College, Women's and Children's Health Research Unit<br>Karadiguddi, Chandrashekhar C.; KLE Academy of Higher Education and Research<br>Honnungar, Narayan V.; KLE Academy of Higher Education and Research<br>Bannale, Shashidhar G.; S Nijalingappa Medical College, Women's and Children's Health Research Unit<br>Mungarwadi, Geetanjali I.; KLE Academy of Higher Education and Research<br>Bone, Jeffrey; The University of British Columbia, Obstetrics and Gynecology<br>Vidler, Marianne; The University of British Columbia, Obstetrics & Gynaecology<br>Magee, Laura; King's College London School of Medical Education,<br>Mallapur, Ashalata; BVVS S Nijalingappa Medical College and HSK Hospital and Research Centre<br>Goudar, Shivaprasad S.; KLE University, Women's and Children's Health Research Unit<br>Bellad, Mrutyunjaya; KLE Academy of Higher Education and Research, Women's and Children's Health Research Unit<br>Derman, Richard ; Thomas Jefferson University<br>von Dadelszen, Peter ; King's College London Faculty of Life Sciences and Medicine, Department of Women and Children's Health<br>The CLIP India Working Group, .; The University of British Columbia Faculty of Medicine, Department of Obstetrics and Gynaecology
Keywords:	OBSTETRICS, Public health < INFECTIOUS DISEASES, Hypertension < CARDIOLOGY

For peer review only - http://bmjopen.bmj.com/site/about/guidelines.xhtml

I, the Submitting Author has the right to grant and does grant on behalf of all authors of the Work (as defined in the below author licence), an exclusive licence and/or a non-exclusive licence for contributions from authors who are: i) UK Crown employees; ii) where BMJ has agreed a CC-BY licence shall apply, and/or iii) in accordance with the terms applicable for US Federal Government officers or employees acting as part of their official duties; on a worldwide, perpetual, irrevocable, royalty-free basis to BMJ Publishing Group Ltd ("BMJ") its licensees and where the relevant Journal is co-owned by BMJ to the co-owners of the Journal, to publish the Work in this journal and any other BMJ products and to exploit all rights, as set out in our licence.

The Submitting Author accepts and understands that any supply made under these terms is made by BMJ to the Submitting Author unless you are acting as an employee on behalf of your employer or a postgraduate student of an affiliated institution which is paying any applicable article publishing charge ("APC") for Open Access articles. Where the Submitting Author wishes to make the Work available on an Open Access basis (and intends to pay the relevant APC), the terms of reuse of such Open Access shall be governed by a Creative Commons licence – details of these licences and which Creative Commons licence will apply to this Work are set out in our licence referred to above.

Other than as permitted in any relevant BMJ Author's Self Archiving Policies, I confirm this Work has not been accepted for publication elsewhere, is not being considered for publication elsewhere and does not duplicate material already published. I confirm all authors consent to publication of this Work and authorise the granting of this licence.

For peer review only

Evaluating the effect of community engagement on the birth preparedness and complication readiness in the Community Level Interventions for Pre-eclampsia (CLIP) randomised trial in northern Karnataka, India

Avinash Kavi[1*]
Mai-Lei Woo Kinshella[2]
Umesh Y. Ramadurg[3]
Umesh S. Charantimath[1]
Geetanjali M. Katageri[3]
Chandrashekhar C. Karadiguddi[1]
Narayan V. Honnungar[1]
Shashidhar G. Bannale[3]
Geetanjali I. Mungarwadi[1]
Jeffrey N. Bone[2]
Marianne Vidler[2]
Laura A. Magee[4]
Ashalata A. Mallapur[3]
Shivaprasad S. Goudar[1]
Mrutyunjaya Bellad[1]
Richard J. Derman[5]
Peter von Dadelszen[4]
and the CLIP India Working Group

1. Women's and Children's Health Research Unit, KLE Academy of Higher Education and Research's Jawaharlal Nehru Medical College, Belagavi, Karnataka, India
2. Department of Obstetrics and Gynaecology and BC Children's Hospital Research Institute, University of British Columbia, Vancouver BC, Canada
3. Women's and Children's Health Research Unit, S. Nijalingappa Medical College, Bagalkote, Karnataka, India
4. Department of Women and Children's Health, School of Life Course Sciences, Kings College London, London, UK
5. Thomas Jefferson University, Philadelphia, USA

*Corresponding author:
Avinash Kavi

Women's and Children's Health Research Unit, KLE Academy of Higher Education and Research's Jawaharlal Nehru Medical College, Belagavi, Karnataka, India

Email: dravinashkavi@gmail.com

Word count: 3432/4000

For peer review only - http://bmjopen.bmj.com/site/about/guidelines.xhtml

For peer review only

ABSTRACT

Objective: To describe the process of community engagement in northern Karnataka, India and its impact on pre-eclampsia knowledge, birth preparedness and complication readiness, pregnancy-related care seeking and maternal morbidity.

Design: Community engagement was conducted in intervention clusters within the Indian "Community-Level Interventions for Pre-eclampsia" Trial. Community engagement attendance was summarized according to participant group. Pre-eclampsia knowledge, birth preparedness, health services engagement and perinatal outcomes was evaluated within trial surveillance. Outcomes were compared between trial arms using a mixed effects logistic regression model on RStudio (RStudio Inc, Boston, United States). Community feedback notes were thematically analysed on NVivo12 (QSR International, Melbourne, Australia).

Setting: Belagavi and Bagalkote districts in rural Karnataka, India.

Participants: Pregnant women and women of reproductive age, mothers and mothers-in-law, community stakeholders and male household decision-makers, and health workers.

Results: A total of 1,379 community engagement meetings were conducted with 39,362 participants between November 2014 and October 2016. Community engagement activities may have had an effect on modifying community attitudes towards hypertension in pregnancy and its complications. However, rates of pre-eclampsia knowledge, birth preparedness, health services engagement and maternal morbidities among individual pregnant women were not significantly impacted by community engagement activities in their area.

Conclusion: Evaluation of our community engagement program in India demonstrates the feasibility of reaching pregnant women alongside household decision-makers, community stakeholders and health workers. More research is needed to explore the pathways of impact between broad community mobilization to strengthen support for maternal care-seeking and clinical outcomes of individual pregnant women.

Trial registration: NCT01911494

Keywords: Community Engagement, Birth preparedness, Complication readiness, Pre-eclampsia, Community support, Barriers.

For peer review only - http://bmjopen.bmj.com/site/about/guidelines.xhtml

STRENGTHS AND LIMITATIONS OF THIS STUDY

- We systematically evaluated the effectiveness of a large-scale community engagement program conducted with the Community Level Interventions for Pre-eclampsia (CLIP) Trial in rural India using a process evaluation framework. The logic model outlines the hypothesized relationships between inputs, outputs and outcomes of interest to explore potential pathways of change.
- Data for the assessment was obtained from a prospective population-based surveillance system of women in the CLIP Trial and community engagement records including community feedback notes.
- The community engagement program and its evaluation involved a wide range of health workers, pregnant women, household decision-makers and community leaders.
- The limited timeframe within a clinical trial may have been too short to appropriately evaluate community-level behavioural change and more research is needed to better understand the influence of external factors on maternal health care-seeking.
- Community engagement was one of the components of a complex intervention evaluated in the CLIP Trial and interventions involving multiple factors makes the impact assessment more complicated.

For peer review only

For peer review only - http://bmjopen.bmj.com/site/about/guidelines.xhtml

INTRODUCTION

Over 94% of maternal deaths occur in low- and middle-income countries (LMICs), primarily in South Asia and sub-Saharan Africa, and are preventable (1,2). Maternal deaths relate primarily to delays in triage (ability of care provider and women to identify who is severely ill and requires urgent care), transport (ability to get women to appropriate care when needed) and treatment (ability to provide appropriate treatment when care accessed) (3). Increased birth preparedness and complication readiness can help mitigate against delays and ensure timely identification of the need for seeking skilled care and arrival at the appropriate facility for pregnancy complications. Birth preparedness includes knowledge of pregnancy danger signs, identification of health facility and transport for birth and in case of complications, identification of a birth companion and preferred birth attendant, supplies to bring to the facility, funds for any expenses, and identification of a support person to look after other children at home as well as compatible blood donors if needed (4).

By identifying women at highest risk of complications at the community-level, transportation and treatment can be targeted to those most in need in a timely manner. Involvement of community members and raising community awareness of health in pregnancy are critical in the implementation of community health programs to reduce delays and improve maternal health services (5). Engaging the community is vital for effective functioning of the health system; however, involving communities in the implementation of the programs can be challenging.

Over the past few decades, community engagement (CE) has emerged as an increasingly effective strategy for harnessing community potential, particularly for health improvement (6–11). CE is defined as 'a process of working collaboratively with groups of people who are affiliated by geographic proximity, special interests, or similar situations, with respect to issues affecting their well-being' (12). It is a dynamic relational process that facilitates communication, interaction, involvement, and exchange between an organization and a community for a range of social and organizational outcomes (13). This strategy has been used for health promotion, research, and policy making to address a variety of health issues (14–17). CE enables a more contextualized understanding of the perceptions and contexts of the members of the community, thereby facilitating stronger relationships.

This paper intends to describe the process of CE and its impact on knowledge around pre-eclampsia, birth preparedness and complication readiness, pregnancy-related care seeking and rates of maternal morbidity.

METHODS

The CLIP India Trial was one of three independently-powered cluster randomised controlled trials (cRCT); the others took place in Pakistan and Mozambique (NCT01911494). We undertook a two-phased community (including primary health

For peer review only - http://bmjopen.bmj.com/site/about/guidelines.xhtml

centre (PHC)-level) cRCT in rural Karnataka in Belagavi and Bagalkote districts (**Figure 1 map**) where the incidence of pregnancy hypertension is 10.3% (18). Belagavi and Bagalkote include 1278, and 627 villages, respectively. The adult female literacy rate varies in the two districts, at 70·2% in Belagavi, and 59·3% in Bagalkote, respectively (19). More detail on the CLIP India Trial has been published elsewhere (20). In brief, the unit of randomization was the PHC area and restricted, stratified randomisation was undertaken to allocate 12 clusters to the intervention and control groups, using population size as a single stratification factor. The CLIP Trial focused on implementing community-level evidence-based care to reduce all cause maternal and perinatal mortality and major morbidity by supporting early identification and prompt referrals for pregnancy complications. The intervention included engagement of the community and its stakeholders; blood pressure assessment by Accredited Social Health Activists (ASHA); and usage of PIERS-On-the-Move (POM). POM is a mobile-based application decision aid for community health workers to identify women at risk of adverse outcomes from pregnancy hypertension and guide management, including treatment with oral antihypertensive medication, intramuscular magnesium sulphate, and referral to the hospital depending on the calculated risk (21,22).

The CE component was carried out in intervention clusters over the entire trial period (November 2014 - October 2016). The CE activities aimed at creating culturally and contextually appropriate discussion to improve maternal health awareness and action around the prevention of maternal morbidity and mortality. CE aimed to improve birth preparedness, nutrition, appropriate antenatal care (ANC) and promote appropriate health care seeking. Topics included warning signs of pregnancy complications, permission to seek care, identifying a skilled birth attendant, facility for delivery, and mode of transport as well as saving up funds for transport and treatment. Discussions also included adverse pregnancy outcomes and success stories in the community, overcoming local barriers to accessing maternal health care and the interventions in the CLIP Trial.

Process of facilitating community engagement

Community engagement meetings were primarily held at the anganwadi (AW) centres in the village, which offer basic health services. These were locations where pregnant women and children routinely access ANC, immunizations and other maternal and child health care services. Meetings were also held at sub-centres, PHCs, temples, public halls, schools or other appropriate nearby locations for large monthly sessions.

The activities convened multiple levels of the local health care system. This included anganwadi workers (AWW) at the AW centres, ASHAs who worked in the villages and focused on connecting mothers and children to primary care, Auxiliary Nurse Midwives (ANMs) who worked at sub-centres, Lady Health Visitors (LHV) who supervised the ANMs, and medical officers who coordinated the PHC. In addition, ASHA supervisors and Registry Administrators (RAs) appointed as a part of the trial attended meetings.

For peer review only - http://bmjopen.bmj.com/site/about/guidelines.xhtml

Feasibility studies in these communities revealed that pregnant women rarely made health care decisions on their own, and other family members and community leaders were highly influential in this decision making (23). Consequently, CE activities involved household decision-makers who were often in charge of women's decisions to seek care, along with community leaders, such as elected representatives and teachers, who provided advice and at times provided financial assistance.

The CE activity schedule was prepared at the beginning of each month by the local study team and shared to the respective community stakeholders. Meetings lasted between 30 and 150 minutes. These meetings were facilitated by the study team members involving the local health officials led by medical officers of PHCs. Sessions began with a short formal introduction and description of the purpose, participants were encouraged to actively participate, share personal experiences and discuss case examples. Reviewing local experiences was considered the best way to share information with participants; therefore, recent cases of pregnancy complications, referrals and outcomes were discussed. There were no monetary incentives for participants for attending the meeting. Participants were provided with nutritional snacks and fruits.

Data collection and analysis

A prospective population-based surveillance system was established as part of the CLIP India Trial, modelled on the National Institute of Health Global Network's Maternal and Newborn Health (MNH) Registry (24). Married women of reproductive age likely to conceive within 12 months were identified by an annual household survey, they were consented and enrolled into the CLIP Trial once pregnant. Data were collected soon after enrolment, soon after delivery and at 42 days postpartum from the mother in the local language by cluster PHC health care workers identified as registry administrators (RAs). Data collection included demographic factors, birth preparedness, care-seeking, obstetric information, pre-eclampsia knowledge, perinatal outcomes and treatment information at different time points as specified above. Data forms were reviewed for completeness and entered weekly into the local database. De-identified and encrypted data were transferred to the central server for analyses. For CE activities, study staff and supervisors documented the number of participants and community feedback after each meeting.

Figure 2 illustrates the strategy for assessing CE in the CLIP India Trial, based on the process evaluation framework for the CLIP Pilot Trial in Nigeria (25). CE attendance was summarized according to participant group: pregnant women and other women of reproductive age, the pregnant women's mothers and mothers-in-law, community stakeholders (community leaders, male and female decision-makers), health workers (AWW, ANM, ASHA, ASHA supervisor, LHV, medical officers, nurses, RA) and others (family members, neighbours, friends, community members).

Pre-eclampsia knowledge evaluated awareness of: 1) abnormal bleeding in pregnancy, 2) high blood pressure during pregnancy, 3) seizures in pregnancy, 4) that pregnancy

For peer review only - http://bmjopen.bmj.com/site/about/guidelines.xhtml

hypertension can be life threatening; by recalling at least one of the above conditions and at least four symptoms of high blood pressure in pregnancy for the composite pre-eclampsia knowledge score. Birth preparedness and complication readiness (BPCR) evaluated a pregnant woman's plans for: 1) transport in case of emergencies, 2) permission to seek emergency care, 3) money saved for an emergency, 4) identified health facility for delivery, and 5) at least two of the components for the BCPR composite score.

Pre-eclampsia knowledge, birth preparedness, health services engagement and perinatal outcomes indicators were summarized between arms with counts and frequencies for categorical variables and medians and interquartile range [IQR] for continuous variables. For health service questions, we restricted the denominators to women who delivered by trial end as these indicators are more likely to occur later in pregnancy. As pre-eclampsia knowledge and birth preparedness were asked throughout pregnancy and all women received community engagement, we included all women in assessing these rates. Outcomes were further compared between trial arms using a mixed effects logistic regression model (to account for clustering) with a logit link. Models were adjusted for age, maternal education, husband education, gestational age at booking, and parity and results are presented as odds ratios and 95% confidence intervals. Quantitative data were analysed using RStudio (RStudio Inc, Boston, United States), which was complemented by qualitative community feedback recorded by study staff. The community feedback notes were imported to NVivo12 software (QSR International, Melbourne, Australia) for thematic analysis.

Research ethics approval

The project received ethics committee approvals from KLE Academy of Higher Education and Research Deemed-to-be-University (MDC/IECHSR/2011-12/A-4; ICMR 5/7/859/12-RHN) and the University of British Columbia (UBC, H12-03497).

Patient and public involvement

Neither patients nor public were involved in the design or conduct of the study.

RESULTS

A total of 1,379 CE meetings were conducted. Among them 586 sessions were conducted in Belagavi district and 773 sessions at Bagalkote district. The median number of sessions per cluster was 204 [IQR 186 – 281]. **Table 1** describes the activities across clusters and the participants reached. As long as pregnant women remained in the same village, they were encouraged to attend all sessions. The other participants, such as stakeholders and decision-makers, were usually different for each session. Pregnant women were most represented at the meetings, followed by mothers-in-law who often accompanied them. Husbands and fathers-in-law were least

For peer review only - http://bmjopen.bmj.com/site/about/guidelines.xhtml

For peer review only

represented in the meetings as they often had competing priorities; however, overall sessions were well attended by target groups.

Table 1: Participants reached in CE activities

Target groups	Number of participants (% of total)	Number of sessions (% of total)
Pregnant women and other women of reproductive age	22,149 (56.3%)	1,379 (100.0%)
Mothers and mothers-in-law	4,643 (11.8%)	1,140 (82.7%)
Community stakeholders	5,685 (14.4%)	1,147 (83.2%)
Health workers	4,752 (12.1%)	1,376 (99.8%)
Others	2,113 (5.4%)	N/A
Total	39,362	1,379

Staff noted that they appreciated engaging household decision-makers and community stakeholders alongside pregnant women and health workers. Household decision-makers may not have been aware of pre-eclampsia and other maternal health risks and their inclusion in the discussion supported care for pregnant women and increased value in attending ANC.

> "The people appreciated these community engagement meetings. They said that they learnt many things about the care of the pregnant women, which they were not aware of earlier. The pregnant women became more aware about their health and their parents also started giving more support and care to her."

> "Earlier, it was difficult for the woman to take permission from the decision makers in the family to seek antenatal care. But after conducting the community engagement meetings, the views of the decision-makers were changed so that the seeking of permission for antenatal care became easy."

Tables 2 and **3** present summaries of pre-eclampsia knowledge and birth preparedness, the rates for both (composite and components) were not significantly different between arms. Only a minority of participants from both intervention and control (2-6%) could name at least four symptoms of high blood pressure in pregnancy or had overall pre-eclampsia knowledge. In contrast, there were higher levels of birth preparedness among women in both intervention and control with a majority of women reporting that they had arranged transport, has permission for emergency care, and identified a health facility for delivery. Only saving funds for an obstetric emergency was lower, with about half of women declaring that they had funds saved up. Confidence intervals of

For peer review only - http://bmjopen.bmj.com/site/about/guidelines.xhtml

odd ratios are wide reflecting high heterogeneity between study clusters [see **Tables S1 and S2**].

Table 2: Summary of pre-eclampsia knowledge by arm

Reported awareness that:	Intervention	Control	Total	Adjusted odds ratio (95%CI); p-value*
Women can have abnormal bleeding in pregnancy	1,981 (25.3%)	2,368 (34.1%)	4,349 (29.4%)	0.88 (0.25, 3.18); 0.85
Women can have seizure in pregnancy	1,814 (23.1%)	1,691 (24.4%)	3,505 (23.7%)	1.49 (0.40, 5.62); 0.55
Women can have high blood pressure in pregnancy	3,143 (40.1%)	3,073 (44.3%)	6,216 (42.1%)	1.02 (0.32, 3.25); 0.97
High blood pressure in pregnancy can be life threatening	3,086 (39.4%)	2,840 (40.9%)	5,926 (40.1%)	0.86 (0.17, 4.21); 0.85
Can name at least four symptoms	441 (5.6%)	153 (2.2%)	594 (4.0%)	2.35 (0.60, 9.12); 0.22
Composite**	439 (5.6%)	151 (2.2%)	590 (4.0%)	2.35 (0.61, 9.10); 0.22

**Estimated by mixed effects logistic regression adjusted for: maternal age, maternal education, husband education, gestational age at enrolment, parity, and cluster (random effect).*

*** Recall at least one condition and at least four symptoms of high blood pressure*

Table 3: Summary of birth preparedness by arm

	Intervention	Control	Total	Adjusted odds ratio (95%CI); p-value*
Arranged transport	6,109 (77.9%)	6,045 (87.1%)	12,154 (82.2%)	0.40 (0.03, 6.07); 0.51
Has permission for emergency care	7,595 (96.9%)	6,825 (98.3%)	14,420 (97.5%)	0.48 (0.05, 4.31); 0.51
Has money saved for emergency	3,869 (49.4%)	4,040 (58.2%)	7,909 (53.5%)	0.46 (0.05, 4.19); 0.49
Identified facility for delivery	6,856 (87.5%)	5,995 (86.3%)	12,851 (86.9%)	1.50 (0.36, 6.27); 0.58
Composite**	5,587 (71.3%)	5,869 (84.5%)	11,456 (77.5%)	0.50 (0.05, 5.59); 0.58

**Estimated by mixed effects logistic regression adjusted for: maternal age, maternal education, husband education, gestational age at enrolment, parity, and cluster (random effect).*

*** Having at least two of the components of birth preparedness ready.*

For peer review only - http://bmjopen.bmj.com/site/about/guidelines.xhtml

For peer review only

CE feedback indicates an increased awareness of antenatal care as an important component of birth preparedness. Staff noted that through CE activities, decision-makers and community leaders gave more value to preparation for delivery and pregnancy complications.

> "The women became more aware of the antenatal and postnatal care. The decision-makers and the community leaders started giving more support in antenatal and postnatal care of the woman."

> "Most of the participants found the discussion about the pregnant woman's care to be unique, as they were not used to prepare in advance, in anticipation of complications."

While staff noted that there were changing opinions on the value of ANC and birth preparedness in the community, **Table 4** illustrates that health services engagement by pregnant women in the CLIP Trial were similar between arms. Almost all women had at least one ANC visit and three out of four women had at least four visits. Approximately one in ten women visited the PHC for reason other than routine care and about 3% of women were admitted to a health facility for reasons other than delivery, staying a median of three days. One in twenty women experienced a maternal morbidity and there was no significant difference between arms (OR 0.95, 95%CI: 0.61-1.50, p= 0.84).

Table 4: Health services engagement and maternal morbidity by arm

	**Intervention** (N=6,908)	**Control** (N=6,109)	**Total** (N=13,017)	**Adjusted odds ratio (95%CI); p-value***
Women with at least one ANC visit	6,891 (99.8%)	6,068 (99.3%)	12,959 (99.6%)	Cannot be computed
Women with 4 or more ANC visits	5,140 (74.4%)	4,745 (77.7%)	9,885 (75.9%)	0.86 (0.51, 1.44); 0.57
Visited PHC for other than routine care	823 (11.9%)	739 (12.1%)	1562 (12.0%)	1.92 (0.43, 8.55); 0.39
Visited higher level facility for other than routine care	1,069 (15.5%)	780 (12.8%)	1849 (14.2%)	1.59 (0.58, 4.38); 0.37
Number of women admitted to health facility for reasons other than delivery	225 (3.3%)	170 (2.8%)	395 (3.0%)	1.28 (0.08, 19.3); 0.86
Median days of admission [IQR]	3.0 [2.0; 5.0]	3.0 [2.0; 5.0]	3.0 [2.0; 5.0]	-
Maternal morbidity **	371/7,839 (4.7%)	325/6,944 (4.7%)	696/14,783 (4.7%)	0.95 (0.61, 1.50); 0.84

**Estimated by mixed effects logistic regression adjusted for: maternal age, maternal education, husband education, gestational age at enrolment, parity, and cluster (random effect).*

For peer review only - http://bmjopen.bmj.com/site/about/guidelines.xhtml

***Data taken from CLIP India primary trial paper and includes different adjustment factors* (20)

Community support for maternal health

CE activities supported mobilizing community leaders to better support maternal health locally. There were examples of community leaders advocating for appropriate maternal health care seeking with household decision-makers as well as pushing for change to support local health systems.

> "The views of stakeholders also changed so much that the arrangement of transport, financial support, counselling the decision-makers became effective. For example, in the initial period of the study, there was one incident where the husband, the male decision-maker of the family, of the woman with eclampsia refused to take her to the hospital but after counselling by the community leaders and his family doctor, he agreed to take her to the higher care hospital."

> "The community leaders recognized the significant role played by the ASHAs and the PHC personnel in promoting the health of the pregnant women. The PHC did not have an ambulance of its own and the community leaders saw to it that a new ambulance was made available at the PHC. They also built a room above the PHC building as a meeting place for the AHSAs to continue the work actively."

External factors and barriers

Barriers of poverty, health infrastructure gaps and poor quality of care at facilities were frequently highlighted in CE discussions.

> "People accepted all the topics. They only found the issue of arranging money to be a problem because they had meagre earnings."

Additionally, the cost of treatment at private hospitals was often brought up in discussions in conjunction with the lack of services at government facilities. Some participants pointed out that there was no obstetric specialist at a nearby secondary level hospital for a period of time. Staff reflected that community members sometimes highlighted the need to strengthen care at facilities in addition to raising community awareness.

> "There were some suggestions from the community about the improvement of services at the local government health centres."

At referral facilities, participants reported that they often had to wait a long time before they would be provided services. They also reported that rural, uneducated people may encounter stigma and discrimination when seeking care at urban tertiary facilities.

For peer review only - http://bmjopen.bmj.com/site/about/guidelines.xhtml

For peer review only

"The participants wanted to know how to get immediate admission and care at higher and bigger tertiary care hospitals."

"They also demanded about early admission and prompt service at the tertiary care hospitals as there are some uneducated persons in the community who face some problems in getting the services in bigger hospitals."

DISCUSSION

Our evaluation of the CLIP CE program in India demonstrates the feasibility of reaching pregnant women alongside household decision-makers, community stakeholders and health workers. This initiative involved almost 40,000 participants in 1,379 sessions over a period of two years. Bringing different parts of local communities together to discuss pregnancy care may have helped to shift local opinions on the value of ANC to monitor for pregnancy complications and mitigate risks. However, rates of pre-eclampsia knowledge, birth preparedness, health services engagement and maternal morbidities remained similar across arms in the CLIP Trial. According to our evaluation framework (**Figure 2**), our study found no evidence that the outputs were associated with our expected outcomes.

Although research findings related to the effect of community participation to improve access to maternal health care services have been mixed (26), a previous study in Uttar Pradesh in Northern India found significant effects of community health worker home visits and monthly community meetings on improved self-recognition of problems faced during pregnancy, birth preparedness components and knowledge of pregnancy danger signs (27). Differing outcomes between our findings and the study in Uttar Pradesh highlight two issues: first, the importance of context, and secondly, challenges in measuring impacts of community mobilization. Uttar Pradesh has much higher maternal mortality rates than Karnataka (28), suggesting that CE may have a stronger effect where there is lower baseline access to maternal health services. Secondly, in contrast to targeted community meetings with traditional maternal-care providers and birth attendants, our CE strategy was focused on broader community mobilization and strengthening support for maternal care-seeking among community stakeholders, which has previously been discussed in the literature as challenging to measure (5,29).

CE involved shifting opinions on maternal health and cultures of care, behavioural change and mobilization and can take more time than the limited timeframe within a clinical trial. Logic models can help to understand the pathways of change, however, some of our expected short-term effects in birth preparedness via community mobilization may take more time than measured during the trial. A systematic review found increased knowledge of birth preparedness and complication readiness as a result of interventions but increased knowledge did not always correspond to increased utilization of maternal health services (30). While the CE program was successful in reaching a wide audience of participants and community feedback suggested increased

For peer review only - http://bmjopen.bmj.com/site/about/guidelines.xhtml

support for maternal health care-seeking, these may be upstream indicators or insufficient to change clinical outcomes.

A systematic review on the effects of community participation on improving uptake of skilled maternal and newborn care highlighted the importance of qualitative research to understand unforeseen impacts and socio-political factors (26). While quantitative results did not reveal an effect in our study, staff qualitatively noted that effects were noticeable in the opinions of community leaders and household decision-makers in their support for pregnancy care. Effects of CE on community leaders and household decision-makers were not adequately captured in our evaluation framework that focused on measuring effects with only the pregnant women enrolled in the trial. Additionally, the discrepancy between the quantitative and qualitative results may also highlight the influence of external factors where community members gained appreciation of maternal health issues but were limited in their capacity to access care. Poverty and quality of care available at local health facilities emerged as significant barriers shared in community feedback, which is in line with previous research on the determinants of maternal health care services utilization in the area (23).

Nevertheless, qualitative findings that suggested improved awareness and attitudes towards pregnancy hypertension and its complications among community leaders and household decision-makers is promising. Previous research in the area found lack of knowledge around pre-eclampsia and eclampsia (31). Hypertension specifically as a pregnancy condition was unknown and there was no specific terminology in the local language (Kannada) for pre-eclampsia.

CE has significant repercussions for the implementation of health care interventions and policy making. In addition to the individual-level factors, community-level factors play a vital role in understanding whether or not a woman is able to accept maternal health interventions. However, since CE was one of the components of a complex intervention, the effects assessed do not reflect CE alone. Intervention of multiple factors makes the impact assessment much more complicated.

CONCLUSION

Our study found that CE activities may have had an effect on modifying community attitudes towards hypertension in pregnancy and its complications but did not find an impact on individual women's pre-eclampsia knowledge, birth preparedness and care-seeking indicators. Contemplation of specific individual factors and community capabilities in engaging in the health seeking behaviours would contribute to the vision of supporting people-centred methodologies to deliver health promotion. Through the complex social interactions inherent in the current CE activities, appraisals over time will evaluate the effectiveness and sustainability of these interventions.

For peer review only - http://bmjopen.bmj.com/site/about/guidelines.xhtml

ACKNOWLEDGEMENTS

We would like to thank the women and their families who contributed data to this trial, in addition to the ASHA and ANMs who deliver care to the most vulnerable populations. Also, we would like to acknowledge the key contributions of the following DSMB members: Romano Nkumbwa Byaruhanga, Brian Darlow, Eileen Hutton (Chair), and Lehana Thabane.

We would also like to acknowledge the CLIP India Working Group members: Shashidhar G Bannale, Keval S Chougala, Vaibhav B Dhamanekar, Anjali M Joshi, Namdev A Kamble, Gudadayya S Kengapur, Uday S Kudachi, Sphoorthi S Mastiholi, Geetanjali I Mungarwadi, Esperança Sevene, Khátia Munguambe, Charfudin Sacoor, Eusébio Macete, Helena Boene, Felizarda Amose, Orvalho Augusto, Cassimo Bique, Ana Ilda Biz, Rogério Chiaú, Silvestre Cutana, Paulo Filimone, Emília Gonçálves, Marta Macamo, Salésio Macuacua, Sónia Maculuve, Ernesto Mandlate, Analisa Matavele, Sibone Mocumbi, Dulce Mulungo, Zefanias Nhamirre, Ariel Nhancolo, Cláudio Nkumbula, Vivalde Nobela, Rosa Pires, Corsino Tchavana, Anifa Vala, Faustino Vilanculo, Rahat N Qureshi, Sana Sheikh, Zahra Hoodbhoy, Imran Ahmed, Amjad Hussain, Javed Memon, Farrukh Raza, Olalekan O Adetoro, John O Sotunsa, Sharla K Drebit, Chirag Kariya, Mansun Lui, Diane Sawchuck, Ugochi V Ukah, Mai-Lei Woo Kinshella, Shafik Dharamsi, Guy A Dumont, Tabassum Firoz, Ana Pilar Betrán, Susheela M Engelbrecht, Veronique Filippi, William A Grobman, Marian Knight, Ana Langer, Simon A Lewin, Gwyneth Lewis, Craig Mitton, Nadine Schuurman, James G Thornton, France Donnay, Romano N Byaruhanga, Brian Darlow, Eileen Hutton, Mario Merialdi, Lehana Thabane, Kelly Pickerill.

For peer review

DECLARATIONS OF INTEREST

The authors declare that they have no competing interests.

ROLE OF THE FUNDING SOURCE

This trial was funded by the University of British Columbia, a grantee of the Bill & Melinda Gates Foundation (PRE-EMPT initiative, grant number OPP1017337). Following input into trial design, the Gates Foundation had no role in data collection, analysis, or interpretation, or writing of the report.

AUTHOR CONTRIBUTIONS

PVD, RJD, MBB, SSG, AAM, LAM conceptualised the trial and components of the intervention. MBB, SSG, AAM, GIM, SGB, NVH, CCK, GMK, USC, UYR, AV, MV implemented and co-ordinated monitoring of the trial. JB and MWK performed the quantitative and qualitative analyses. AK and MWK wrote the first draft of the manuscript. All authors provided feedback and review of the manuscript.

For peer review only - http://bmjopen.bmj.com/site/about/guidelines.xhtml

DATA AVAILABILITY

The datasets used and/or analysed during the current study are available from the corresponding author on reasonable request.

FIGURE CAPTIONS

Figure 1. Belagavi and Bagalkote Districts, Karnataka State, India

Figure 2. A process evaluation plan for assessing community engagement to improve maternal health in Karnataka, India

For peer review only

REFERENCES

1. WHO, UNICEF, UNFPA, World Bank Group, The United Nations Population Division. Trends in maternal mortality 2000 to 2017. Geneva: World Health Organization; 2019.

2. Goldenberg RL, McClure EM, Saleem S. Improving pregnancy outcomes in low- and middle-income countries. Reprod Health. 2018 Jun 22;15(Suppl 1).

3. Thaddeus S, Maine D. Too far to walk: maternal mortality in context. Soc Sci Med. 1994;38(8):1091–110.

4. JHPIEGO. Monitoring Birth Preparedness and Complications Readiness: Tools and Indicators for Maternal and Newborn Health. Baltimore; 2004.

5. Cyril S, Smith BJ, Possamai-Inesedy A, Renzaho AMN. Exploring the role of community engagement in improving the health of disadvantaged populations: A systematic review. Glob Health Action. 2015;8.

6. Lassi ZS, Kumar R, Bhutta ZA. Community-Based Care to Improve Maternal, Newborn, and Child Health. In: Disease Control Priorities, Third Edition (Volume 2): Reproductive, Maternal, Newborn, and Child Health. Washington DC: The World Bank; 2016. p. 263–84.

7. Ricca J, Kureshy N, Leban K, Prosnitz D, Ryan L. Community-based intervention packages facilitated by NGOs demonstrate plausible evidence for child mortality impact. Health Policy Plan. 2014 Mar;29(2):204–16.

8. Rockers PC, Fink G, Zanolini A, Banda B, Biemba G, Sullivan C, et al. Impact of a community-based package of interventions on child development in Zambia: A cluster-randomised controlled trial. BMJ Glob Heal. 2016 Nov 1;1(3):104.

9. Prost A, Colbourn T, Seward N, Azad K, Coomarasamy A, Copas A, et al. Women's groups practising participatory learning and action to improve maternal and newborn health in low-resource settings: a systematic review and meta-analysis. Lancet. 2013 May 18;381(9879):1736–46.

10. Sadruddin S, Khan I ul H, Bari A, Khan A, Ahmad I, Qazi SA. Effect of community mobilization on appropriate care seeking for pneumonia in Haripur, Pakistan. J Glob Health. 2015 Jun;5(1):010405.

11. Mochache V, Irungu E, El-Busaidy H, Temmerman M, Gichangi P. "Our voices matter": A before-after assessment of the effect of a community-participatory intervention to promote uptake of maternal and child health services in Kwale, Kenya 11 Medical and Health Sciences 1117 Public Health and Health Services. BMC Health Serv Res. 2018 Dec 4;18(1).

12. Butterfoss FD, Francisco VT. Evaluating community partnerships and coalitions with practitioners in mind. Health Promot Pract. 2004 Apr 30;5(2):108–14.

For peer review only - http://bmjopen.bmj.com/site/about/guidelines.xhtml

13. NHS Department of Health and Human Services. Principles of Community Engagement [Internet]. Second edi. NIH Publication No. 11-7782. Bethesda, Maryland: National Institutes of Health (NIH); 2011 [cited 2020 Dec 15]. Available from: http://www.atsdr.cdc.gov/communityengagement/pdf/PCE_Report_508_FINAL.pdf

14. Johnston KA. Toward a Theory of Social Engagement. In: Johnston KA, Taylor M, editors. The Handbook of Communication Engagement. Hoboken, NJ, USA: John Wiley & Sons, Inc.; 2018. p. 17–32.

15. National Institute for Health and Care Excellence (NICE). Community engagement: improving health and wellbeing and reducing health inequalities . London: NICE; 2016.

16. Balcázar HG, de Heer H, Rosenthal L, Aguirre M, Flores L, Puentes FA, et al. A Promotores de Salud intervention to reduce cardiovascular disease risk in a high-risk hispanic border population, 2005-2008. Prev Chronic Dis. 2010;7(2).

17. Wells KB, Jones L, Chung B, Dixon EL, Tang L, Gilmore J, et al. Community-partnered cluster-randomized comparative effectiveness trial of community engagement and planning or resources for services to address depression disparities. J Gen Intern Med. 2013;28(10):1268–78.

18. Magee LA, Sharma S, Nathan HL, Adetoro OO, Bellad MB, Goudar S, et al. The incidence of pregnancy hypertension in India, Pakistan, Mozambique, and Nigeria: A prospective population-level analysis. PLOS Med. 2019;16(4):e1002783.

19. International Institute for Population Sciences (IIPS). National Family Health Survey (NFHS-4), 2015-16: India. Mumbai; 2017.

20. Bellad MB, Goudar SS, Mallapur AA, Sharma S, Bone J, Charantimath US, et al. Community level interventions for pre-eclampsia (CLIP) in India: A cluster randomised controlled trial. Pregnancy Hypertens. 2020 Jul 1;21:166–75.

21. Lim J, Cloete G, Dunsmuir DT, Payne BA, Scheffer C, von Dadelszen P, et al. Usability and Feasibility of PIERS on the Move: An mHealth App for Pre-Eclampsia Triage. JMIR mHealth uHealth. 2015 Apr 17;3(2):e37.

22. Payne B, Dunsmuir D, Hall D, Lim J, Sikandar R, Magee L, et al. Designing an evidence-based mobile health decision aid for the management of hypertensive disorders of pregnancy (HDP) in low-resourced settings. BMC Health Serv Res. 2014 Jul 7;14(S2):P93.

23. Vidler M, Ramadurg U, Charantimath U, Katageri G, Karadiguddi C, Sawchuck D, et al. Utilization of maternal health care services and their determinants in Karnataka State, India. Reprod Health. 2016 Jun 8;13(1):37.

For peer review only - http://bmjopen.bmj.com/site/about/guidelines.xhtml

24. Goudar SS, Carlo WA, McClure EM, Pasha O, Patel A, Esamai F, et al. The maternal and newborn health registry study of the global network for women's and children's health research. Int J Gynecol Obstet. 2012;118(3):190–3.

25. Sharma S, Adetoro OO, Vidler M, Drebit S, Payne BA, Akeju DO, et al. A process evaluation plan for assessing a complex community-based maternal health intervention in Ogun State, Nigeria. BMC Health Serv Res. 2017 Mar 28;17(1).

26. Marston C, Renedo A, McGowan CR, Portela A. Effects of Community Participation on Improving Uptake of Skilled Care for Maternal and Newborn Health: A Systematic Review. Braitstein P, editor. PLoS One. 2013 Feb 4;8(2):e55012.

27. Kumar V, Kumar A, Das V, Srivastava NM, Baqui AH, Santosham M, et al. Community-driven impact of a newborn-focused behavioral intervention on maternal health in Shivgarh, India. Int J Gynecol Obstet. 2012;117(1):48–55.

28. Dandona L, Dandona R, Kumar GA, Shukla DK, Paul VK, Balakrishnan K, et al. Nations within a nation: variations in epidemiological transition across the states of India, 1990–2016 in the Global Burden of Disease Study. Lancet. 2017 Dec 2;390(10111):2437–60.

29. MacQueen KM, Bhan A, Frohlich J, Holzer J, Sugarman J. Evaluating community engagement in global health research: The need for metrics Ethics in Biomedical Research. BMC Med Ethics. 2015 Jul 1;16(1):44.

30. Solnes Miltenburg A, Roggeveen Y, Shields L, van Elteren M, van Roosmalen J, Stekelenburg J, et al. Impact of Birth Preparedness and Complication Readiness Interventions on Birth with a Skilled Attendant: A Systematic Review. Niederkrotenthaler T, editor. PLoS One. 2015 Nov 23;10(11):e0143382.

31. Vidler M, Charantimath U, Katageri G, Ramadurg U, Karadiguddi C, Sawchuck D, et al. Community perceptions of pre-eclampsia in rural Karnataka State, India: A qualitative study. Reprod Health. 2016 Jun 8;13(1):35.

For peer review only - http://bmjopen.bmj.com/site/about/guidelines.xhtml

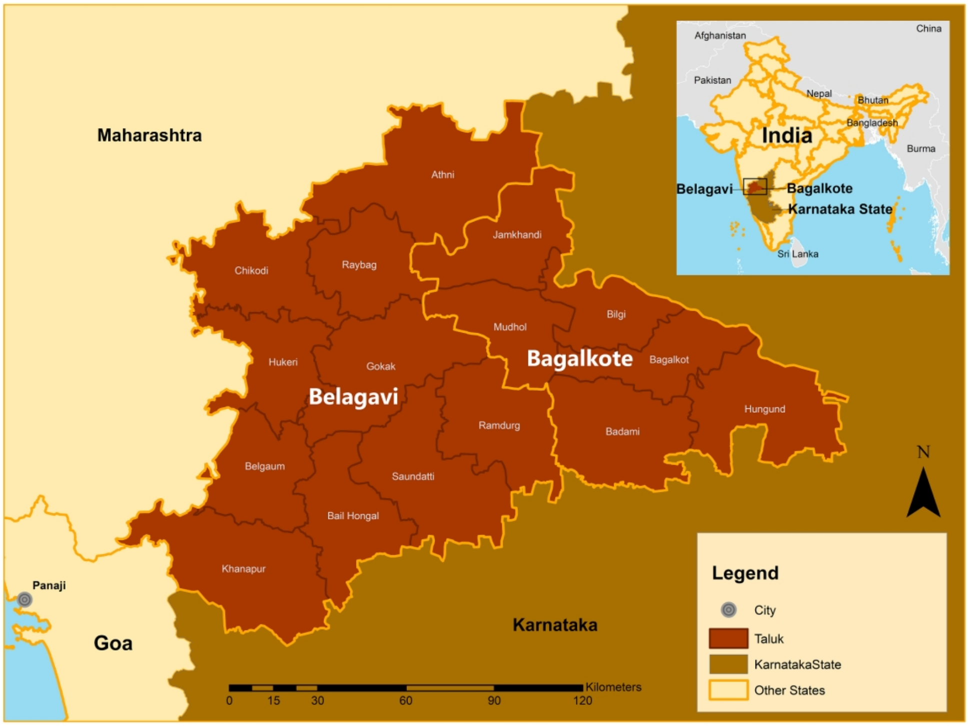
Maharashtra
Athni
Jamkhandi
Chikodi
Raybag
Mudhol
Bilgi
Hukeri
Gokak
Bagalkote
Bagalkot
Belagavi
Hungund
Ramdurg
Badami
Belgaum
Saundatti
Bail Hongal
Khanapur
Panaji
Goa
Karnataka
Kilometers
0 15 30 60 90 120
N
Legend
City
Taluk
KarnatakaState
Other States
Afghanistan
China
Pakistan
Nepal
Bhutan
India
Bangladesh
Burma
Belagavi
Bagalkote
Karnataka State
Sri Lanka

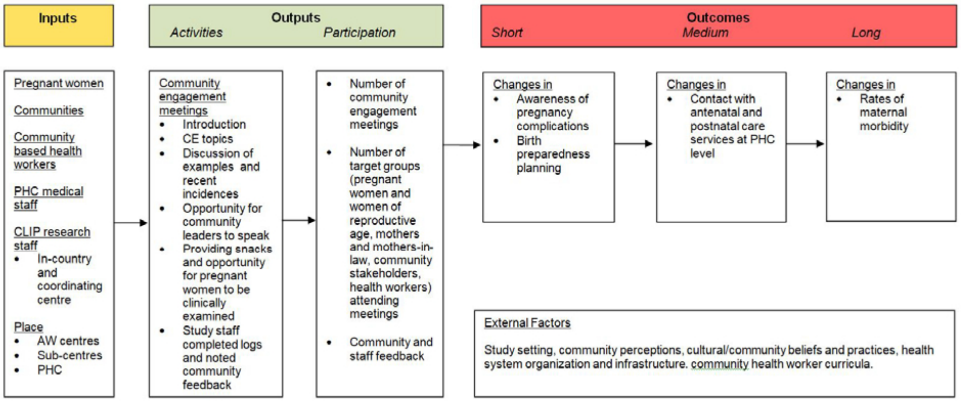
Inputs
Outputs
Activities
Participation
Outcomes
Short
Medium
Long
Pregnant women
Communities
Community based health workers
PHC medical staff
CLIP research staff
In-country and coordinating centre
Place
AW centres
Sub-centres
PHC
Community engagement meetings
Introduction
CE topics
Discussion of examples and recent incidences
Opportunity for community leaders to speak
Providing snacks and opportunity for pregnant women to be clinically examined
Study staff completed logs and noted community feedback
Number of community engagement meetings
Number of target groups (pregnant women and women of reproductive age, mothers and mothers-in-law, community stakeholders, health workers) attending meetings
Community and staff feedback
Changes in
Awareness of pregnancy complications
Birth preparedness planning
Changes in
Contact with antenatal and postnatal care services at PHC level
Changes in
Rates of maternal morbidity
External Factors
Study setting, community perceptions, cultural/community beliefs and practices, health system organization and infrastructure. community health worker curricula.

SUPPLEMENTARY FILES

Table S1: Summary of pre-eclampsia knowledge by cluster [n (%)]

	Intervention Clusters						Control Clusters						Total
	**Hosur**	**Kanagala**	**Sangolli**	**Pattadaka**	**Amingad**	**Sutugundar**	**Aihole**	**Galagali**	**Katageri**	**Belavadi**	**Chachadi**	**Ammangi**	
Composite	122 (7.5)	17 (1.6)	62 (5.9)	4 (0.3)	221 (13.2)	13 (1.3)	6 (0.6)	94 (6.9)	12 (1.1)	3 (0.2)	26 (2.0)	10 (1.0)	590 (3.4)
Can name at least 4 symptoms	122 (7.0)	17 (1.6)	62 (5.9)	4 (0.3)	223 (13.3)	13 (1.3)	6 (0.6)	96 (7.0)	12 (1.1)	3 (0.2)	26 (2.0)	10 (1.0)	594 (4.0)
Aware women can have abnormal bleeding in pregnancy	656 (40.3)	325 (30.5)	406 (38.8)	296 (21.3)	171 (10.2)	127 (12.3)	36 (3.9)	1034 (75.4)	268 (24.1)	494 (39.0)	442 (34.2)	94 (9.5)	4349 (29.4)
Aware women can have seizure in pregnancy	624 (38.3)	316 (29.7)	345 (32.9)	276 (19.8)	145 (8.6)	108 (10.5)	19 (2.1)	810 (59.1)	260 (23.4)	334 (26.4)	236 (18.3)	32 (3.2)	3505 (23.7)
Aware women can have high blood pressure in pregnancy	817 (50.1)	584 (54.8)	553 (52.8)	264 (19.0)	732 (43.6)	193 (18.8)	38 (4.1)	1000 (72.9)	339 (30.5)	718 (56.7)	512 (39.7)	466 (47.2)	6216 (42.1)
Aware high blood pressure in pregnancy can be life threatening	793 (48.7)	584 (54.8)	552 (52.7)	232 (16.7)	736 (43.8)	189 (18.4)	37 (4.0)	968 (70.6)	232 (20.9)	626 (49.5)	511 (39.6)	466 (47.2)	5926 (40.1)

For peer review only

Table S2: Summary of birth preparedness by cluster [n (%)]

	Intervention Clusters						Control Clusters						Total
	**Hosur**	**Kanagala**	**Sangolli**	**Pattadakal lu**	**Amingad**	**Sutugunda r**	**Aihole**	**Galagali**	**Katageri**	**Belavadi**	**Chachadi**	**Ammangi**	
Composite	1556 (95.5)	1013 (95.1)	1044 (99.7)	869 (62.5)	466 (27.8)	639 (62.1)	702 (76.5)	878 (64.0)	827 (74.4)	1241 (98.0)	1289 (99.9)	932 (94.4)	11456 (77.5)
Arranged transport	1548 (95.0)	995 (93.4)	1044 (99.7)	1160 (83.5)	475 (28.3)	887 (86.2%)	720 (78.4)	1065 (77.7)	859 (77.3)	1262 (99.7)	1291 (100.0	848 (85.9)	12154 (82.2)
Has permission for emergency care	1626 (99.8)	1063 (99.8)	1045 (99.8)	1372 (98.7)	1634 (97.3)	855 (83.0)	909 (99.0)	1362 (99.3)	1015 (91.4)	1264 (99.8)	1291 (100.0)	984 (99.7)	14420 (97.5)
Has money saved for emergency	1324 (81.2	1008 (94.6	907 (86.6)	127 (9.1)	385 (22.9)	118 (11.4)	481 (52.4)	327 (23.9)	364 (32.8)	723 (57.1)	1168 (90.5	977 (99.0)	7909 (53.5)
Identified facility for delivery	1572 (96.5)	1058 (99.3)	1002 (95.7)	1011 (72.7)	1377 (82.0)	836 (81.2)	745 (81.2)	997 (72.7)	874 (78.7)	1210 (95.6)	1266 (98.1)	903 (91.5)	12851 (86.9)

CONSORT 2010 checklist of information to include when reporting a randomised trial*

Section/Topic	Item No	Checklist item	Reported on page No
**Title and abstract**			
	1a	Identification as a randomised trial in the title	Page 1
	1b	Structured summary of trial design, methods, results, and conclusions (for specific guidance see CONSORT for abstracts)	Page 2
**Introduction**			
Background and objectives	2a	Scientific background and explanation of rationale	Page 4
	2b	Specific objectives or hypotheses	Page 4
**Methods**			
Trial design	3a	Description of trial design (such as parallel, factorial) including allocation ratio	Page 4-5
	3b	Important changes to methods after trial commencement (such as eligibility criteria), with reasons	N/A*
Participants	4a	Eligibility criteria for participants	Page 5
	4b	Settings and locations where the data were collected	Page 5
Interventions	5	The interventions for each group with sufficient details to allow replication, including how and when they were actually administered	Page 5-6
Outcomes	6a	Completely defined pre-specified primary and secondary outcome measures, including how and when they were assessed	Page 7
	6b	Any changes to trial outcomes after the trial commenced, with reasons	N/A*

For peer review only

Sample size	7a	How sample size was determined	N/A*
	7b	When applicable, explanation of any interim analyses and stopping guidelines	N/A*
Randomisation:			
Sequence generation	8a	Method used to generate the random allocation sequence	Page 5
	8b	Type of randomisation; details of any restriction (such as blocking and block size)	Page 5
Allocation concealment mechanism	9	Mechanism used to implement the random allocation sequence (such as sequentially numbered containers), describing any steps taken to conceal the sequence until interventions were assigned	N/A*
Implementation	10	Who generated the random allocation sequence, who enrolled participants, and who assigned participants to interventions	N/A*
Blinding	11a	If done, who was blinded after assignment to interventions (for example, participants, care providers, those assessing outcomes) and how	N/A*
	11b	If relevant, description of the similarity of interventions	N/A*
Statistical methods	12a	Statistical methods used to compare groups for primary and secondary outcomes	Page 6
	12b	Methods for additional analyses, such as subgroup analyses and adjusted analyses	Page 6
**Results**			
Participant flow (a diagram is strongly recommended)	13a	For each group, the numbers of participants who were randomly assigned, received intended treatment, and were analysed for the primary outcome	N/A*
	13b	For each group, losses and exclusions after randomisation, together with reasons	N/A*
Recruitment	14a	Dates defining the periods of recruitment and follow-up	Page 5

For peer review only

	14b	Why the trial ended or was stopped	N/A*
Baseline data	15	A table showing baseline demographic and clinical characteristics for each group	Page 7-8, table 1
Numbers analysed	16	For each group, number of participants (denominator) included in each analysis and whether the analysis was by original assigned groups	Page 8-9, tables 2 and 3
Outcomes and estimation	17a	For each primary and secondary outcome, results for each group, and the estimated effect size and its precision (such as 95% confidence interval)	Page 8-9, tables 2 and 3
	17b	For binary outcomes, presentation of both absolute and relative effect sizes is recommended	N/A*
Ancillary analyses	18	Results of any other analyses performed, including subgroup analyses and adjusted analyses, distinguishing pre-specified from exploratory	N/A*
Harms	19	All important harms or unintended effects in each group (for specific guidance see CONSORT for harms)	N/A*
**Discussion**			
Limitations	20	Trial limitations, addressing sources of potential bias, imprecision, and, if relevant, multiplicity of analyses	Page 13
Generalisability	21	Generalisability (external validity, applicability) of the trial findings	Page 13
Interpretation	22	Interpretation consistent with results, balancing benefits and harms, and considering other relevant evidence	Page 12-13
**Other information**			
Registration	23	Registration number and name of trial registry	Page 4
Protocol	24	Where the full trial protocol can be accessed, if available	N/A*

For peer review only

Funding	25	Sources of funding and other support (such as supply of drugs), role of funders	Page 14

*For more details on the methodology, implementation and results of the full CLIP Trial in India, please see: Bellad MB, Goudar SS, Mallapur AA, Sharma S, Bone J, Charantimath US, Katageri GM, Ramadurg UY, Mark Ansermino J, Derman RJ, Dunsmuir DT, Honnungar NV, Karadiguddi C, Kavi AJ, Kodkany BS, Lee T, Li J, Nathan HL, Payne BA, Revankar AP, Shennan AH, Singer J, Tu DK, Vidler M, Wong H, Bhutta ZA, Magee LA, von Dadelszen P; CLIP India Working Group. Community level interventions for pre-eclampsia (CLIP) in India: A cluster randomised controlled trial. Pregnancy Hypertens. 2020 Jul;21:166-175. doi: 10.1016/j.preghy.2020.05.008. Epub 2020 May 19. PMID: 32554291; PMCID: PMC7471838.

For peer review only

BMJ Open

Community engagement for birth preparedness and complication readiness in the Community Level Interventions for Pre-eclampsia (CLIP) trial in India: A mixed method evaluation

Journal:	*BMJ Open*
Manuscript ID	bmjopen-2021-060593.R1
Article Type:	Original research
Date Submitted by the Author:	14-Sep-2022
Complete List of Authors:	Kavi, Avinash; KLE Academy of Higher Education and Research, Community Medicine and Women's and Children's Health Research Unit<br>Kinshella, Mai-Lei Woo; The University of British Columbia Faculty of Medicine, Department of Obstetrics and Gynaecology<br>Ramadurg, Umesh Y.; S Nijalingappa Medical College, Women’s and Children’s Health Research Unit<br>Charantimath, Umesh; KLE Academy of Higher Education and Research, Women’s and Children’s Health Research Unit<br>Katageri, Geetanjali M.; S Nijalingappa Medical College, Women’s and Children’s Health Research Unit<br>Karadiguddi, Chandrashekhar C.; KLE Academy of Higher Education and Research<br>Honnungar, Narayan V.; KLE Academy of Higher Education and Research<br>Bannale, Shashidhar G.; S Nijalingappa Medical College, Women’s and Children’s Health Research Unit<br>Mungarwadi, Geetanjali I.; KLE Academy of Higher Education and Research<br>Bone, Jeffrey; The University of British Columbia, Obstetrics and Gynecology<br>Vidler, Marianne; The University of British Columbia, Obstetrics & Gynaecology<br>Magee, Laura; King's College London School of Medical Education,<br>Mallapur, Ashalata; BVVS S Nijalingappa Medical College and HSK Hospital and Research Centre<br>Goudar, Shivaprasad S.; KLE University, Women's and Children's Health Research Unit<br>Bellad, Mrutyunjaya; KLE Academy of Higher Education and Research, Women’s and Children’s Health Research Unit<br>Derman, Richard ; Thomas Jefferson University<br>von Dadelszen, Peter ; King's College London Faculty of Life Sciences and Medicine, Department of Women and Children's Health<br>The CLIP India Working Group, .; The University of British Columbia Faculty of Medicine, Department of Obstetrics and Gynaecology
**Primary Subject Heading**:	Obstetrics and gynaecology
Secondary Subject Heading:	Public health

For peer review only - http://bmjopen.bmj.com/site/about/guidelines.xhtml

Keywords:	OBSTETRICS, Public health < INFECTIOUS DISEASES, Hypertension < CARDIOLOGY

SCHOLARONE™
Manuscripts

I, the Submitting Author has the right to grant and does grant on behalf of all authors of the Work (as defined in the below author licence), an exclusive licence and/or a non-exclusive licence for contributions from authors who are: i) UK Crown employees; ii) where BMJ has agreed a CC-BY licence shall apply, and/or iii) in accordance with the terms applicable for US Federal Government officers or employees acting as part of their official duties; on a worldwide, perpetual, irrevocable, royalty-free basis to BMJ Publishing Group Ltd ("BMJ") its licensees and where the relevant Journal is co-owned by BMJ to the co-owners of the Journal, to publish the Work in this journal and any other BMJ products and to exploit all rights, as set out in our licence.

The Submitting Author accepts and understands that any supply made under these terms is made by BMJ to the Submitting Author unless you are acting as an employee on behalf of your employer or a postgraduate student of an affiliated institution which is paying any applicable article publishing charge ("APC") for Open Access articles. Where the Submitting Author wishes to make the Work available on an Open Access basis (and intends to pay the relevant APC), the terms of reuse of such Open Access shall be governed by a Creative Commons licence – details of these licences and which Creative Commons licence will apply to this Work are set out in our licence referred to above.

Other than as permitted in any relevant BMJ Author's Self Archiving Policies, I confirm this Work has not been accepted for publication elsewhere, is not being considered for publication elsewhere and does not duplicate material already published. I confirm all authors consent to publication of this Work and authorise the granting of this licence.

For peer review only

Community engagement for birth preparedness and complication readiness in the Community Level Interventions for Pre-eclampsia (CLIP) trial in India: A mixed method evaluation

Avinash Kavi[1*]
Mai-Lei Woo Kinshella[2]
Umesh Y. Ramadurg[3]
Umesh S. Charantimath[1]
Geetanjali M. Katageri[3]
Chandrashekhar C. Karadiguddi[1]
Narayan V. Honnungar[1]
Shashidhar G. Bannale[3]
Geetanjali I. Mungarwadi[1]
Jeffrey N. Bone[2]
Marianne Vidler[2]
Laura A. Magee[4]
Ashalata A. Mallapur[3]
Shivaprasad S. Goudar[1]
Mrutyunjaya B. Bellad[1]
Richard J. Derman[5]
Peter von Dadelszen[4]
and the CLIP India Working Group

1. Women's and Children's Health Research Unit, KLE Academy of Higher Education and Research's Jawaharlal Nehru Medical College, Belagavi, Karnataka, India
2. Department of Obstetrics and Gynaecology and BC Children's Hospital Research Institute, University of British Columbia, Vancouver BC, Canada
3. Women's and Children's Health Research Unit, S. Nijalingappa Medical College, Bagalkote, Karnataka, India
4. Department of Women and Children's Health, School of Life Course Sciences, Kings College London, London, UK
5. Thomas Jefferson University, Philadelphia, USA

*Corresponding author: Avinash Kavi

Women's and Children's Health Research Unit, KLE Academy of Higher Education and Research's Jawaharlal Nehru Medical College, Belagavi, Karnataka, India

Email: dravinashkavi@gmail.com

Word count: 3850/4000

For peer review only

ABSTRACT

Objective: To describe the process of community engagement in northern Karnataka, India and its impact on pre-eclampsia knowledge, birth preparedness and complication readiness, pregnancy-related care seeking and maternal morbidity.

Design: This study was a secondary analysis of a cluster randomized trial of Community Level Interventions for Preeclampsia (CLIP). A total of 12 clusters based on primary health centre catchment areas were randomised to intervention or control. Community engagement was conducted in intervention clusters. Community engagement attendance was summarized according to participant group using both quantitative and qualitative assessment. Pre-eclampsia knowledge, birth preparedness, health services engagement and perinatal outcomes was evaluated within trial surveillance. Outcomes were compared between trial arms using a mixed effects logistic regression model on RStudio (RStudio Inc, Boston, United States). Community feedback notes were thematically analysed on NVivo12 (QSR International, Melbourne, Australia).

Setting: Belagavi and Bagalkote districts in rural Karnataka, India.

Participants: Pregnant women and women of reproductive age, mothers and mothers-in-law, community stakeholders and male household decision-makers, and health workers.

Results: A total of 1,379 community engagement meetings were conducted with 39,362 participants between November 2014 and October 2016. Community engagement activities may have had an effect on modifying community attitudes towards hypertension in pregnancy and its complications. However, rates of pre-eclampsia knowledge, birth preparedness, health services engagement and maternal morbidities among individual pregnant women were not significantly impacted by community engagement activities in their area.

Conclusion: Evaluation of our community engagement program in India demonstrates the feasibility of reaching pregnant women alongside household decision-makers, community stakeholders and health workers. More research is needed to explore the pathways of impact between broad community mobilization to strengthen support for maternal care-seeking and clinical outcomes of individual pregnant women.

Trial registration: NCT01911494

Keywords: Community Engagement, Birth preparedness, Complication readiness, Pre-eclampsia, Community support, Barriers.

For peer review only - http://bmjopen.bmj.com/site/about/guidelines.xhtml

STRENGTHS AND LIMITATIONS OF THIS STUDY

- We systematically evaluated the effectiveness of a large-scale community engagement program conducted with the Community Level Interventions for Pre-eclampsia (CLIP) Trial in rural India using a process evaluation framework. The logic model outlines the hypothesized relationships between inputs, outputs and outcomes of interest to explore potential pathways of change.
- Data for the assessment was obtained from a prospective population-based surveillance system of women in the CLIP Trial and community engagement records including community feedback notes.
- The community engagement program and its evaluation involved a wide range of health workers, pregnant women, household decision-makers and community leaders.
- The limited timeframe within a clinical trial may have been too short to appropriately evaluate community-level behavioural change and more research is needed to better understand the influence of external factors on maternal health care-seeking.
- Community engagement was one of the components of a complex intervention evaluated in the CLIP Trial and interventions involving multiple factors makes the impact assessment more complicated.

For peer review only

For peer review only - http://bmjopen.bmj.com/site/about/guidelines.xhtml

INTRODUCTION

Over 94% of maternal deaths occur in low- and middle-income countries (LMICs), primarily in South Asia and sub-Saharan Africa, and are preventable (1,2). Maternal deaths relate primarily to delays in triage (ability of care provider and women to identify who is severely ill and requires urgent care), transport (ability to get women to appropriate care when needed) and treatment (ability to provide appropriate treatment when care accessed) (3). Increased birth preparedness and complication readiness can help mitigate against delays and ensure timely identification of the need for seeking skilled care and arrival at the appropriate facility for pregnancy complications. India has an estimated maternal mortality ratio (MMR) of 113 per 100,000 livebirths in 2016-18, with pregnancy hypertension accounting for 7% of maternal deaths (4). While India has achieved a 6.3% average annual rate of decline from 1997 to 2018, progress has varied by region (4). Though the states in southern India are often categorized as high-performing with better maternal and perinatal health indicators than much of the country, the MMR in Karnataka is double the rate in neighbouring Kerala (92 vs 42 per 100,000 live births) (4).

Birth preparedness and complication readiness (BPCR) interventions are strongly recommended by the World Health Organization (WHO) to increase skilled birth attendance and timely use of facility care for obstetric and neonatal complications (5,6). According to the WHO, BCPR involves counselling pregnant women and their families on knowledge of pregnancy danger signs and developing a BCPR plan that includes identification of health facility and transport for birth and in case of complications, identification of a birth companion and preferred birth attendant, supplies to bring to the facility, funds for any expenses, and identification of a support person to look after other children at home as well as compatible blood donors if needed (5–7). involvement of community members are critical in the implementation of community health programs (8). In India, raising community awareness to support BPCR complements the national programme of community health workers called Accredited Social Health Activists (ASHAs), who aim to connect women and children to primary care (9). ASHAs identify pregnant women in their communities and accompany them to nearby primary health centre for registration and antenatal care (ANC) visits. However, previous research in Karnataka revealed that pregnant women rarely made health care decisions on their own, and other family members and community leaders were highly influential in decision-making (10). Bringing community members together to raise awareness of maternal health issues mobilize wider support for maternal healthcare seeking and ASHAs' work in local communities.

Over the past few decades, community engagement (CE) has emerged as an increasingly effective strategy for harnessing community potential for health improvement (11–16). CE is defined as 'a process of working collaboratively with groups of people who are affiliated by geographic proximity, special interests, or similar situations, with respect to issues affecting their well-being' (17). It is a dynamic relational process that facilitates communication, interaction, involvement, and

For peer review only - http://bmjopen.bmj.com/site/about/guidelines.xhtml

exchange between an organization and a community for a range of social and organizational outcomes (18). This strategy has been used for health promotion, research, and policy making to address a variety of health issues (19–22). CE enables a more contextualized understanding of the perceptions and contexts of the members of the community, thereby facilitating stronger relationships. Implementation of CE enhances the delivery of the health care services in an optimal manner for mothers and newborns, which ultimately aligns with the vision of national goal.

This paper is the secondary analysis of the Community Level Interventions for Pre-eclampsia (CLIP) Trial, India; the primary paper has been published elsewhere (23).The paper describes the process of CE, one of the component of CLIP Trial, and evaluates the impact of CE on knowledge around pre-eclampsia, birth preparedness and complication readiness, pregnancy-related care seeking and rates of maternal morbidity.

METHODS

The CLIP Trial and study setting

The CLIP India Trial (Clinical Trials.gov NCT01911494) was a community-based cluster randomised controlled trial reported according to the CONSORT 2010 Statement (**Table S1 checklist**). The CLIP India Trial was conducted in rural Karnataka, India where the incidence of pregnancy hypertension is 10.3% (24). This study was conducted in 12 distinct geographic regions in Karnataka State, six in each Belagavi and Bagalkote districts (**Figure 1 map**) (**Figure 2 map**). Belagavi has a population of 4,779,661 living in 1278 villages and Bagalkote has a population of 1,889,752 living in 627 villages (25). There are an estimated 500 birth annually in each district (25). The adult female literacy rate varies in the two districts, at 70.2% in Belagavi, and 59.3% in Bagalkote, respectively (26).

The primary health centre (PHC) and its catchment area served as the unit of randomization. Clusters were chosen according to region, accessibility for surveillance, and absence of conflicting research activity. Clusters were allocated (TL, statistician) using a restricted randomisation algorithm, balancing region and population size to ensure equivalence between intervention and control clusters. tratified randomisation was undertaken to allocate six clusters each to the intervention and control groups, using population size as a single stratification factor. Both intervention and control clusters were similar in terms of population (11.6 per hectare in intervention clusters vs 13.3 per hectare in control clusters) and community health workers (17 vs 18 ASHAs per cluster) density (23). Among the pregnant women enrolled in the CLIP Trial (7,839 in intervention clusters, 6,944 in the control clusters), maternal age was on average 22-23 years old, 56-58% of women had 8 or more years of schooling, and populations were over 90% Hindu without significant difference between intervention and control clusters (23).

For peer review only - http://bmjopen.bmj.com/site/about/guidelines.xhtml

The overall CLIP Trial focused on implementing community-level evidence-based care to reduce all cause maternal and perinatal mortality and major morbidity by supporting early identification and prompt referrals for pregnancy complications. The intervention included community engagement alongside strengthening clinical capacities of ASHAs to assess blood pressure and usage of PIERS-On-the-Move (POM). POM is a mobile-based application decision aid for community health workers to identify women at risk of adverse outcomes from pregnancy hypertension and guide management (27,28). More detail on the overall CLIP India Trial, participant characteristics, and reporting on the primary outcomes have been published elsewhere (23).

Process of facilitating community engagement in the CLIP Trial

The CE component was carried out in intervention clusters over the entire trial period (November 2014 - October 2016). The CE activities aimed at creating culturally and contextually appropriate discussion to improve maternal health awareness and action around the prevention of maternal morbidity and mortality. CE aimed to improve birth preparedness, nutrition, and promote appropriate ANC and healthcare seeking. Topics included warning signs of pregnancy complications, permission to seek care, identifying a skilled birth attendant, facility for delivery, and mode of transport as well as saving up funds for transport and treatment. Discussions also included adverse pregnancy outcomes and success stories in the community, overcoming local barriers to accessing maternal health care and the interventions in the CLIP Trial.

Community engagement meetings were primarily held at the anganwadi (AW) centres in the village, which offer basic health services. These were locations where pregnant women and children routinely access ANC, immunizations and other maternal and child health care services. Meetings were also held at sub-centres, PHCs, temples, public halls, schools or other appropriate nearby locations for large monthly sessions.

The activities convened multiple levels of the local health care system. This included workers (AWW) at the AW centres, ASHAs who worked in the villages, Auxiliary Nurse Midwives (ANMs) who worked at sub-centres, Lady Health Visitors (LHV) who supervised the ANMs, and medical officers who coordinated the PHC. In addition, ASHA supervisors and Registry Administrators (RAs) appointed as a part of the trial attended meetings.

Pregnant women and married women of reproductive age groups were primary beneficiaries while their family members and other household decision-makers were secondary targets. These secondary targets were involved in CE activities because household decision-makers were often in charge of women's decisions to seek care and community leaders provided advice and financial assistance for healthcare seeking at times. Community leaders included elected representatives and teachers.

The CE activity schedule was prepared at the beginning of each month by the local study team and shared to the respective community stakeholders. Meetings lasted between 30 and 150 minutes. These meetings were facilitated by the study research

For peer review only - http://bmjopen.bmj.com/site/about/guidelines.xhtml

officers with support from the medical officers at each PHC. Sessions began with a short formal introduction and description of the purpose, then discussion of CE topics prompted by informational posters developed for the project (for example, see **Figure S1**). Participants were encouraged to actively participate, share personal experiences and discuss case examples. There were no monetary incentives for participants for attending the meeting. Participants were provided with nutritional snacks and fruits.

Data collection and analysis

For the purpose of assessing implementation, study staff and supervisors completed CE logs after each meeting. This included a quantitative dataset on the number of participants attending each meeting and target groups reached, as well as a qualitative dataset comprised of community feedback. For assessing the potential impact of CE activities on health outcomes, quantitative data was collected from the prospective population-based CLIP Trial Surveillance system of pregnant women enrolled in the intervention and control groups of the study (23,29). Data collected from the CLIP Trial Surveillance included demographic factors, birth preparedness, care-seeking, obstetric information, pre-eclampsia knowledge, perinatal outcomes and treatment information. Data forms were reviewed for completeness and entered weekly into the local database. De-identified and encrypted data were transferred to the central server for analyses.

We employed a convergent mixed-methods approach, which involves quantitative and qualitative data collection separately and integrating them at the point of interpretation to highlight the areas where the two datasets converge (30). **Figure 3** illustrates the strategy for assessing CE in the CLIP India Trial, based on the process evaluation framework for the CLIP Pilot Trial in Nigeria (31).

- **CE attendance** was summarized according to participant group: pregnant women and other women of reproductive age, the pregnant women's mothers and mothers-in-law, community stakeholders (community leaders, male and female decision-makers), health workers (AWW, ANM, ASHA, ASHA supervisor, LHV, medical officers, nurses, RA) and others (family members, neighbours, friends, community members).
- **Pre-eclampsia knowledge** was based on the clinical symptoms that predicted adverse maternal and perinatal outcomes in women with pre-eclampsia and included evaluating awareness of: 1) high blood pressure during pregnancy 2) abnormal bleeding in pregnancy , 3) seizures in pregnancy, 4) that pregnancy hypertension can be life threatening (32). The composite pre-eclampsia knowledge score included recalling at least one of the above conditions and at least four symptoms of high blood pressure in pregnancy.
- **Birth preparedness** evaluated a pregnant woman's plans for: 1) transport in case of emergencies, 2) permission to seek emergency care, 3) money saved for an emergency, 4) identified health facility for delivery, and 5) at least two of the components for the BCPR composite score.

For peer review only - http://bmjopen.bmj.com/site/about/guidelines.xhtml

Pre-eclampsia knowledge, birth preparedness, health services engagement and perinatal outcomes indicators were summarized between arms with counts and frequencies for categorical variables and medians and interquartile range [IQR] for continuous variables. For health service questions, we restricted the denominators to women who delivered by trial end as these indicators are more likely to occur later in pregnancy. As pre-eclampsia knowledge and birth preparedness were asked throughout pregnancy and all women received community engagement, we included all women in assessing these rates. Outcomes were further compared between trial arms using a mixed effects logistic regression model (to account for clustering) with a logit link. Models were adjusted for age, maternal education, husband education, gestational age at booking, and parity and results are presented as odds ratios and 95% confidence intervals. Quantitative data were analysed using RStudio (RStudio Inc, Boston, United States), which was complemented by qualitative community feedback recorded by study staff. The community feedback notes were imported to NVivo12 software (QSR International, Melbourne, Australia) for thematic analysis.

Research ethics approval

The project received ethics committee approvals from KLE Academy of Higher Education and Research Deemed-to-be-University (MDC/IECHSR/2011-12/A-4; ICMR 5/7/859/12-RHN) and the University of British Columbia (UBC, H12-03497).

Patient and public involvement

Neither patients nor public were involved in the design or conduct of the study.

RESULTS

Community engagement attendance

A total of 1,379 CE meetings were conducted the 6 intervention clusters. Among them 586 sessions were conducted in Belagavi district and 773 sessions at Bagalkote district. The median number of sessions per cluster was 204 [IQR 186 – 281]. **Table 1** describes the activities across clusters and the participants reached. As long as pregnant women remained in the same village, they were encouraged to attend all sessions. The other participants, such as stakeholders and decision-makers, were usually different for each session. Pregnant women were most represented at the meetings, followed by mothers-in-law who often accompanied them. Husbands and fathers-in-law were least represented in the meetings as they often had competing priorities; however, overall sessions were well attended by target groups.

For peer review only - http://bmjopen.bmj.com/site/about/guidelines.xhtml

Table 1: Participants reached in CE activities in the intervention clusters

Target groups	Number of participants (% of total)	Number of sessions (% of total)
Pregnant women and other women of reproductive age	22,149 (56.3%)	1,379 (100.0%)
Mothers and mothers-in-law	4,643 (11.8%)	1,140 (82.7%)
Community stakeholders	5,685 (14.4%)	1,147 (83.2%)
Health workers	4,752 (12.1%)	1,376 (99.8%)
Others	2,113 (5.4%)	N/A
Total	39,342	1,379

Impact on clinical outcomes

Tables 2 and **3** present summaries of pre-eclampsia knowledge and birth preparedness among pregnant women in the CLIP Trial. The rates for both (composite and components) were not significantly different between arms. Only a minority of participants from both intervention and control (2-6%) could name at least four symptoms of high blood pressure in pregnancy or had overall pre-eclampsia knowledge. In contrast, there were higher levels of birth preparedness among women in both intervention and control with a majority of women reporting that they had arranged transport, has permission for emergency care, and identified a health facility for delivery. Only saving funds for an obstetric emergency was lower, with about half of women declaring that they had funds saved up. Confidence intervals of odd ratios are wide, reflecting high heterogeneity between study clusters [see **Tables S2 and S3**].

Table 2: Summary of pre-eclampsia knowledge by arm

	**Intervention** (n= 7,839)	**Control** (n= 6,944)	**Total*** (n=14,783)	**Adjusted odds ratio (95%CI);**	**p-value†**
Women can have abnormal bleeding in pregnancy	1,981 (25.3%)	2,368 (34.1%)	4,349 (29.4%)	0.88 (0.25, 3.18);	0.85
Women can have seizure in pregnancy	1,814 (23.1%)	1,691 (24.4%)	3,505 (23.7%)	1.49 (0.40, 5.62);	0.55
Women can have high blood pressure in pregnancy	3,143 (40.1%)	3,073 (44.3%)	6,216 (42.1%)	1.02 (0.32, 3.25);	0.97
High blood pressure in pregnancy can be life threatening	3,086 (39.4%)	2,840 (40.9%)	5,926 (40.1%)	0.86 (0.17, 4.21);	0.85

For peer review only - http://bmjopen.bmj.com/site/about/guidelines.xhtml

Can name at least four symptoms	441 (5.6%)	153 (2.2%)	594 (4.0%)	2.35 (0.60, 9.12);	0.22
Composite††	439 (5.6%)	151 (2.2%)	590 (4.0%)	2.35 (0.61, 9.10);	0.22

* *All pregnancies enrolled in the CLIP India Trial*

† *Estimated by mixed effects logistic regression adjusted for: maternal age, maternal education, husband education, gestational age at enrolment, parity, and cluster (random effect).*

††*Recall at least one condition and at least four symptoms of high blood pressure*

Table 3: Summary of birth preparedness by arm

	**Intervention** (n= 7,839)	**Control** (n= 6,944)	**Total** (n=14,783)	**Adjusted odds ratio (95%CI);**	**p-value***
Arranged transport	6,109 (77.9%)	6,045 (87.1%)	12,154 (82.2%)	0.40 (0.03, 6.07);	0.51
Has permission for emergency care	7,595 (96.9%)	6,825 (98.3%)	14,420 (97.5%)	0.48 (0.05, 4.31);	0.51
Has money saved for emergency	3,869 (49.4%)	4,040 (58.2%)	7,909 (53.5%)	0.46 (0.05, 4.19);	0.49
Identified facility for delivery	6,856 (87.5%)	5,995 (86.3%)	12,851 (86.9%)	1.50 (0.36, 6.27);	0.58
Composite†	5,587 (71.3%)	5,869 (84.5%)	11,456 (77.5%)	0.50 (0.05, 5.59);	0.58

**All pregnancies enrolled in the CLIP India Trial*

† *Estimated by mixed effects logistic regression adjusted for: maternal age, maternal education, husband education, gestational age at enrolment, parity, and cluster (random effect).*

††*Having at least two of the components of birth preparedness ready.*

Table 4 illustrates that health services engagement by pregnant women in the CLIP Trial were similar between arms. Almost all women had at least one ANC visit and three out of four women had at least four visits. Approximately one in ten women visited the PHC for reason other than routine care and about 3% of women were admitted to a health facility for reasons other than delivery, staying a median of three days. One in twenty women experienced a maternal morbidity and there was no significant difference between arms (OR 0.95, 95%CI: 0.61-1.50, p= 0.84).

Table 4: Health services engagement and maternal morbidity by arm

	**Intervention** (N=6,908)	**Control** (N=6,109)	**Total*** (N=13,017)	**Adjusted odds ratio (95%CI);**	**p-value†**
Women with at least one ANC visit	6,891 (99.8%)	6,068 (99.3%)	12,959 (99.6%)	Cannot be computed	-

For peer review only - http://bmjopen.bmj.com/site/about/guidelines.xhtml

Women with 4 or more ANC visits	5,140 (74.4%)	4,745 (77.7%)	9,885 (75.9%)	0.86 (0.51, 1.44);	0.57
Visited PHC for other than routine care	823 (11.9%)	739 (12.1%)	1562 (12.0%)	1.92 (0.43, 8.55);	0.39
Visited higher level facility for other than routine care	1,069 (15.5%)	780 (12.8%)	1849 (14.2%)	1.59 (0.58, 4.38);	0.37
Number of women admitted to health facility for reasons other than delivery	225 (3.3%)	170 (2.8%)	395 (3.0%)	1.28 (0.08, 19.3);	0.86
Median days of admission [IQR]	3.0 [2.0; 5.0]	3.0 [2.0; 5.0]	3.0 [2.0; 5.0]	-	-
Maternal morbidity††	371/7,839 (4.7%)	325/6,944 (4.7%)	696/14,783 (4.7%)	0.95 (0.61, 1.50);	0.84

**Pregnancies enrolled in the CLIP India Trial with postpartum follow-up*

† Estimated by mixed effects logistic regression adjusted for: maternal age, maternal education, husband education, gestational age at enrolment, parity, and cluster (random effect).

††Data taken from CLIP India primary trial paper and includes different adjustment factors (33)

Community feedback on community engagement activities

<u>Engaging family members</u>

Staff noted that communities appreciated engaging household decision-makers and community stakeholders alongside pregnant women and health workers. Household decision-makers may not have been aware of pre-eclampsia and other maternal health risks. Their inclusion in the discussion supported care for pregnant women and increased value in attending ANC.

> "The people appreciated these community engagement meetings. They said that they learnt many things about the care of the pregnant women, which they were not aware of earlier. The pregnant women became more aware about their health and their parents also started giving more support and care to her."

> "Earlier, it was difficult for the woman to take permission from the decision makers in the family to seek antenatal care. But after conducting the community engagement meetings, the views of the decision-makers were changed so that the seeking of permission for antenatal care became easy."

For peer review only - http://bmjopen.bmj.com/site/about/guidelines.xhtml

Mobilizing community support for maternal health

CE feedback indicated increased community support for BPCR. There were examples of community leaders advocating for appropriate maternal health care seeking with household decision-makers as well as pushing for change to support local health systems.

> “The views of stakeholders also changed so much that the arrangement of transport, financial support, counselling the decision-makers became effective. For example, in the initial period of the study, there was one incident where the husband, the male decision-maker of the family, of the woman with eclampsia refused to take her to the hospital but after counselling by the community leaders and his family doctor, he agreed to take her to the higher care hospital.”

> “The community leaders recognized the significant role played by the ASHAs and the PHC personnel in promoting the health of the pregnant women. The PHC did not have an ambulance of its own and the community leaders saw to it that a new ambulance was made available at the PHC. They also built a room above the PHC building as a meeting place for the AHSAs to continue the work actively.”

External factors and barriers

Barriers of poverty, health infrastructure gaps and poor quality of care at facilities were frequently highlighted in CE discussions.

> “People accepted all the topics. They only found the issue of arranging money to be a problem because they had meagre earnings.”

Additionally, the cost of treatment at private hospitals was often brought up in discussions in conjunction with the lack of services at government facilities. Some participants pointed out that there was no obstetric specialist at a nearby secondary level hospital for a period of time. Staff reflected that community members sometimes highlighted the need to strengthen care at facilities in addition to raising community awareness.

> “There were some suggestions from the community about the improvement of services at the local government health centres.”

At referral facilities, participants reported that they often had to wait a long time before they would be provided services. They also reported that rural, uneducated people may encounter stigma and discrimination when seeking care at urban tertiary facilities.

> “The participants wanted to know how to get immediate admission and care at higher and bigger tertiary care hospitals.”

For peer review only - http://bmjopen.bmj.com/site/about/guidelines.xhtml

> "They also demanded about early admission and prompt service at the tertiary care hospitals as there are some uneducated persons in the community who face some problems in getting the services in bigger hospitals."

DISCUSSION

Summary of findings and comparisons with the literature

Our evaluation of the CLIP CE program in India demonstrates the feasibility of reaching pregnant women alongside household decision-makers, community stakeholders and health workers. This initiative involved almost 40,000 participants in 1,379 sessions over a period of two years. Engaging household decision-makers and community leaders together with pregnant women to discuss pregnancy care may have helped to shift local opinions on the value of ANC to monitor for pregnancy complications and mitigate risks. However, rates of pre-eclampsia knowledge, birth preparedness, health services engagement and maternal morbidities remained similar across arms in the CLIP Trial. According to our evaluation framework (**Figure 3**), our study found no evidence that the outputs were associated with our expected outcomes.

Although research findings related to the effect of community participation to improve access to maternal health care services have been mixed (34), a previous study in Uttar Pradesh in Northern India found significant effects of community health worker home visits and monthly community meetings on improved self-recognition of problems faced during pregnancy, birth preparedness components and knowledge of pregnancy danger signs (35). Differing outcomes between our findings and the study in Uttar Pradesh highlight two issues: first, the importance of context, and secondly, challenges in measuring impacts of community mobilization. Uttar Pradesh has much higher maternal mortality rates than Karnataka (36), suggesting that CE may have a stronger effect where there is lower baseline access to maternal health services. Secondly, in contrast to targeted community meetings with traditional maternal-care providers and birth attendants, our CE strategy was focused on broader community mobilization and strengthening support for maternal care-seeking among community stakeholders, which has previously been discussed in the literature as challenging to measure (8,37).

Community engagement in context and potential implications for policy and practice

CE involved shifting opinions on maternal health and cultures of care, behavioural change and mobilization and can take more time than the limited timeframe within a clinical trial. Logic models can help to understand the pathways of change, however, some of our expected short-term effects in birth preparedness via community mobilization may take more time than measured during the trial. A systematic review found increased knowledge of birth preparedness and complication readiness as a result of interventions but increased knowledge did not always correspond to increased utilization of maternal health services (38). While the CE program was successful in

For peer review only - http://bmjopen.bmj.com/site/about/guidelines.xhtml

reaching a wide audience of participants and community feedback suggested increased support for maternal health care-seeking, these may be upstream indicators or insufficient to change clinical outcomes.

A systematic review on the effects of community participation on improving uptake of skilled maternal and newborn care highlighted the importance of qualitative research to understand unforeseen impacts and socio-political factors (34). While quantitative results did not reveal an effect in our study, staff qualitatively noted that effects were noticeable in the opinions of community leaders and household decision-makers in their support for pregnancy care. Additionally, the discrepancy between the quantitative and qualitative results may also highlight the influence of external factors where community members gained appreciation of maternal health issues but were limited in their capacity to access care. Poverty and quality of care available at local health facilities emerged as significant barriers shared in community feedback, which is in line with previous research on the determinants of maternal health care services utilization in the area (10).

Nevertheless, qualitative findings that suggested improved awareness and attitudes towards pregnancy hypertension and its complications among community leaders and household decision-makers is promising. Previous research in the area found lack of knowledge around pre-eclampsia and eclampsia (39). Hypertension specifically as a pregnancy condition was unknown and there was no specific terminology in the local language (Kannada) for pre-eclampsia. CE has significant repercussions for the implementation of health care interventions and policy making.

Strengths and limitations

Strengths of our CE program and assessment include its systematic evaluation using a logic model framework and involvement of a wide range of health workers, pregnant women, household decision-makers and community leaders. Our study was also able to triangulate qualitative and quantitative datasets and data collected both

However, as a secondary analysis, our evaluation of the impact of CE activities was limited. The effects of CE on community leaders and household decision-makers were not adequately captured in our evaluation framework that focused on measuring effects with only the pregnant women enrolled in the trial. Additionally, the possibility of contamination cannot be ruled out as the clusters belonged to the same district. The health workers from the intervention and control clusters often meet at the district-level meetings and might exchange ideas and discuss the interventions. This would be a general limitation for cluster randomized settings. Furthermore, since CE was one of the components of a complex intervention, the effects assessed do not reflect CE alone. Intervention of multiple factors makes the impact assessment much more complicated.

For peer review only - http://bmjopen.bmj.com/site/about/guidelines.xhtml

CONCLUSION

Our study found that CE activities may have had an effect on modifying community attitudes towards hypertension in pregnancy and its complications but did not find an impact on individual women's pre-eclampsia knowledge, birth preparedness and care-seeking indicators. Contemplation of specific individual factors and community capabilities in engaging in the health seeking behaviours would contribute to the vision of supporting people-centred methodologies to deliver health promotion. Through the complex social interactions inherent in the current CE activities, appraisals over time will evaluate the effectiveness and sustainability of these interventions.

ACKNOWLEDGEMENTS

We would like to thank the women and their families who contributed data to this trial, in addition to the ASHA and ANMs who deliver care to the most vulnerable populations. Also, we would like to acknowledge the key contributions of the following DSMB members: Romano Nkumbwa Byaruhanga, Brian Darlow, Eileen Hutton (Chair), and Lehana Thabane.

We would also like to acknowledge the CLIP India Working Group members: Shashidhar G Bannale, Keval S Chougala, Vaibhav B Dhamanekar, Anjali M Joshi, Namdev A Kamble, Gudadayya S Kengapur, Uday S Kudachi, Sphoorthi S Mastiholi, Geetanjali I Mungarwadi, Esperança Sevene, Khátia Munguambe, Charfudin Sacoor, Eusébio Macete, Helena Boene, Felizarda Amose, Orvalho Augusto, Cassimo Bique, Ana Ilda Biz, Rogério Chiaú, Silvestre Cutana, Paulo Filimone, Emília Gonçálves, Marta Macamo, Salésio Macuacua, Sónia Maculuve, Ernesto Mandlate, Analisa Matavele, Sibone Mocumbi, Dulce Mulungo, Zefanias Nhamirre, Ariel Nhancolo, Cláudio Nkumbula, Vivalde Nobela, Rosa Pires, Corsino Tchavana, Anifa Vala, Faustino Vilanculo, Rahat N Qureshi, Sana Sheikh, Zahra Hoodbhoy, Imran Ahmed, Amjad Hussain, Javed Memon, Farrukh Raza, Olalekan O Adetoro, John O Sotunsa, Sharla K Drebit, Chirag Kariya, Mansun Lui, Diane Sawchuck, Ugochi V Ukah, Mai-Lei Woo Kinshella, Shafik Dharamsi, Guy A Dumont, Tabassum Firoz, Ana Pilar Betrán, Susheela M Engelbrecht, Veronique Filippi, William A Grobman, Marian Knight, Ana Langer, Simon A Lewin, Gwyneth Lewis, Craig Mitton, Nadine Schuurman, James G Thornton, France Donnay, Romano N Byaruhanga, Brian Darlow, Eileen Hutton, Mario Merialdi, Lehana Thabane, Kelly Pickerill.

DECLARATIONS OF INTEREST

The authors declare that they have no competing interests.

ROLE OF THE FUNDING SOURCE

This trial was funded by the University of British Columbia, a grantee of the Bill & Melinda Gates Foundation (PRE-EMPT initiative, grant number OPP1017337). Following input into trial design, the Gates Foundation had no role in data collection, analysis, or interpretation, or writing of the report.

For peer review only - http://bmjopen.bmj.com/site/about/guidelines.xhtml

AUTHOR CONTRIBUTIONS

PVD, RJD, MBB, SSG, AAM, LAM conceptualised the trial and components of the intervention. MBB, SSG, AAM, USC, GMK, UYR, AK, NVH, CCK, SGB, MV, GIM, implemented and co-ordinated monitoring of the trial. JNB and MWK performed the quantitative and qualitative analyses. AK and MWK wrote the first draft of the manuscript. All authors provided feedback and review of the manuscript.

DATA AVAILABILITY

The datasets used and/or analysed during the current study are available from the corresponding author on reasonable request.

FIGURE CAPTIONS

Figure 1. Study Location in India, Karnataka State

Figure 2. Belagavi and Bagalkote Districts, Karnataka State, India with detailed PHC areas.

Figure 3. A process evaluation plan for assessing community engagement to improve maternal health in Karnataka, India

ETHICAL APPROVAL

The project received ethics committee approvals from KLE Academy of Higher Education and Research Deemed-to-be-University (MDC/IECHSR/2011-12/A-4; ICMR 5/7/859/12-RHN) and the University of British Columbia (UBC, H12-03497).

For peer review only

REFERENCES

1. WHO, UNICEF, UNFPA, World Bank Group, The United Nations Population Division. Trends in maternal mortality 2000 to 2017. Geneva: World Health Organization; 2019.

2. Goldenberg RL, McClure EM, Saleem S. Improving pregnancy outcomes in low- and middle-income countries. Reproductive Health. 2018 Jun 22;15(Suppl 1).

3. Thaddeus S, Maine D. Too far to walk: maternal mortality in context. Social science & medicine (1982). 1994;38(8):1091–110.

4. Meh C, Sharma A, Ram U, Fadel S, Correa N, Snelgrove J, et al. Trends in maternal mortality in India over two decades in nationally representative surveys. BJOG. 2022 Mar;129(4):550–61.

5. World Health Organization. WHO recommendation on birth preparedness and complication readiness [Internet]. The WHO Reproductive Health Library. [cited 2022 Aug 24]. Available from: https://srhr.org/rhl/article/who-recommendation-on-birth-preparedness-and-complication-readiness

6. World Health Organization. WHO recommendations on health promotion interventions for maternal and newborn health [Internet]. Geneva: World Health Organization; 2015 [cited 2022 Aug 24]. Available from: https://www.who.int/publications/i/item/9789241508742

7. JHPIEGO. Monitoring Birth Preparedness and Complications Readiness: Tools and Indicators for Maternal and Newborn Health. Baltimore; 2004.

8. Cyril S, Smith BJ, Possamai-Inesedy A, Renzaho AMN. Exploring the role of community engagement in improving the health of disadvantaged populations: A systematic review. Global Health Action. 2015;8.

9. About Accredited Social Health Activist (ASHA) :: National Health Mission [Internet]. [cited 2022 Aug 24]. Available from: https://nhm.gov.in/index1.php?lang=1&level=1&sublinkid=150&lid=226

10. Vidler M, Ramadurg U, Charantimath U, Katageri G, Karadiguddi C, Sawchuck D, et al. Utilization of maternal health care services and their determinants in Karnataka State, India. Reproductive Health. 2016 Jun 8;13(1):37.

11. Lassi ZS, Kumar R, Bhutta ZA. Community-Based Care to Improve Maternal, Newborn, and Child Health. In: Disease Control Priorities, Third Edition (Volume 2): Reproductive, Maternal, Newborn, and Child Health. Washington DC: The World Bank; 2016. p. 263–84.

12. Ricca J, Kureshy N, Leban K, Prosnitz D, Ryan L. Community-based intervention packages facilitated by NGOs demonstrate plausible evidence for child mortality impact. Health Policy and Planning. 2014 Mar;29(2):204–16.

For peer review only - http://bmjopen.bmj.com/site/about/guidelines.xhtml

13. Rockers PC, Fink G, Zanolini A, Banda B, Biemba G, Sullivan C, et al. Impact of a community-based package of interventions on child development in Zambia: A cluster-randomised controlled trial. BMJ Global Health. 2016 Nov 1;1(3):104.

14. Prost A, Colbourn T, Seward N, Azad K, Coomarasamy A, Copas A, et al. Women's groups practising participatory learning and action to improve maternal and newborn health in low-resource settings: a systematic review and meta-analysis. The Lancet. 2013 May 18;381(9879):1736–46.

15. Sadruddin S, Khan I ul H, Bari A, Khan A, Ahmad I, Qazi SA. Effect of community mobilization on appropriate care seeking for pneumonia in Haripur, Pakistan. Journal of Global Health. 2015 Jun;5(1):010405.

16. Mochache V, Irungu E, El-Busaidy H, Temmerman M, Gichangi P. "Our voices matter": A before-after assessment of the effect of a community-participatory intervention to promote uptake of maternal and child health services in Kwale, Kenya 11 Medical and Health Sciences 1117 Public Health and Health Services. BMC Health Services Research. 2018 Dec 4;18(1).

17. Butterfoss FD, Francisco VT. Evaluating community partnerships and coalitions with practitioners in mind. Health promotion practice. 2004 Apr 30;5(2):108–14.

18. NHS Department of Health and Human Services. Principles of Community Engagement. Second edi. NIH Publication No. 11-7782. Bethesda, Maryland: National Institutes of Health (NIH); 2011.

19. Johnston KA. Toward a Theory of Social Engagement. In: Johnston KA, Taylor M, editors. The Handbook of Communication Engagement. Hoboken, NJ, USA: John Wiley & Sons, Inc.; 2018. p. 17–32.

20. National Institute for Health and Care Excellence (NICE). Community engagement: improving health and wellbeing and reducing health inequalities . London: NICE; 2016.

21. Balcázar HG, de Heer H, Rosenthal L, Aguirre M, Flores L, Puentes FA, et al. A Promotores de Salud intervention to reduce cardiovascular disease risk in a high-risk hispanic border population, 2005-2008. Preventing Chronic Disease. 2010;7(2).

22. Wells KB, Jones L, Chung B, Dixon EL, Tang L, Gilmore J, et al. Community-partnered cluster-randomized comparative effectiveness trial of community engagement and planning or resources for services to address depression disparities. Journal of General Internal Medicine. 2013;28(10):1268–78.

23. Bellad MB, Goudar SS, Mallapur AA, Sharma S, Bone J, Charantimath US, et al. Community level interventions for pre-eclampsia (CLIP) in India: A cluster randomised controlled trial. Pregnancy Hypertension. 2020 Jul 1;21:166–75.

24. Magee LA, Sharma S, Nathan HL, Adetoro OO, Bellad MB, Goudar S, et al. The incidence of pregnancy hypertension in India, Pakistan, Mozambique, and Nigeria: A prospective population-level analysis. PLOS Medicine. 2019;16(4):e1002783.

For peer review only - http://bmjopen.bmj.com/site/about/guidelines.xhtml

25. Government of India. Census of India 2011: Our Census, Our Future. Delhi: Office of The Registrar General & Census Commissioner;

26. International Institute for Population Sciences (IIPS). National Family Health Survey (NFHS-4), 2015-16: India. Mumbai; 2017.

27. Lim J, Cloete G, Dunsmuir DT, Payne BA, Scheffer C, von Dadelszen P, et al. Usability and Feasibility of PIERS on the Move: An mHealth App for Pre-Eclampsia Triage. JMIR mHealth and uHealth. 2015 Apr 17;3(2):e37.

28. Payne B, Dunsmuir D, Hall D, Lim J, Sikandar R, Magee L, et al. Designing an evidence-based mobile health decision aid for the management of hypertensive disorders of pregnancy (HDP) in low-resourced settings. BMC Health Services Research. 2014 Jul 7;14(S2):P93.

29. Goudar SS, Carlo WA, McClure EM, Pasha O, Patel A, Esamai F, et al. The maternal and newborn health registry study of the global network for women's and children's health research. International Journal of Gynecology and Obstetrics. 2012;118(3):190–3.

30. Fetters MD, Curry LA, Creswell JW. Achieving Integration in Mixed Methods Designs—Principles and Practices. Health Services Research. 2013 Dec 1;48(6pt2):2134–56.

31. Sharma S, Adetoro OO, Vidler M, Drebit S, Payne BA, Akeju DO, et al. A process evaluation plan for assessing a complex community-based maternal health intervention in Ogun State, Nigeria. BMC Health Services Research. 2017 Mar 28;17(1).

32. Yen TW, Payne B, Qu Z, Hutcheon JA, Lee T, Magee LA, et al. Using clinical symptoms to predict adverse maternal and perinatal outcomes in women with preeclampsia: data from the PIERS (Pre-eclampsia Integrated Estimate of RiSk) study. J Obstet Gynaecol Can. 2011 Aug;33(8):803–9.

33. Bellad MB, Goudar SS, Mallapur AA, Sharma S, Bone J, Charantimath US, et al. Community level interventions for pre-eclampsia (CLIP) in India: A cluster randomised controlled trial. Pregnancy Hypertension. 2020 Jul 1;21:166–75.

34. Marston C, Renedo A, McGowan CR, Portela A. Effects of Community Participation on Improving Uptake of Skilled Care for Maternal and Newborn Health: A Systematic Review. Braitstein P, editor. PLoS ONE. 2013 Feb 4;8(2):e55012.

35. Kumar V, Kumar A, Das V, Srivastava NM, Baqui AH, Santosham M, et al. Community-driven impact of a newborn-focused behavioral intervention on maternal health in Shivgarh, India. International Journal of Gynecology and Obstetrics. 2012;117(1):48–55.

36. Dandona L, Dandona R, Kumar GA, Shukla DK, Paul VK, Balakrishnan K, et al. Nations within a nation: variations in epidemiological transition across the states of India, 1990–2016 in the Global Burden of Disease Study. The Lancet. 2017 Dec 2;390(10111):2437–60.

For peer review only - http://bmjopen.bmj.com/site/about/guidelines.xhtml

37. MacQueen KM, Bhan A, Frohlich J, Holzer J, Sugarman J. Evaluating community engagement in global health research: The need for metrics Ethics in Biomedical Research. BMC Medical Ethics. 2015 Jul 1;16(1):44.

38. Solnes Miltenburg A, Roggeveen Y, Shields L, van Elteren M, van Roosmalen J, Stekelenburg J, et al. Impact of Birth Preparedness and Complication Readiness Interventions on Birth with a Skilled Attendant: A Systematic Review. Niederkrotenthaler T, editor. PLOS ONE. 2015 Nov 23;10(11):e0143382.

39. Vidler M, Charantimath U, Katageri G, Ramadurg U, Karadiguddi C, Sawchuck D, et al. Community perceptions of pre-eclampsia in rural Karnataka State, India: A qualitative study. Reproductive Health. 2016 Jun 8;13(1):35.

For peer review only

For peer review only - http://bmjopen.bmj.com/site/about/guidelines.xhtml

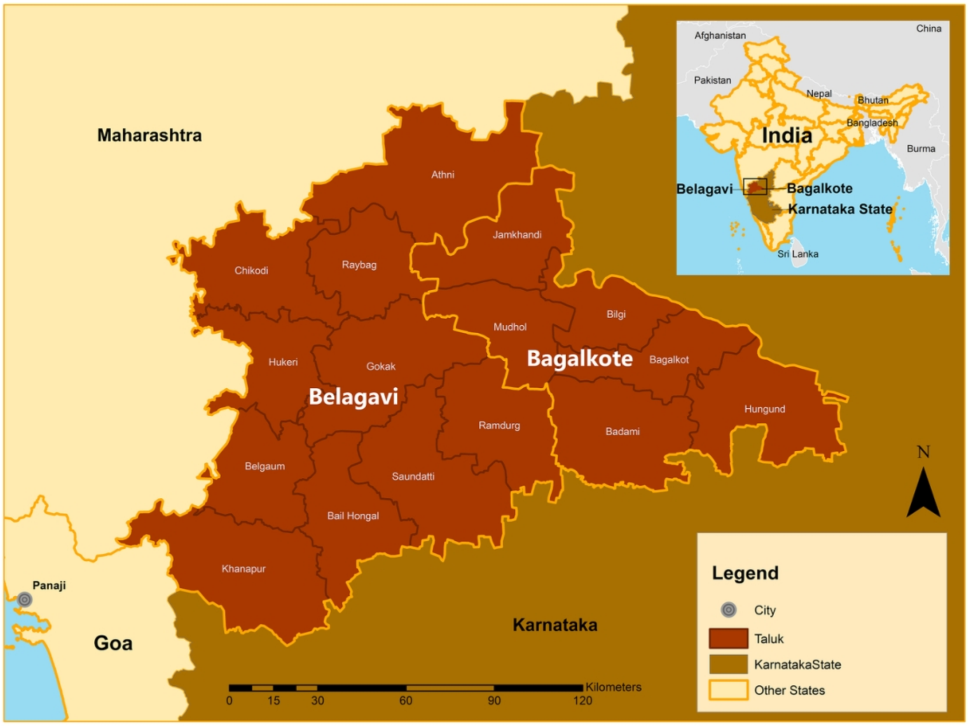

Study Location in India, Karnataka State

59x45mm (600 x 600 DPI)

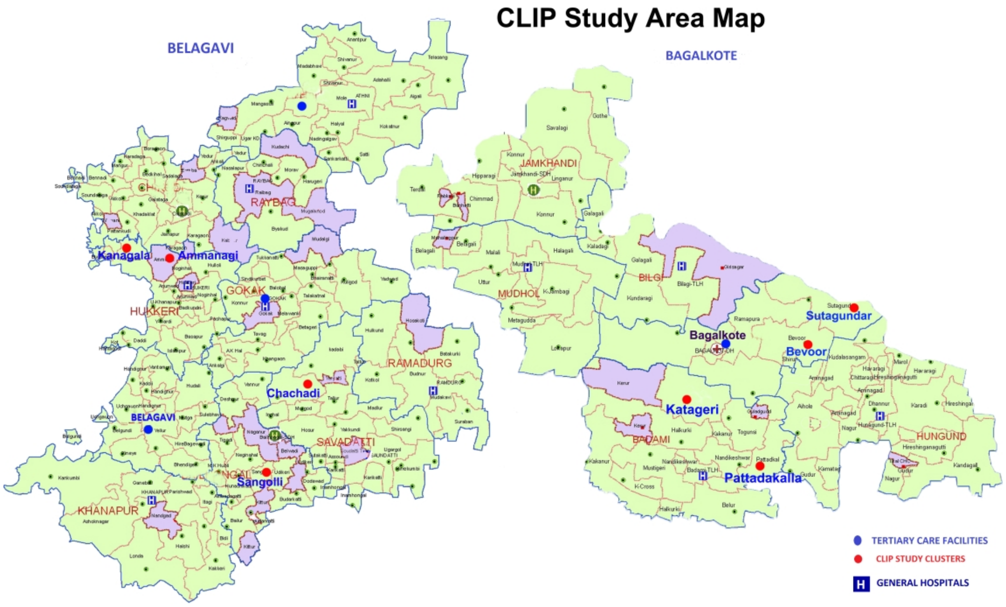

Belagavi and Bagalkote Districts, Karnataka State, India with detailed PHC areas.

793x474mm (144 x 144 DPI)

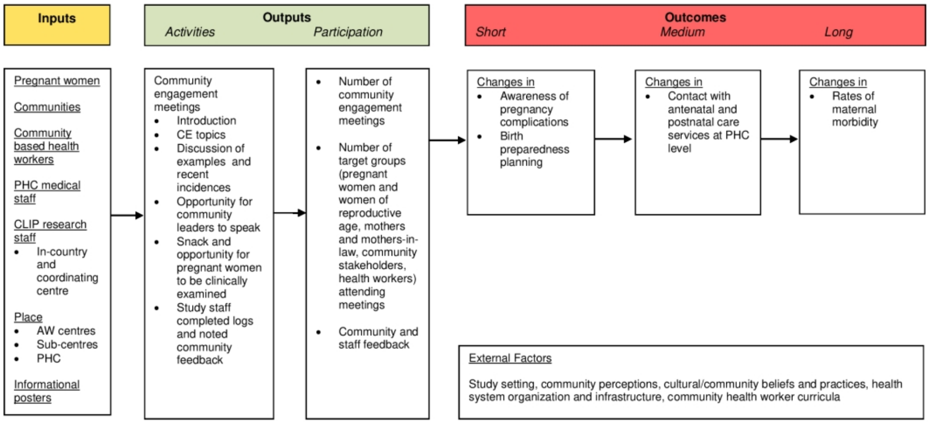

A process evaluation plan for assessing community engagement to improve maternal health in Karnataka, India

279x215mm (200 x 200 DPI)

SUPPLEMENTARY FILES

**Table S1:** Consort 2020 Checklist	Page 2
**Table S2:** Summary of pre-eclampsia knowledge by cluster [n (%)]	Page 6
**Table S3:** Summary of birth preparedness by cluster [n (%)]	Page 8
**Figure S1:** Informational poster on danger signs of hypertensive disorders of pregnancy	Page 9

For peer review only

Table S1 Consort 2020 Checklist

CONSORT 2010 checklist of information to include when reporting a randomised trial*

Section/Topic	Item No	Checklist item	Reported on page No
**Title and abstract**			
	1a	Identification as a randomised trial in the title	Page 1
	1b	Structured summary of trial design, methods, results, and conclusions (for specific guidance see CONSORT for abstracts)	Page 2
**Introduction**			
Background and objectives	2a	Scientific background and explanation of rationale	Page 4-5
	2b	Specific objectives or hypotheses	Page 5
**Methods**			
Trial design	3a	Description of trial design (such as parallel, factorial) including allocation ratio	Page 5
	3b	Important changes to methods after trial commencement (such as eligibility criteria), with reasons	N/A*
Participants	4a	Eligibility criteria for participants	Page 5-6
	4b	Settings and locations where the data were collected	Page 5
Interventions	5	The interventions for each group with sufficient details to allow replication, including how and when they were actually administered	Page 6

For peer review only - http://bmjopen.bmj.com/site/about/guidelines.xhtml

Outcomes	6a	Completely defined pre-specified primary and secondary outcome measures, including how and when they were assessed	Page 7
	6b	Any changes to trial outcomes after the trial commenced, with reasons	N/A*
Sample size	7a	How sample size was determined	N/A*
	7b	When applicable, explanation of any interim analyses and stopping guidelines	N/A*
Randomisation:			
Sequence generation	8a	Method used to generate the random allocation sequence	Page 5
	8b	Type of randomisation; details of any restriction (such as blocking and block size)	Page 5
Allocation concealment mechanism	9	Mechanism used to implement the random allocation sequence (such as sequentially numbered containers), describing any steps taken to conceal the sequence until interventions were assigned	Page 5
Implementation	10	Who generated the random allocation sequence, who enrolled participants, and who assigned participants to interventions	Page 5
Blinding	11a	If done, who was blinded after assignment to interventions (for example, participants, care providers, those assessing outcomes) and how	N/A*
	11b	If relevant, description of the similarity of interventions	N/A*
Statistical methods	12a	Statistical methods used to compare groups for primary and secondary outcomes	Page 7-8
	12b	Methods for additional analyses, such as subgroup analyses and adjusted analyses	
**Results**			
Participant flow (a diagram is	13a	For each group, the numbers of participants who were randomly assigned, received intended treatment, and were analysed for the primary outcome	Page 5*

For peer review only - http://bmjopen.bmj.com/site/about/guidelines.xhtml

strongly recommended)	13b	For each group, losses and exclusions after randomisation, together with reasons	N/A*
Recruitment	14a	Dates defining the periods of recruitment and follow-up	Page 6
	14b	Why the trial ended or was stopped	Page 6
Baseline data	15	A table showing baseline demographic and clinical characteristics for each group	Page 5*
Numbers analysed	16	For each group, number of participants (denominator) included in each analysis and whether the analysis was by original assigned groups	N/A*
Outcomes and estimation	17a	For each primary and secondary outcome, results for each group, and the estimated effect size and its precision (such as 95% confidence interval)	Page 9-11
	17b	For binary outcomes, presentation of both absolute and relative effect sizes is recommended	Page 5*
Ancillary analyses	18	Results of any other analyses performed, including subgroup analyses and adjusted analyses, distinguishing pre-specified from exploratory	N/A*
Harms	19	All important harms or unintended effects in each group (for specific guidance see CONSORT for harms)	N/A*
**Discussion**			
Limitations	20	Trial limitations, addressing sources of potential bias, imprecision, and, if relevant, multiplicity of analyses	Page 14
Generalisability	21	Generalisability (external validity, applicability) of the trial findings	Page 13-14
Interpretation	22	Interpretation consistent with results, balancing benefits and harms, and considering other relevant evidence	Page 13-14

For peer review only - http://bmjopen.bmj.com/site/about/guidelines.xhtml

**Other information**			
Registration	23	Registration number and name of trial registry	Page 5
Protocol	24	Where the full trial protocol can be accessed, if available	N/A*
Funding	25	Sources of funding and other support (such as supply of drugs), role of funders	Page 15

*For more details on the methodology, implementation and results of the full CLIP Trial in India, please see: Bellad MB, Goudar SS, Mallapur AA, Sharma S, Bone J, Charantimath US, et al. Community level interventions for pre-eclampsia (CLIP) in India: A cluster randomised controlled trial. Pregnancy Hypertension. 2020 Jul 1;21:166–75.

For peer review only

Table S2: Summary of pre-eclampsia knowledge by cluster [n (%)]

	Intervention Clusters						Control Clusters						Total
	Hosur	Kanagala	Sangolli	Pattadaka	Amingad	Sutugundar	Aihole	Galagali	Katageri	Belavadi	Chachadi	Ammangi	
Composite	122 (7.5)	17 (1.6)	62 (5.9)	4 (0.3)	221 (13.2)	13 (1.3)	6 (0.6)	94 (6.9)	12 (1.1)	3 (0.2)	26 (2.0)	10 (1.0)	590 (3.4)
Can name at least 4 symptoms	122 (7.0)	17 (1.6)	62 (5.9)	4 (0.3)	223 (13.3)	13 (1.3)	6 (0.6)	96 (7.0)	12 (1.1)	3 (0.2)	26 (2.0)	10 (1.0)	594 (4.0)
Aware women can have abnormal bleeding in pregnancy	656 (40.3)	325 (30.5)	406 (38.8)	296 (21.3)	171 (10.2)	127 (12.3)	36 (3.9)	1034 (75.4)	268 (24.1)	494 (39.0)	442 (34.2)	94 (9.5)	4349 (29.4)
Aware women can have seizure in pregnancy	624 (38.3)	316 (29.7)	345 (32.9)	276 (19.8)	145 (8.6)	108 (10.5)	19 (2.1)	810 (59.1)	260 (23.4)	334 (26.4)	236 (18.3)	32 (3.2)	3505 (23.7)
Aware women can have high blood pressure in pregnancy	817 (50.1)	584 (54.8)	553 (52.8)	264 (19.0)	732 (43.6)	193 (18.8)	38 (4.1)	1000 (72.9)	339 (30.5)	718 (56.7)	512 (39.7)	466 (47.2)	6216 (42.1)

For peer review only - http://bmjopen.bmj.com/site/about/guidelines.xhtml

Aware high blood pressure in pregnancy can be life threatening	793 (48.7)	584 (54.8)	552 (52.7)	232 (16.7)	736 (43.8)	189 (18.4)	37 (4.0)	968 (70.6)	232 (20.9)	626 (49.5)	511 (39.6)	466 (47.2)	5926 (40.1)

For peer review only

Table S3: Summary of birth preparedness by cluster [n (%)]

	Intervention Clusters						Control Clusters						Total
	**Hosur**	**Kanagala**	**Sangolli**	**Pattadakal lu**	**Amingad**	**Sutugunda r**	**Aihole**	**Galagali**	**Katageri**	**Belavadi**	**Chachadi**	**Ammangi**	
Composite	1556 (95.5)	1013 (95.1)	1044 (99.7)	869 (62.5)	466 (27.8)	639 (62.1)	702 (76.5)	878 (64.0)	827 (74.4)	1241 (98.0)	1289 (99.9)	932 (94.4)	11456 (77.5)
Arranged transport	1548 (95.0)	995 (93.4)	1044 (99.7)	1160 (83.5)	475 (28.3)	887 (86.2%)	720 (78.4)	1065 (77.7)	859 (77.3)	1262 (99.7)	1291 (100.0)	848 (85.9)	12154 (82.2)
Has permission for emergency care	1626 (99.8)	1063 (99.8)	1045 (99.8)	1372 (98.7)	1634 (97.3)	855 (83.0)	909 (99.0)	1362 (99.3)	1015 (91.4)	1264 (99.8)	1291 (100.0)	984 (99.7)	14420 (97.5)
Has money saved for emergency	1324 (81.2)	1008 (94.6)	907 (86.6)	127 (9.1)	385 (22.9)	118 (11.4)	481 (52.4)	327 (23.9)	364 (32.8)	723 (57.1)	1168 (90.5)	977 (99.0)	7909 (53.5)
Identified facility for delivery	1572 (96.5)	1058 (99.3)	1002 (95.7)	1011 (72.7)	1377 (82.0)	836 (81.2)	745 (81.2)	997 (72.7)	874 (78.7)	1210 (95.6)	1266 (98.1)	903 (91.5)	12851 (86.9)

For peer review only

Figure S1: Informational poster on danger signs of hypertensive disorders of pregnancy

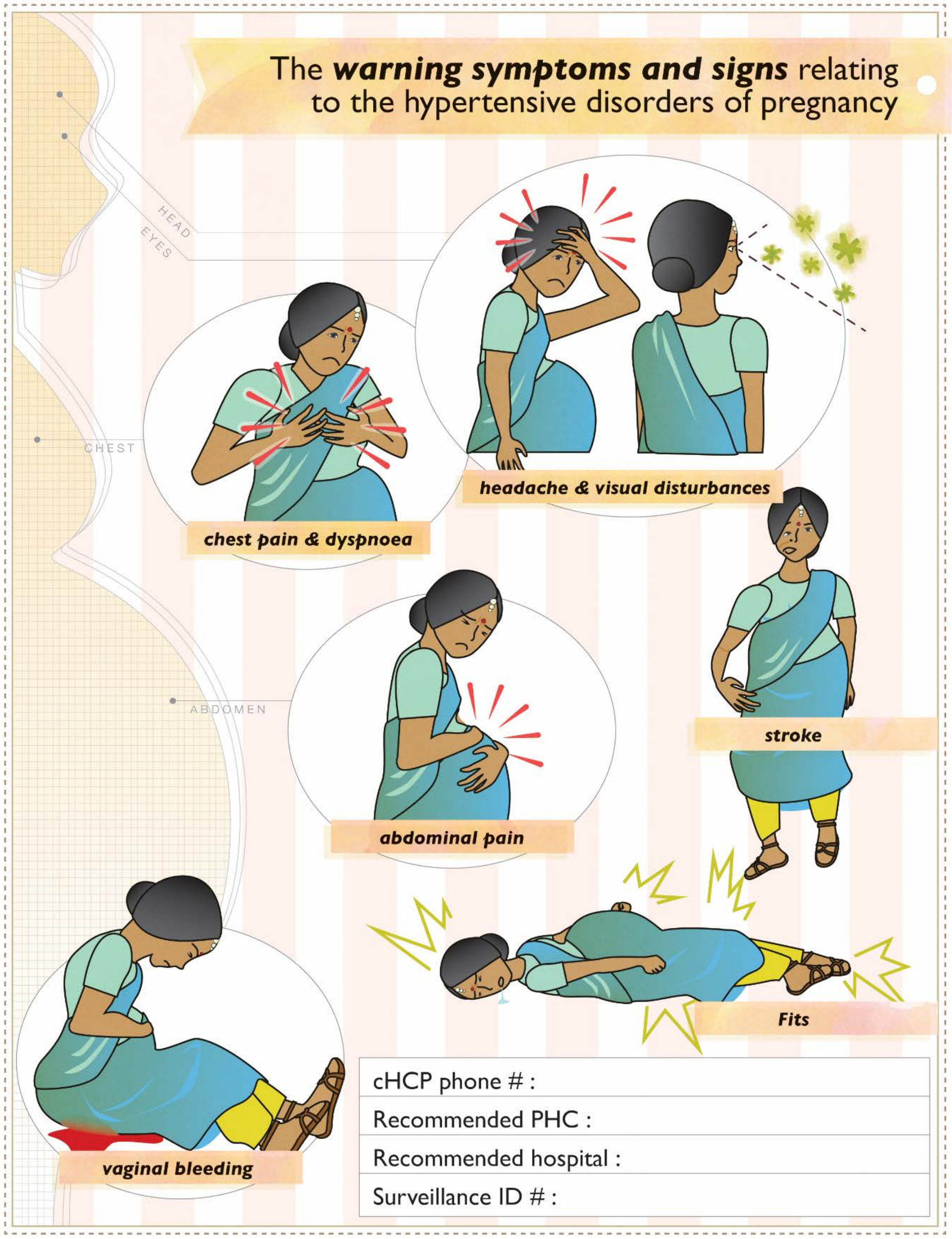

For peer review only - http://bmjopen.bmj.com/site/about/guidelines.xhtml

CONSORT 2010 checklist of information to include when reporting a randomised trial*

Section/Topic	Item No	Checklist item	Reported on page No
**Title and abstract**			
	1a	Identification as a randomised trial in the title	Page 1
	1b	Structured summary of trial design, methods, results, and conclusions (for specific guidance see CONSORT for abstracts)	Page 2
**Introduction**			
Background and objectives	2a	Scientific background and explanation of rationale	Page 4
	2b	Specific objectives or hypotheses	Page 4
**Methods**			
Trial design	3a	Description of trial design (such as parallel, factorial) including allocation ratio	Page 4-5
	3b	Important changes to methods after trial commencement (such as eligibility criteria), with reasons	N/A*
Participants	4a	Eligibility criteria for participants	Page 5
	4b	Settings and locations where the data were collected	Page 5
Interventions	5	The interventions for each group with sufficient details to allow replication, including how and when they were actually administered	Page 5-6
Outcomes	6a	Completely defined pre-specified primary and secondary outcome measures, including how and when they were assessed	Page 7
	6b	Any changes to trial outcomes after the trial commenced, with reasons	N/A*

For peer review only

Sample size	7a	How sample size was determined	N/A*
	7b	When applicable, explanation of any interim analyses and stopping guidelines	N/A*
Randomisation:			
Sequence generation	8a	Method used to generate the random allocation sequence	Page 5
	8b	Type of randomisation; details of any restriction (such as blocking and block size)	Page 5
Allocation concealment mechanism	9	Mechanism used to implement the random allocation sequence (such as sequentially numbered containers), describing any steps taken to conceal the sequence until interventions were assigned	N/A*
Implementation	10	Who generated the random allocation sequence, who enrolled participants, and who assigned participants to interventions	N/A*
Blinding	11a	If done, who was blinded after assignment to interventions (for example, participants, care providers, those assessing outcomes) and how	N/A*
	11b	If relevant, description of the similarity of interventions	N/A*
Statistical methods	12a	Statistical methods used to compare groups for primary and secondary outcomes	Page 6
	12b	Methods for additional analyses, such as subgroup analyses and adjusted analyses	Page 6
**Results**			
Participant flow (a diagram is strongly recommended)	13a	For each group, the numbers of participants who were randomly assigned, received intended treatment, and were analysed for the primary outcome	N/A*
	13b	For each group, losses and exclusions after randomisation, together with reasons	N/A*
Recruitment	14a	Dates defining the periods of recruitment and follow-up	Page 5

For peer review only

	14b	Why the trial ended or was stopped	N/A*
Baseline data	15	A table showing baseline demographic and clinical characteristics for each group	Page 7-8, table 1
Numbers analysed	16	For each group, number of participants (denominator) included in each analysis and whether the analysis was by original assigned groups	Page 8-9, tables 2 and 3
Outcomes and estimation	17a	For each primary and secondary outcome, results for each group, and the estimated effect size and its precision (such as 95% confidence interval)	Page 8-9, tables 2 and 3
	17b	For binary outcomes, presentation of both absolute and relative effect sizes is recommended	N/A*
Ancillary analyses	18	Results of any other analyses performed, including subgroup analyses and adjusted analyses, distinguishing pre-specified from exploratory	N/A*
Harms	19	All important harms or unintended effects in each group (for specific guidance see CONSORT for harms)	N/A*
**Discussion**			
Limitations	20	Trial limitations, addressing sources of potential bias, imprecision, and, if relevant, multiplicity of analyses	Page 13
Generalisability	21	Generalisability (external validity, applicability) of the trial findings	Page 13
Interpretation	22	Interpretation consistent with results, balancing benefits and harms, and considering other relevant evidence	Page 12-13
**Other information**			
Registration	23	Registration number and name of trial registry	Page 4
Protocol	24	Where the full trial protocol can be accessed, if available	N/A*

For peer review only

Funding	25	Sources of funding and other support (such as supply of drugs), role of funders	Page 14

*For more details on the methodology, implementation and results of the full CLIP Trial in India, please see: Bellad MB, Goudar SS, Mallapur AA, Sharma S, Bone J, Charantimath US, Katageri GM, Ramadurg UY, Mark Ansermino J, Derman RJ, Dunsmuir DT, Honnungar NV, Karadiguddi C, Kavi AJ, Kodkany BS, Lee T, Li J, Nathan HL, Payne BA, Revankar AP, Shennan AH, Singer J, Tu DK, Vidler M, Wong H, Bhutta ZA, Magee LA, von Dadelszen P; CLIP India Working Group. Community level interventions for pre-eclampsia (CLIP) in India: A cluster randomised controlled trial. Pregnancy Hypertens. 2020 Jul;21:166-175. doi: 10.1016/j.preghy.2020.05.008. Epub 2020 May 19. PMID: 32554291; PMCID: PMC7471838.

For peer review only

BMJ Open

Community engagement for birth preparedness and complication readiness in the Community Level Interventions for Pre-eclampsia (CLIP) trial in India: A mixed method evaluation

Journal:	*BMJ Open*
Manuscript ID	bmjopen-2021-060593.R2
Article Type:	Original research
Date Submitted by the Author:	02-Nov-2022

Complete List of Authors:	Kavi, Avinash; KLE Academy of Higher Education and Research, Community Medicine and Women's and Children's Health Research Unit<br>Kinshella, Mai-Lei Woo; The University of British Columbia Faculty of Medicine, Department of Obstetrics and Gynaecology<br>Ramadurg, Umesh Y.; S Nijalingappa Medical College, Women's and Children's Health Research Unit<br>Charantimath, Umesh; KLE Academy of Higher Education and Research, Women's and Children's Health Research Unit<br>Katageri, Geetanjali M.; S Nijalingappa Medical College, Women's and Children's Health Research Unit<br>Karadiguddi, Chandrashekhar C.; KLE Academy of Higher Education and Research<br>Honnungar, Narayan V.; KLE Academy of Higher Education and Research<br>Bannale, Shashidhar G.; S Nijalingappa Medical College, Women's and Children's Health Research Unit<br>Mungarwadi, Geetanjali I.; KLE Academy of Higher Education and Research<br>Bone, Jeffrey; The University of British Columbia, Obstetrics and Gynecology<br>Vidler, Marianne; The University of British Columbia, Obstetrics & Gynaecology<br>Magee, Laura; King's College London School of Medical Education,<br>Mallapur, Ashalata; BVVS S Nijalingappa Medical College and HSK Hospital and Research Centre<br>Goudar, Shivaprasad S.; KLE University, Women's and Children's Health Research Unit<br>Bellad, Mrutyunjaya; KLE Academy of Higher Education and Research, Women's and Children's Health Research Unit<br>Derman, Richard ; Thomas Jefferson University<br>von Dadelszen, Peter ; King's College London Faculty of Life Sciences and Medicine, Department of Women and Children's Health<br>The CLIP India Working Group, .; The University of British Columbia Faculty of Medicine, Department of Obstetrics and Gynaecology

**Primary Subject Heading**:	Obstetrics and gynaecology
Secondary Subject Heading:	Public health

Keywords:	OBSTETRICS, Public health < INFECTIOUS DISEASES, Hypertension < CARDIOLOGY

SCHOLARONE™ Manuscripts

I, the Submitting Author has the right to grant and does grant on behalf of all authors of the Work (as defined in the below author licence), an exclusive licence and/or a non-exclusive licence for contributions from authors who are: i) UK Crown employees; ii) where BMJ has agreed a CC-BY licence shall apply, and/or iii) in accordance with the terms applicable for US Federal Government officers or employees acting as part of their official duties; on a worldwide, perpetual, irrevocable, royalty-free basis to BMJ Publishing Group Ltd ("BMJ") its licensees and where the relevant Journal is co-owned by BMJ to the co-owners of the Journal, to publish the Work in this journal and any other BMJ products and to exploit all rights, as set out in our licence.

The Submitting Author accepts and understands that any supply made under these terms is made by BMJ to the Submitting Author unless you are acting as an employee on behalf of your employer or a postgraduate student of an affiliated institution which is paying any applicable article publishing charge ("APC") for Open Access articles. Where the Submitting Author wishes to make the Work available on an Open Access basis (and intends to pay the relevant APC), the terms of reuse of such Open Access shall be governed by a Creative Commons licence – details of these licences and which Creative Commons licence will apply to this Work are set out in our licence referred to above.

Other than as permitted in any relevant BMJ Author's Self Archiving Policies, I confirm this Work has not been accepted for publication elsewhere, is not being considered for publication elsewhere and does not duplicate material already published. I confirm all authors consent to publication of this Work and authorise the granting of this licence.

For peer review only

Community engagement for birth preparedness and complication readiness in the Community Level Interventions for Pre-eclampsia (CLIP) trial in India: A mixed method evaluation

Avinash Kavi[1*]
Mai-Lei Woo Kinshella[2]
Umesh Y. Ramadurg[3]
Umesh S. Charantimath[1]
Geetanjali M. Katageri[3]
Chandrashekhar C. Karadiguddi[1]
Narayan V. Honnungar[1]
Shashidhar G. Bannale[3]
Geetanjali I. Mungarwadi[1]
Jeffrey N. Bone[2]
Marianne Vidler[2]
Laura A. Magee[4]
Ashalata A. Mallapur[3]
Shivaprasad S. Goudar[1]
Mrutyunjaya B. Bellad[1]
Richard J. Derman[5]
Peter von Dadelszen[4]
and the CLIP India Working Group

1. Women's and Children's Health Research Unit, KLE Academy of Higher Education and Research's Jawaharlal Nehru Medical College, Belagavi, Karnataka, India
2. Department of Obstetrics and Gynaecology and BC Children's Hospital Research Institute, University of British Columbia, Vancouver BC, Canada
3. Women's and Children's Health Research Unit, S. Nijalingappa Medical College, Bagalkote, Karnataka, India
4. Department of Women and Children's Health, School of Life Course Sciences, Kings College London, London, UK
5. Thomas Jefferson University, Philadelphia, USA

*Corresponding author: Avinash Kavi

Women's and Children's Health Research Unit, KLE Academy of Higher Education and Research's Jawaharlal Nehru Medical College, Belagavi, Karnataka, India

Email: dravinashkavi@gmail.com

Word count: 3932/4000

For peer review only - http://bmjopen.bmj.com/site/about/guidelines.xhtml

For peer review only

ABSTRACT

Objective: To describe the process of community engagement in northern Karnataka, India and its impact on pre-eclampsia knowledge, birth preparedness and complication readiness, pregnancy-related care seeking and maternal morbidity.

Design: This study was a secondary analysis of a cluster randomized trial of Community Level Interventions for Preeclampsia (CLIP). A total of 12 clusters based on primary health centre catchment areas were randomised to intervention or control. Community engagement was conducted in intervention clusters. Community engagement attendance was summarized according to participant group using both quantitative and qualitative assessment. Pre-eclampsia knowledge, birth preparedness, health services engagement and perinatal outcomes was evaluated within trial surveillance. Outcomes were compared between trial arms using a mixed effects logistic regression model on RStudio (RStudio Inc, Boston, United States). Community feedback notes were thematically analysed on NVivo12 (QSR International, Melbourne, Australia).

Setting: Belagavi and Bagalkote districts in rural Karnataka, India.

Participants: Pregnant women and women of reproductive age, mothers and mothers-in-law, community stakeholders and male household decision-makers, and health workers.

Results: A total of 1,379 community engagement meetings were conducted with 39,362 participants between November 2014 and October 2016. Community engagement activities may have had an effect on modifying community attitudes towards hypertension in pregnancy and its complications. However, rates of pre-eclampsia knowledge, birth preparedness, health services engagement and maternal morbidities among individual pregnant women were not significantly impacted by community engagement activities in their area.

Conclusion: Evaluation of our community engagement program in India demonstrates the feasibility of reaching pregnant women alongside household decision-makers, community stakeholders and health workers. More research is needed to explore the pathways of impact between broad community mobilization to strengthen support for maternal care-seeking and clinical outcomes of individual pregnant women.

Trial registration: NCT01911494

Keywords: Community Engagement, Birth preparedness, Complication readiness, Pre-eclampsia, Community support, Barriers.

For peer review only - http://bmjopen.bmj.com/site/about/guidelines.xhtml

STRENGTHS AND LIMITATIONS OF THIS STUDY

- We systematically evaluated the effectiveness of a large-scale community engagement program conducted with the Community Level Interventions for Pre-eclampsia (CLIP) Trial in rural India using a process evaluation framework. The logic model outlines the hypothesized relationships between inputs, outputs and outcomes of interest to explore potential pathways of change.
- Data for the assessment was obtained from a prospective population-based surveillance system of women in the CLIP Trial and community engagement records including community feedback notes. Our study triangulated qualitative and quantitative datasets in a convergent mixed-methods approach.
- The community engagement program and its evaluation involved a wide range of health workers, pregnant women, household decision-makers and community leaders. Group sessions with many stakeholders allowed for a diversity of perspectives. Unfortunately, we do not have data on specific individuals.
- The limited timeframe within a clinical trial may have been too short to appropriately evaluate community-level behavioural change and more research is needed to better understand the influence of external factors on maternal health care-seeking.
- Community engagement was one of the components of a complex intervention evaluated in the CLIP Trial and interventions involving multiple factors makes the impact assessment more complicated.

For peer review only

INTRODUCTION

Over 94% of maternal deaths occur in low- and middle-income countries (LMICs), primarily in South Asia and sub-Saharan Africa, and are preventable (1,2). Maternal deaths relate primarily to delays in triage (ability of care provider and women to identify who is severely ill and requires urgent care), transport (ability to get women to appropriate care when needed) and treatment (ability to provide appropriate treatment when care accessed) (3). Increased birth preparedness and complication readiness can help mitigate against delays and ensure timely identification of the need for seeking skilled care and arrival at the appropriate facility for pregnancy complications. India has an estimated maternal mortality ratio (MMR) of 113 per 100,000 livebirths in 2016-18, with pregnancy hypertension accounting for 7% of maternal deaths (4). While India has achieved a 6.3% average annual rate of decline from 1997 to 2018, progress has varied by region (4). Though the states in southern India are often categorized as high-performing with better maternal and perinatal health indicators than much of the country, the MMR in Karnataka is double the rate in neighbouring Kerala (92 vs 42 per 100,000 live births) (4).

Birth preparedness and complication readiness (BPCR) interventions are strongly recommended by the World Health Organization (WHO) to increase skilled birth attendance and timely use of facility care for obstetric and neonatal complications (5,6). According to the WHO, BCPR involves counselling pregnant women and their families on knowledge of pregnancy danger signs and developing a BCPR plan that includes identification of health facility and transport for birth and in case of complications, identification of a birth companion and preferred birth attendant, supplies to bring to the facility, funds for any expenses, and identification of a support person to look after other children at home as well as compatible blood donors if needed (5–7). Involvement of community members are critical in the implementation of community health programs (8). In India, raising community awareness to support BPCR complements the national programme of community health workers called Accredited Social Health Activists (ASHAs), which aims to connect women and children to primary care (9). ASHAs identify pregnant women in their communities and accompany them to nearby primary health centre for registration and antenatal care (ANC) visits. However, previous research in Karnataka revealed that pregnant women rarely made health care decisions on their own, and other family members and community leaders were highly influential in decision-making (10). Bringing community members together to raise awareness of maternal health issues mobilizes wider support for maternal healthcare seeking and ASHAs' work in local communities.

Over the past few decades, community engagement (CE) has emerged as an increasingly effective strategy for harnessing community potential for health improvement (11–16). CE is defined as 'a process of working collaboratively with groups of people who are affiliated by geographic proximity, special interests, or similar situations, with respect to issues affecting their well-being' (17). It is a dynamic relational process that facilitates communication, interaction, involvement, and

For peer review only - http://bmjopen.bmj.com/site/about/guidelines.xhtml

exchange between an organization and a community for a range of social and organizational outcomes (18). This strategy has been used for health promotion, research, and policy making to address a variety of health issues (19–22). CE enables a more contextualized understanding of community members' perceptions, thereby facilitating stronger relationships. Implementation of CE enhances the delivery of the health care services in an optimal manner for mothers and newborns, which ultimately aligns with the vision of national goal.

This paper is the secondary analysis of the Community Level Interventions for Pre-eclampsia (CLIP) Trial, India; the primary paper has been published elsewhere (23).The paper describes the process of CE, one of the component of CLIP Trial, and evaluates the impact of CE on knowledge around pre-eclampsia, birth preparedness and complication readiness, pregnancy-related care seeking and rates of maternal morbidity.

METHODS

The CLIP Trial and study setting

The CLIP India Trial (Clinical Trials.gov NCT01911494) was a community-based cluster randomised controlled trial reported according to the CONSORT 2010 Statement (**Table S1 checklist**). The CLIP India Trial was conducted in rural Karnataka, India where the incidence of pregnancy hypertension is 10.3% (24). This study was conducted in 12 distinct geographic regions in Karnataka State, six in each Belagavi and Bagalkote districts (**Figure 1 map**) (**Figure 2 map**). Belagavi has a population of 4,779,661 living in 1278 villages and Bagalkote has a population of 1,889,752 living in 627 villages (25). There are an estimated 500 birth annually in each district (25). The adult female literacy rate varies in the two districts, at 70.2% in Belagavi, and 59.3% in Bagalkote, respectively (26).

The primary health centre (PHC) and its catchment area served as the unit of randomization. Clusters were chosen according to region, accessibility for surveillance, and absence of conflicting research activity. Clusters were allocated (TL, statistician) using a restricted randomisation algorithm, balancing region and population size to ensure equivalence between intervention and control clusters. Stratified randomisation was undertaken to allocate six clusters each to the intervention and control groups, using population size as a single stratification factor. Both intervention and control clusters were similar in terms of population (11.6 per hectare in intervention clusters vs 13.3 per hectare in control clusters) and community health workers (17 vs 18 ASHAs per cluster) density (23). Among the pregnant women enrolled in the CLIP Trial (7,839 in intervention clusters, 6,944 in the control clusters), maternal age was on average 22-23 years old, 56-58% of women had 8 or more years of schooling, and populations were over 90% Hindu without significant difference between intervention and control clusters (23).

For peer review only - http://bmjopen.bmj.com/site/about/guidelines.xhtml

The overall CLIP Trial focused on implementing community-level evidence-based care to reduce all cause maternal and perinatal mortality and major morbidity by supporting early identification and prompt referrals for pregnancy complications. The intervention included community engagement alongside strengthening clinical capacities of ASHAs to assess blood pressure and usage of PIERS-On-the-Move (POM). POM is a mobile-based application decision aid for community health workers to identify women at risk of adverse outcomes from pregnancy hypertension and guide management (27,28). More detail on the overall CLIP India Trial, participant characteristics, and reporting on the primary outcomes have been published elsewhere (23).

Process of facilitating community engagement in the CLIP Trial

The CE component was carried out in intervention clusters over the entire trial period (November 2014 - October 2016). The CE activities aimed at creating culturally and contextually appropriate discussion to improve maternal health awareness and action around the prevention of maternal morbidity and mortality. CE aimed to improve birth preparedness, nutrition, and promote appropriate ANC and healthcare seeking. Topics included warning signs of pregnancy complications, permission to seek care, identifying a skilled birth attendant, facility for delivery, and mode of transport as well as saving up funds for transport and treatment. Discussions also included adverse pregnancy outcomes and success stories in the community, overcoming local barriers to accessing maternal health care and the interventions in the CLIP Trial.

Community engagement meetings were primarily held at the anganwadi (AW) centres in the village, which offer basic health services. These were locations where pregnant women and children routinely access ANC, immunizations and other maternal and child health care services. Meetings were also held at sub-centres, PHCs, temples, public halls, schools or other appropriate nearby locations for large monthly sessions.

The activities convened multiple levels of the local health care system. This included workers (AWW) at the AW centres, ASHAs who worked in the villages, Auxiliary Nurse Midwives (ANMs) who worked at sub-centres, Lady Health Visitors (LHV) who supervised the ANMs, and medical officers who coordinated the PHC. In addition, ASHA supervisors and Registry Administrators (RAs) appointed as a part of the trial also attended meetings.

Pregnant women and married women of reproductive age groups were primary beneficiaries while their family members and other household decision-makers were secondary targets. Household decision-makers were often in charge of women's decisions to seek care and community leaders provided advice and financial assistance for healthcare seeking at times. Community leaders included elected representatives and teachers.

The CE activity schedule was prepared at the beginning of each month by the local study team and shared to the respective community stakeholders. Meetings lasted between 30 and 150 minutes. These meetings were facilitated by the study research

For peer review only - http://bmjopen.bmj.com/site/about/guidelines.xhtml

officers with support from the medical officers at each PHC. Sessions began with a short formal introduction and description of the purpose, then discussion of CE topics prompted by informational posters developed for the project (for example, see **Figure S1**). Participants were encouraged to actively participate, share personal experiences and discuss case examples. There were no monetary incentives for participants to attend the meeting. Participants were provided with nutritional snacks and fruit.

Data collection and analysis

For the purpose of assessing implementation, study staff and supervisors completed CE logs after each meeting. This included a quantitative dataset on the number of participants attending each meeting and target groups reached, as well as a qualitative dataset comprised of community feedback. For assessing the potential impact of CE activities on health outcomes, quantitative data was collected from the prospective population-based CLIP Trial Surveillance system of pregnant women enrolled in the intervention and control groups of the study (23,29). Data collected from the CLIP Trial Surveillance included demographic factors, birth preparedness, care-seeking, obstetric information, pre-eclampsia knowledge, perinatal outcomes and treatment information. Data forms were reviewed for completeness and entered weekly into the local database. De-identified and encrypted data were transferred to the central server for analyses.

We employed a convergent mixed-methods approach, which involves quantitative and qualitative data collection separately and integrating them at the point of interpretation to highlight the areas where the two datasets converge (30). **Figure 3** illustrates the strategy for assessing CE in the CLIP India Trial, based on the process evaluation framework for the CLIP Pilot Trial in Nigeria (31).

- **CE attendance** was summarized according to participant group: pregnant women and other women of reproductive age, the pregnant women's mothers and mothers-in-law, community stakeholders (community leaders, male and female decision-makers), health workers (AWW, ANM, ASHA, ASHA supervisor, LHV, medical officers, nurses, RA) and others (family members, neighbours, friends, community members).
- **Pre-eclampsia knowledge** was based on the clinical symptoms that predicted adverse maternal and perinatal outcomes in women with pre-eclampsia and included evaluating awareness of: 1) high blood pressure during pregnancy 2) abnormal bleeding in pregnancy , 3) seizures in pregnancy, 4) that pregnancy hypertension can be life threatening (32). The composite pre-eclampsia knowledge score included recalling at least one of the above conditions and at least four symptoms of high blood pressure in pregnancy.
- **Birth preparedness** evaluated a pregnant woman's plans for: 1) transport in case of emergencies, 2) permission to seek emergency care, 3) money saved for an emergency, 4) identified health facility for delivery, and 5) at least two of the components for the BCPR composite score.

For peer review only - http://bmjopen.bmj.com/site/about/guidelines.xhtml

Pre-eclampsia knowledge, birth preparedness, health services engagement and perinatal outcomes indicators were summarized between arms with counts and frequencies for categorical variables and medians and interquartile range [IQR] for continuous variables. For health service questions, we restricted the denominators to women who delivered by trial end as these indicators are more likely to occur later in pregnancy. As pre-eclampsia knowledge and birth preparedness were asked throughout pregnancy and all women received community engagement, we included all women in assessing these rates. Outcomes were further compared between trial arms using a mixed effects logistic regression model (to account for clustering) with a logit link. Models were adjusted for age, maternal education, husband education, gestational age at booking, and parity and results are presented as odds ratios and 95% confidence intervals. Quantitative data were analysed using RStudio (RStudio Inc, Boston, United States).

Community feedback recorded by study staff on CE logs were qualitatively analysed using a descriptive, exploratory approach to understand how communities received CE activities and supported interpretation of quantitative findings (**Table S2 checklist)**. Feedback was recorded as open-text and included some individual comments and some group feedback given during the CE sessions. Community feedback notes were imported to NVivo12 software (QSR International, Melbourne, Australia) for thematic analysis (33). Coding was conducted by qualitative experts (MWK, MV) in close collaboration with a community maternal and child health clinician-scientist from India (AV). Triangulation of qualitative and quantitative data enhanced trustworthiness of findings.

Research ethics approval

The project received ethics committee approvals from KLE Academy of Higher Education and Research Deemed-to-be-University (MDC/IECHSR/2011-12/A-4; ICMR 5/7/859/12-RHN) and the University of British Columbia (UBC, H12-03497).

Patient and public involvement

Neither patients nor public were involved in the design or conduct of the study.

RESULTS

Community engagement attendance

A total of 1,379 CE meetings were conducted the 6 intervention clusters. Among them 586 sessions were conducted in Belagavi district and 773 sessions at Bagalkote district. The median number of sessions per cluster was 204 [IQR 186 – 281]. **Table 1** describes the activities across clusters and the participants reached. As long as pregnant women remained in the same village, they were encouraged to attend all sessions. The other participants, such as stakeholders and decision-makers, were usually different for each session. Pregnant women were most represented at the meetings, followed by mothers-

For peer review only - http://bmjopen.bmj.com/site/about/guidelines.xhtml

in-law who often accompanied them. Husbands and fathers-in-law were least represented in the meetings as they often had competing priorities; however, overall sessions were well attended by target groups.

Table 1: Participants reached in CE activities in the intervention clusters

Target groups	Number of participants (% of total)	Number of sessions (% of total)
Pregnant women and other women of reproductive age	22,149 (56.3%)	1,379 (100.0%)
Mothers and mothers-in-law	4,643 (11.8%)	1,140 (82.7%)
Community stakeholders	5,685 (14.4%)	1,147 (83.2%)
Health workers	4,752 (12.1%)	1,376 (99.8%)
Others	2,113 (5.4%)	N/A
Total	39,342	1,379

Impact on clinical outcomes

Tables 2 and **3** present summaries of pre-eclampsia knowledge and birth preparedness among pregnant women in the CLIP Trial. The rates for both (composite and components) were not significantly different between arms. Only a minority of participants from both intervention and control (2-6%) could name at least four symptoms of high blood pressure in pregnancy or had overall pre-eclampsia knowledge. In contrast, there were higher levels of birth preparedness among women in both intervention and control with a majority of women reporting that they had arranged transport, has permission for emergency care, and identified a health facility for delivery. Only saving funds for an obstetric emergency was lower, with about half of women declaring that they had funds saved up. Confidence intervals of odd ratios are wide, reflecting high heterogeneity between study clusters [see **Tables S3 and S4**].

Table 2: Summary of pre-eclampsia knowledge by arm

	**Intervention** (n= 7,839)	**Control** (n= 6,944)	**Total*** (n=14,783)	**Adjusted odds ratio (95%CI);**	**p-value†**
Women can have abnormal bleeding in pregnancy	1,981 (25.3%)	2,368 (34.1%)	4,349 (29.4%)	0.88 (0.25, 3.18);	0.85
Women can have seizure in pregnancy	1,814 (23.1%)	1,691 (24.4%)	3,505 (23.7%)	1.49 (0.40, 5.62);	0.55
Women can have high blood pressure in pregnancy	3,143 (40.1%)	3,073 (44.3%)	6,216 (42.1%)	1.02 (0.32, 3.25);	0.97

For peer review only - http://bmjopen.bmj.com/site/about/guidelines.xhtml

High blood pressure in pregnancy can be life threatening	3,086 (39.4%)	2,840 (40.9%)	5,926 (40.1%)	0.86 (0.17, 4.21);	0.85
Can name at least four symptoms	441 (5.6%)	153 (2.2%)	594 (4.0%)	2.35 (0.60, 9.12);	0.22
Composite††	439 (5.6%)	151 (2.2%)	590 (4.0%)	2.35 (0.61, 9.10);	0.22

* *All pregnancies enrolled in the CLIP India Trial*

† *Estimated by mixed effects logistic regression adjusted for: maternal age, maternal education, husband education, gestational age at enrolment, parity, and cluster (random effect).*

††*Recall at least one condition and at least four symptoms of high blood pressure*

Table 3: Summary of birth preparedness by arm

	**Intervention** (n= 7,839)	**Control** (n= 6,944)	**Total** (n=14,783)	**Adjusted odds ratio (95%CI);**	**p-value***
Arranged transport	6,109 (77.9%)	6,045 (87.1%)	12,154 (82.2%)	0.40 (0.03, 6.07);	0.51
Has permission for emergency care	7,595 (96.9%)	6,825 (98.3%)	14,420 (97.5%)	0.48 (0.05, 4.31);	0.51
Has money saved for emergency	3,869 (49.4%)	4,040 (58.2%)	7,909 (53.5%)	0.46 (0.05, 4.19);	0.49
Identified facility for delivery	6,856 (87.5%)	5,995 (86.3%)	12,851 (86.9%)	1.50 (0.36, 6.27);	0.58
Composite†	5,587 (71.3%)	5,869 (84.5%)	11,456 (77.5%)	0.50 (0.05, 5.59);	0.58

**All pregnancies enrolled in the CLIP India Trial*

† *Estimated by mixed effects logistic regression adjusted for: maternal age, maternal education, husband education, gestational age at enrolment, parity, and cluster (random effect).*

††*Having at least two of the components of birth preparedness ready.*

Table 4 illustrates that health services engagement by pregnant women in the CLIP Trial were similar between arms. Almost all women had at least one ANC visit and three out of four women had at least four visits. Approximately one in ten women visited the PHC for reason other than routine care and about 3% of women were admitted to a health facility for reasons other than delivery, staying a median of three days. One in twenty women experienced a maternal morbidity and there was no significant difference between arms (OR 0.95, 95%CI: 0.61-1.50, p= 0.84).

Table 4: Health services engagement and maternal morbidity by arm

For peer review only - http://bmjopen.bmj.com/site/about/guidelines.xhtml

For peer review only

	**Intervention** (N=6,908)	**Control** (N=6,109)	**Total*** (N=13,017)	**Adjusted odds ratio (95%CI);**	**p-value†**
Women with at least one ANC visit	6,891 (99.8%)	6,068 (99.3%)	12,959 (99.6%)	Cannot be computed	-
Women with 4 or more ANC visits	5,140 (74.4%)	4,745 (77.7%)	9,885 (75.9%)	0.86 (0.51, 1.44);	0.57
Visited PHC for other than routine care	823 (11.9%)	739 (12.1%)	1562 (12.0%)	1.92 (0.43, 8.55);	0.39
Visited higher level facility for other than routine care	1,069 (15.5%)	780 (12.8%)	1849 (14.2%)	1.59 (0.58, 4.38);	0.37
Number of women admitted to health facility for reasons other than delivery	225 (3.3%)	170 (2.8%)	395 (3.0%)	1.28 (0.08, 19.3);	0.86
Median days of admission [IQR]	3.0 [2.0; 5.0]	3.0 [2.0; 5.0]	3.0 [2.0; 5.0]	-	-
Maternal morbidity††	371/7,839 (4.7%)	325/6,944 (4.7%)	696/14,783 (4.7%)	0.95 (0.61, 1.50);	0.84

**Pregnancies enrolled in the CLIP India Trial with postpartum follow-up*

† Estimated by mixed effects logistic regression adjusted for: maternal age, maternal education, husband education, gestational age at enrolment, parity, and cluster (random effect).

††Data taken from CLIP India primary trial paper and includes different adjustment factors (34)

Community feedback on community engagement activities

<u>Engaging family members</u>

Staff noted that communities appreciated engaging household decision-makers and community stakeholders alongside pregnant women and health workers. Household decision-makers may not have been aware of pre-eclampsia and other maternal health risks. Their inclusion in the discussion supported care for pregnant women and increased value in attending ANC.

> “The people appreciated these community engagement meetings. They said that they learnt many things about the care of the pregnant women, which they were not aware of earlier. The pregnant women became more aware about their health and their parents also started giving more support and care to her.”

> “Earlier, it was difficult for the woman to take permission from the decision makers in the family to seek antenatal care. But after conducting the community

For peer review only - http://bmjopen.bmj.com/site/about/guidelines.xhtml

engagement meetings, the views of the decision-makers were changed so that the seeking of permission for antenatal care became easy."

Mobilizing community support for maternal health

CE feedback indicated increased community support for BPCR. There were examples of community leaders advocating for appropriate maternal health care seeking with household decision-makers as well as pushing for change to support local health systems.

> "The views of stakeholders also changed so much that the arrangement of transport, financial support, counselling the decision-makers became effective. For example, in the initial period of the study, there was one incident where the husband, the male decision-maker of the family, of the woman with eclampsia refused to take her to the hospital but after counselling by the community leaders and his family doctor, he agreed to take her to the higher care hospital."

> "The community leaders recognized the significant role played by the ASHAs and the PHC personnel in promoting the health of the pregnant women. The PHC did not have an ambulance of its own and the community leaders saw to it that a new ambulance was made available at the PHC. They also built a room above the PHC building as a meeting place for the AHSAs to continue the work actively."

External factors and barriers

Barriers of poverty, health infrastructure gaps and poor quality of care at facilities were frequently highlighted in CE discussions.

> "People accepted all the topics. They only found the issue of arranging money to be a problem because they had meagre earnings."

Additionally, the cost of treatment at private hospitals was often brought up in discussions in conjunction with the lack of services at government facilities. Some participants pointed out that there was no obstetric specialist at a nearby secondary level hospital for a period of time. Staff reflected that community members sometimes highlighted the need to strengthen care at facilities in addition to raising community awareness.

> "There were some suggestions from the community about the improvement of services at the local government health centres."

At referral facilities, participants reported that they often had to wait a long time before they would be provided services. They also reported that rural, uneducated people may encounter stigma and discrimination when seeking care at urban tertiary facilities.

For peer review only - http://bmjopen.bmj.com/site/about/guidelines.xhtml

"The participants wanted to know how to get immediate admission and care at higher and bigger tertiary care hospitals."

"They also demanded about early admission and prompt service at the tertiary care hospitals as there are some uneducated persons in the community who face some problems in getting the services in bigger hospitals."

DISCUSSION

Summary of findings and comparisons with the literature

Our evaluation of the CLIP CE program in India demonstrates the feasibility of reaching pregnant women alongside household decision-makers, community stakeholders and health workers. This included 1,379 CE sessions reaching out to nearly 40,000 stakeholders in the community. Engaging household decision-makers and community leaders together with pregnant women to discuss pregnancy care may have helped to shift local opinions on the value of ANC to monitor for pregnancy complications and mitigate risks. However, rates of pre-eclampsia knowledge, birth preparedness, health services engagement and maternal morbidities remained similar across arms in the CLIP Trial. According to our evaluation framework (**Figure 3**), our study found no evidence that the outputs were associated with our expected outcomes.

Although research findings related to the effect of community participation to improve access to maternal health care services have been mixed (35), a previous study in Uttar Pradesh in Northern India found significant effects of community health worker home visits and monthly community meetings on improved self-recognition of problems faced during pregnancy, birth preparedness components and knowledge of pregnancy danger signs (36). Differing outcomes between our findings and the study in Uttar Pradesh highlight two issues: first, the importance of context, and secondly, challenges in measuring impacts of community mobilization. Uttar Pradesh has much higher maternal mortality rates than Karnataka (37), suggesting that CE may have a stronger effect where there is lower baseline access to maternal health services. Secondly, in contrast to targeted community meetings with traditional maternal-care providers and birth attendants, our CE strategy was focused on broader community mobilization and strengthening support for maternal care-seeking among community stakeholders, which has previously been discussed in the literature as challenging to measure (8,38).

Community engagement in context and potential implications for policy and practice

CE involved shifting opinions on maternal health and cultures of care, behavioural change and mobilization and can take more time than the limited timeframe within a clinical trial. Logic models can help to understand the pathways of change, however, some of our expected short-term effects in birth preparedness via community mobilization may take more time than measured during the trial. A systematic review

For peer review only - http://bmjopen.bmj.com/site/about/guidelines.xhtml

found increased knowledge of birth preparedness and complication readiness as a result of interventions but increased knowledge did not always correspond to increased utilization of maternal health services (39). While the CE program was successful in reaching a wide audience of participants and community feedback suggested increased support for maternal health care-seeking, these may be upstream indicators or insufficient to change clinical outcomes.

A systematic review on the effects of community participation on improving uptake of skilled maternal and newborn care highlighted the importance of qualitative research to understand unforeseen impacts and socio-political factors (35). While quantitative results did not reveal an effect in our study, staff qualitatively noted that effects were noticeable in the opinions of community leaders and household decision-makers in their support for pregnancy care. Additionally, the discrepancy between the quantitative and qualitative results may also highlight the influence of external factors where community members gained appreciation of maternal health issues but were limited in their capacity to access care. Poverty and quality of care available at local health facilities emerged as significant barriers shared in community feedback, which is in line with previous research on the determinants of maternal health care services utilization in the area (10).

Nevertheless, qualitative findings that suggested improved awareness and attitudes towards pregnancy hypertension and its complications among community leaders and household decision-makers is promising. Previous research in the area found lack of knowledge around pre-eclampsia and eclampsia (40). Hypertension specifically as a pregnancy condition was unknown and there was no specific terminology in the local language (Kannada) for pre-eclampsia. CE has significant repercussions for the implementation of health care interventions and policy making.

Strengths and limitations

Strengths of our CE program and assessment include its systematic evaluation using a logic model framework and involvement of a wide range of health workers, pregnant women, household decision-makers and community leaders. Our study was also able to triangulate qualitative and quantitative datasets in a convergent mixed-methods approach.

However, as a secondary analysis, our evaluation of the impact of CE activities was limited. The effects of CE on community leaders and household decision-makers were not adequately captured in our evaluation framework that focused on measuring effects with only the pregnant women enrolled in the trial. Though qualitative findings revealed various themes and quotes were collected from CE sessions, they reflected comments from the group and unfortunately, we do not have individual-level CE feedback data. Additionally, the possibility of contamination cannot be ruled out as the clusters belonged to the same district. The health workers from the intervention and control clusters met at district-level meetings and may have discussed the intervention. This is a general limitation for cluster randomized settings. Furthermore, since CE was

For peer review only - http://bmjopen.bmj.com/site/about/guidelines.xhtml

one of the components of a complex intervention, the effects assessed do not reflect CE alone. Intervention of multiple factors makes the impact assessment much more complicated.

CONCLUSION

Our study found that CE activities may have had an effect on modifying community attitudes towards hypertension in pregnancy and its complications but did not find an impact on individual women's pre-eclampsia knowledge, birth preparedness and care-seeking indicators. Contemplation of specific individual factors and community capabilities in engaging in the health seeking behaviours would contribute to the vision of supporting people-centred methodologies to deliver health promotion. Through the complex social interactions inherent in the current CE activities, appraisals over time will evaluate the effectiveness and sustainability of these interventions.

ACKNOWLEDGEMENTS

We would like to thank the women and their families who contributed data to this trial, in addition to the ASHA and ANMs who deliver care to the most vulnerable populations. Also, we would like to acknowledge the key contributions of the following DSMB members: Romano Nkumbwa Byaruhanga, Brian Darlow, Eileen Hutton (Chair), and Lehana Thabane.

We would also like to acknowledge the CLIP India Working Group members: Shashidhar G Bannale, Keval S Chougala, Vaibhav B Dhamanekar, Anjali M Joshi, Namdev A Kamble, Gudadayya S Kengapur, Uday S Kudachi, Sphoorthi S Mastiholi, Geetanjali I Mungarwadi, Esperança Sevene, Khátia Munguambe, Charfudin Sacoor, Eusébio Macete, Helena Boene, Felizarda Amose, Orvalho Augusto, Cassimo Bique, Ana Ilda Biz, Rogério Chiaú, Silvestre Cutana, Paulo Filimone, Emília Gonçálves, Marta Macamo, Salésio Macuacua, Sónia Maculuve, Ernesto Mandlate, Analisa Matavele, Sibone Mocumbi, Dulce Mulungo, Zefanias Nhamirre, Ariel Nhancolo, Cláudio Nkumbula, Vivalde Nobela, Rosa Pires, Corsino Tchavana, Anifa Vala, Faustino Vilanculo, Rahat N Qureshi, Sana Sheikh, Zahra Hoodbhoy, Imran Ahmed, Amjad Hussain, Javed Memon, Farrukh Raza, Olalekan O Adetoro, John O Sotunsa, Sharla K Drebit, Chirag Kariya, Mansun Lui, Diane Sawchuck, Ugochi V Ukah, Mai-Lei Woo Kinshella, Shafik Dharamsi, Guy A Dumont, Tabassum Firoz, Ana Pilar Betrán, Susheela M Engelbrecht, Veronique Filippi, William A Grobman, Marian Knight, Ana Langer, Simon A Lewin, Gwyneth Lewis, Craig Mitton, Nadine Schuurman, James G Thornton, France Donnay, Romano N Byaruhanga, Brian Darlow, Eileen Hutton, Mario Merialdi, Lehana Thabane, Kelly Pickerill.

DECLARATIONS OF INTEREST

The authors declare that they have no competing interests.

ROLE OF THE FUNDING SOURCE

This trial was funded by the University of British Columbia, a grantee of the Bill &

For peer review only - http://bmjopen.bmj.com/site/about/guidelines.xhtml

Melinda Gates Foundation (PRE-EMPT initiative, grant number OPP1017337). Following input into trial design, the Gates Foundation had no role in data collection, analysis, or interpretation, or writing of the report.

AUTHOR CONTRIBUTIONS

PVD, RJD, MBB, SSG, AAM, LAM conceptualised the trial and components of the intervention. MBB, SSG, AAM, USC, GMK, UYR, AK, NVH, CCK, SGB, MV, GIM, implemented and co-ordinated monitoring of the trial. JNB and MWK performed the quantitative and qualitative analyses. AK and MWK wrote the first draft of the manuscript. All authors provided feedback and review of the manuscript.

DATA AVAILABILITY

The datasets used and/or analysed during the current study are available from the corresponding author on reasonable request.

FIGURE CAPTIONS

Figure 1. Study Location in India, Karnataka State

Figure 2. Belagavi and Bagalkote Districts, Karnataka State, India with detailed PHC areas.

Figure 3. A process evaluation plan for assessing community engagement to improve maternal health in Karnataka, India

ETHICAL APPROVAL

The project received ethics committee approvals from KLE Academy of Higher Education and Research Deemed-to-be-University (MDC/IECHSR/2011-12/A-4; ICMR 5/7/859/12-RHN) and the University of British Columbia (UBC, H12-03497).

For peer review only

REFERENCES

1. WHO, UNICEF, UNFPA, World Bank Group, The United Nations Population Division. Trends in maternal mortality 2000 to 2017. Geneva: World Health Organization; 2019.

2. Goldenberg RL, McClure EM, Saleem S. Improving pregnancy outcomes in low- and middle-income countries. Reproductive Health. 2018 Jun 22;15(Suppl 1).

3. Thaddeus S, Maine D. Too far to walk: maternal mortality in context. Social science & medicine (1982). 1994;38(8):1091–110.

4. Meh C, Sharma A, Ram U, Fadel S, Correa N, Snelgrove J, et al. Trends in maternal mortality in India over two decades in nationally representative surveys. BJOG. 2022 Mar;129(4):550–61.

5. World Health Organization. WHO recommendation on birth preparedness and complication readiness [Internet]. The WHO Reproductive Health Library. [cited 2022 Aug 24]. Available from: https://srhr.org/rhl/article/who-recommendation-on-birth-preparedness-and-complication-readiness

6. World Health Organization. WHO recommendations on health promotion interventions for maternal and newborn health [Internet]. Geneva: World Health Organization; 2015 [cited 2022 Aug 24]. Available from: https://www.who.int/publications/i/item/9789241508742

7. JHPIEGO. Monitoring Birth Preparedness and Complications Readiness: Tools and Indicators for Maternal and Newborn Health. Baltimore; 2004.

8. Cyril S, Smith BJ, Possamai-Inesedy A, Renzaho AMN. Exploring the role of community engagement in improving the health of disadvantaged populations: A systematic review. Global Health Action. 2015;8.

9. About Accredited Social Health Activist (ASHA) :: National Health Mission [Internet]. [cited 2022 Aug 24]. Available from: https://nhm.gov.in/index1.php?lang=1&level=1&sublinkid=150&lid=226

10. Vidler M, Ramadurg U, Charantimath U, Katageri G, Karadiguddi C, Sawchuck D, et al. Utilization of maternal health care services and their determinants in Karnataka State, India. Reproductive Health. 2016 Jun 8;13(1):37.

11. Lassi ZS, Kumar R, Bhutta ZA. Community-Based Care to Improve Maternal, Newborn, and Child Health. In: Disease Control Priorities, Third Edition (Volume 2): Reproductive, Maternal, Newborn, and Child Health. Washington DC: The World Bank; 2016. p. 263–84.

12. Ricca J, Kureshy N, Leban K, Prosnitz D, Ryan L. Community-based intervention packages facilitated by NGOs demonstrate plausible evidence for child mortality impact. Health Policy and Planning. 2014 Mar;29(2):204–16.

For peer review only - http://bmjopen.bmj.com/site/about/guidelines.xhtml

13. Rockers PC, Fink G, Zanolini A, Banda B, Biemba G, Sullivan C, et al. Impact of a community-based package of interventions on child development in Zambia: A cluster-randomised controlled trial. BMJ Global Health. 2016 Nov 1;1(3):104.

14. Prost A, Colbourn T, Seward N, Azad K, Coomarasamy A, Copas A, et al. Women's groups practising participatory learning and action to improve maternal and newborn health in low-resource settings: a systematic review and meta-analysis. The Lancet. 2013 May 18;381(9879):1736–46.

15. Sadruddin S, Khan I ul H, Bari A, Khan A, Ahmad I, Qazi SA. Effect of community mobilization on appropriate care seeking for pneumonia in Haripur, Pakistan. Journal of Global Health. 2015 Jun;5(1):010405.

16. Mochache V, Irungu E, El-Busaidy H, Temmerman M, Gichangi P. "Our voices matter": A before-after assessment of the effect of a community-participatory intervention to promote uptake of maternal and child health services in Kwale, Kenya 11 Medical and Health Sciences 1117 Public Health and Health Services. BMC Health Services Research. 2018 Dec 4;18(1).

17. Butterfoss FD, Francisco VT. Evaluating community partnerships and coalitions with practitioners in mind. Health promotion practice. 2004 Apr 30;5(2):108–14.

18. NHS Department of Health and Human Services. Principles of Community Engagement. Second edi. NIH Publication No. 11-7782. Bethesda, Maryland: National Institutes of Health (NIH); 2011.

19. Johnston KA. Toward a Theory of Social Engagement. In: Johnston KA, Taylor M, editors. The Handbook of Communication Engagement. Hoboken, NJ, USA: John Wiley & Sons, Inc.; 2018. p. 17–32.

20. National Institute for Health and Care Excellence (NICE). Community engagement: improving health and wellbeing and reducing health inequalities . London: NICE; 2016.

21. Balcázar HG, de Heer H, Rosenthal L, Aguirre M, Flores L, Puentes FA, et al. A Promotores de Salud intervention to reduce cardiovascular disease risk in a high-risk hispanic border population, 2005-2008. Preventing Chronic Disease. 2010;7(2).

22. Wells KB, Jones L, Chung B, Dixon EL, Tang L, Gilmore J, et al. Community-partnered cluster-randomized comparative effectiveness trial of community engagement and planning or resources for services to address depression disparities. Journal of General Internal Medicine. 2013;28(10):1268–78.

23. Bellad MB, Goudar SS, Mallapur AA, Sharma S, Bone J, Charantimath US, et al. Community level interventions for pre-eclampsia (CLIP) in India: A cluster randomised controlled trial. Pregnancy Hypertension. 2020 Jul 1;21:166–75.

24. Magee LA, Sharma S, Nathan HL, Adetoro OO, Bellad MB, Goudar S, et al. The incidence of pregnancy hypertension in India, Pakistan, Mozambique, and Nigeria: A prospective population-level analysis. PLOS Medicine. 2019;16(4):e1002783.

For peer review only

25. Government of India. Census of India 2011: Our Census, Our Future. Delhi: Office of The Registrar General & Census Commissioner;

26. International Institute for Population Sciences (IIPS). National Family Health Survey (NFHS-4), 2015-16: India. Mumbai; 2017.

27. Lim J, Cloete G, Dunsmuir DT, Payne BA, Scheffer C, von Dadelszen P, et al. Usability and Feasibility of PIERS on the Move: An mHealth App for Pre-Eclampsia Triage. JMIR mHealth and uHealth. 2015 Apr 17;3(2):e37.

28. Payne B, Dunsmuir D, Hall D, Lim J, Sikandar R, Magee L, et al. Designing an evidence-based mobile health decision aid for the management of hypertensive disorders of pregnancy (HDP) in low-resourced settings. BMC Health Services Research. 2014 Jul 7;14(S2):P93.

29. Goudar SS, Carlo WA, McClure EM, Pasha O, Patel A, Esamai F, et al. The maternal and newborn health registry study of the global network for women's and children's health research. International Journal of Gynecology and Obstetrics. 2012;118(3):190–3.

30. Fetters MD, Curry LA, Creswell JW. Achieving Integration in Mixed Methods Designs—Principles and Practices. Health Services Research. 2013 Dec 1;48(6pt2):2134–56.

31. Sharma S, Adetoro OO, Vidler M, Drebit S, Payne BA, Akeju DO, et al. A process evaluation plan for assessing a complex community-based maternal health intervention in Ogun State, Nigeria. BMC Health Services Research. 2017 Mar 28;17(1).

32. Yen TW, Payne B, Qu Z, Hutcheon JA, Lee T, Magee LA, et al. Using clinical symptoms to predict adverse maternal and perinatal outcomes in women with preeclampsia: data from the PIERS (Pre-eclampsia Integrated Estimate of RiSk) study. J Obstet Gynaecol Can. 2011 Aug;33(8):803–9.

33. Braun V, Clarke V. Using thematic analysis in psychology. Qualitative Research in Psychology. 2006;3(2):77–101.

34. Bellad MB, Goudar SS, Mallapur AA, Sharma S, Bone J, Charantimath US, et al. Community level interventions for pre-eclampsia (CLIP) in India: A cluster randomised controlled trial. Pregnancy Hypertension. 2020 Jul 1;21:166–75.

35. Marston C, Renedo A, McGowan CR, Portela A. Effects of Community Participation on Improving Uptake of Skilled Care for Maternal and Newborn Health: A Systematic Review. Braitstein P, editor. PLoS ONE. 2013 Feb 4;8(2):e55012.

36. Kumar V, Kumar A, Das V, Srivastava NM, Baqui AH, Santosham M, et al. Community-driven impact of a newborn-focused behavioral intervention on maternal health in Shivgarh, India. International Journal of Gynecology and Obstetrics. 2012;117(1):48–55.

For peer review only

For peer review only - http://bmjopen.bmj.com/site/about/guidelines.xhtml

37. Dandona L, Dandona R, Kumar GA, Shukla DK, Paul VK, Balakrishnan K, et al. Nations within a nation: variations in epidemiological transition across the states of India, 1990–2016 in the Global Burden of Disease Study. The Lancet. 2017 Dec 2;390(10111):2437–60.

38. MacQueen KM, Bhan A, Frohlich J, Holzer J, Sugarman J. Evaluating community engagement in global health research: The need for metrics Ethics in Biomedical Research. BMC Medical Ethics. 2015 Jul 1;16(1):44.

39. Solnes Miltenburg A, Roggeveen Y, Shields L, van Elteren M, van Roosmalen J, Stekelenburg J, et al. Impact of Birth Preparedness and Complication Readiness Interventions on Birth with a Skilled Attendant: A Systematic Review. Niederkrotenthaler T, editor. PLOS ONE. 2015 Nov 23;10(11):e0143382.

40. Vidler M, Charantimath U, Katageri G, Ramadurg U, Karadiguddi C, Sawchuck D, et al. Community perceptions of pre-eclampsia in rural Karnataka State, India: A qualitative study. Reproductive Health. 2016 Jun 8;13(1):35.

For peer review only

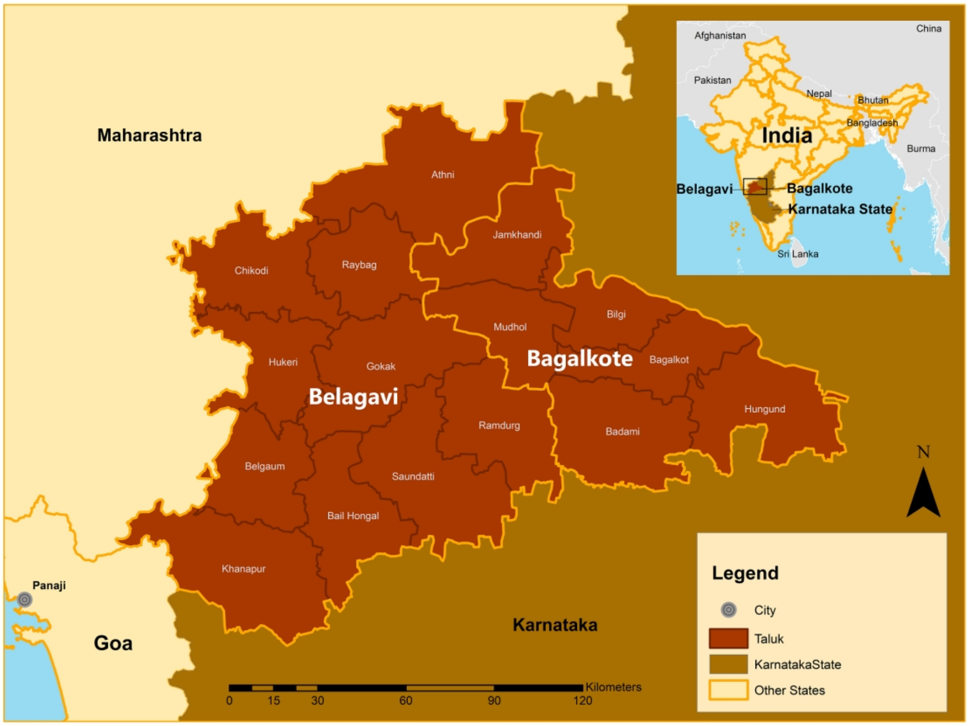

Study Location in India, Karnataka State

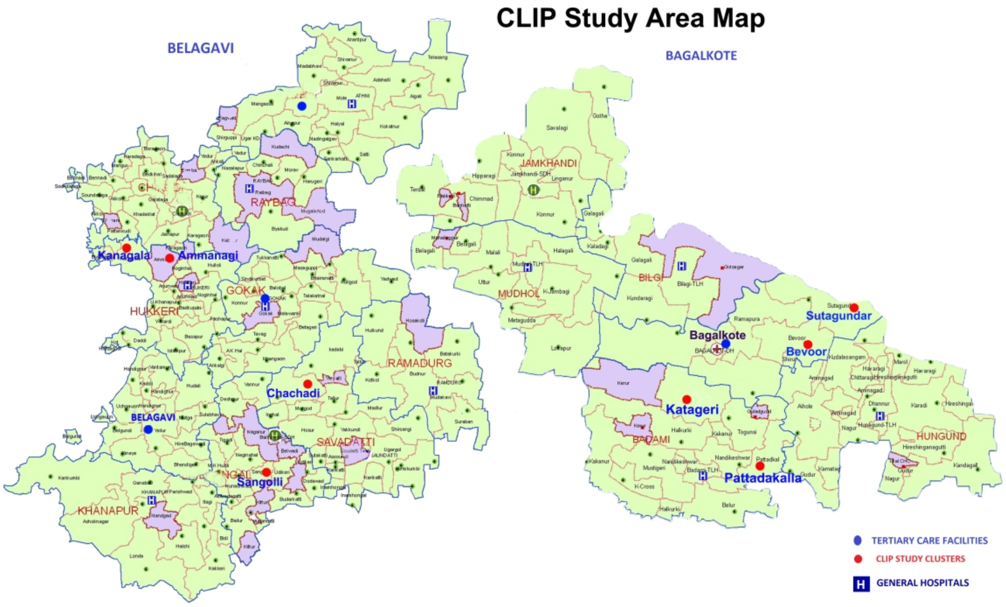

Belagavi and Bagalkote Districts, Karnataka State, India with detailed PHC areas.

793x474mm (144 x 144 DPI)

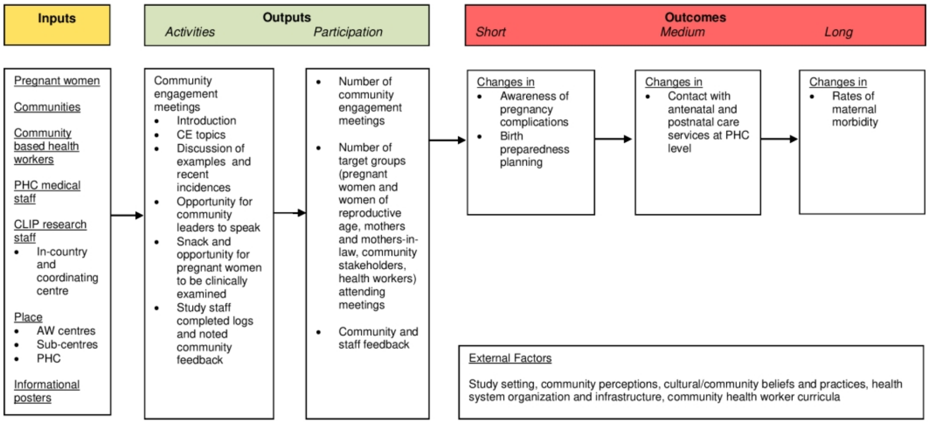

A process evaluation plan for assessing community engagement to improve maternal health in Karnataka, India

279x215mm (200 x 200 DPI)

SUPPLEMENTARY FILES

**Table S1:** Consort 2020 Checklist	Page 2
**Table S2:** Standards for Reporting Qualitative Research (SRQR) Checklist	Page 6
**Table S3:** Summary of pre-eclampsia knowledge by cluster [n (%)]	Page 9
**Table S4:** Summary of birth preparedness by cluster [n (%)]	Page 11
**Figure S1:** Informational poster on danger signs of hypertensive disorders of pregnancy	Page 12

For peer review only

Table S1 Consort 2020 Checklist

CONSORT 2010 checklist of information to include when reporting a randomised trial*

Section/Topic	Item No	Checklist item	Reported on page No
**Title and abstract**			
	1a	Identification as a randomised trial in the title	Page 1
	1b	Structured summary of trial design, methods, results, and conclusions (for specific guidance see CONSORT for abstracts)	Page 2
**Introduction**			
Background and objectives	2a	Scientific background and explanation of rationale	Page 4-5
	2b	Specific objectives or hypotheses	Page 5
**Methods**			
Trial design	3a	Description of trial design (such as parallel, factorial) including allocation ratio	Page 5
	3b	Important changes to methods after trial commencement (such as eligibility criteria), with reasons	N/A*
Participants	4a	Eligibility criteria for participants	Page 5-6
	4b	Settings and locations where the data were collected	Page 5
Interventions	5	The interventions for each group with sufficient details to allow replication, including how and when they were actually administered	Page 6

For peer review only - http://bmjopen.bmj.com/site/about/guidelines.xhtml

Outcomes	6a	Completely defined pre-specified primary and secondary outcome measures, including how and when they were assessed	Page 7
	6b	Any changes to trial outcomes after the trial commenced, with reasons	N/A*
Sample size	7a	How sample size was determined	N/A*
	7b	When applicable, explanation of any interim analyses and stopping guidelines	N/A*
Randomisation:			
Sequence generation	8a	Method used to generate the random allocation sequence	Page 5
	8b	Type of randomisation; details of any restriction (such as blocking and block size)	Page 5
Allocation concealment mechanism	9	Mechanism used to implement the random allocation sequence (such as sequentially numbered containers), describing any steps taken to conceal the sequence until interventions were assigned	Page 5
Implementation	10	Who generated the random allocation sequence, who enrolled participants, and who assigned participants to interventions	Page 5
Blinding	11a	If done, who was blinded after assignment to interventions (for example, participants, care providers, those assessing outcomes) and how	N/A*
	11b	If relevant, description of the similarity of interventions	N/A*
Statistical methods	12a	Statistical methods used to compare groups for primary and secondary outcomes	Page 7-8
	12b	Methods for additional analyses, such as subgroup analyses and adjusted analyses	
**Results**			
Participant flow (a diagram is	13a	For each group, the numbers of participants who were randomly assigned, received intended treatment, and were analysed for the primary outcome	Page 5*

For peer review only

strongly recommended)	13b	For each group, losses and exclusions after randomisation, together with reasons	N/A*
Recruitment	14a	Dates defining the periods of recruitment and follow-up	Page 6
	14b	Why the trial ended or was stopped	Page 6
Baseline data	15	A table showing baseline demographic and clinical characteristics for each group	Page 5*
Numbers analysed	16	For each group, number of participants (denominator) included in each analysis and whether the analysis was by original assigned groups	N/A*
Outcomes and estimation	17a	For each primary and secondary outcome, results for each group, and the estimated effect size and its precision (such as 95% confidence interval)	Page 9-11
	17b	For binary outcomes, presentation of both absolute and relative effect sizes is recommended	Page 5*
Ancillary analyses	18	Results of any other analyses performed, including subgroup analyses and adjusted analyses, distinguishing pre-specified from exploratory	N/A*
Harms	19	All important harms or unintended effects in each group (for specific guidance see CONSORT for harms)	N/A*
**Discussion**			
Limitations	20	Trial limitations, addressing sources of potential bias, imprecision, and, if relevant, multiplicity of analyses	Page 14
Generalisability	21	Generalisability (external validity, applicability) of the trial findings	Page 13-14
Interpretation	22	Interpretation consistent with results, balancing benefits and harms, and considering other relevant evidence	Page 13-14

For peer review only - http://bmjopen.bmj.com/site/about/guidelines.xhtml

**Other information**			
Registration	23	Registration number and name of trial registry	Page 5
Protocol	24	Where the full trial protocol can be accessed, if available	N/A*
Funding	25	Sources of funding and other support (such as supply of drugs), role of funders	Page 15

*For more details on the methodology, implementation and results of the full CLIP Trial in India, please see: Bellad MB, Goudar SS, Mallapur AA, Sharma S, Bone J, Charantimath US, et al. Community level interventions for pre-eclampsia (CLIP) in India: A cluster randomised controlled trial. Pregnancy Hypertension. 2020 Jul 1;21:166–75.

For peer review only

Table S2: Standards for Reporting Qualitative Research (SRQR) Checklist

Title and abstract

	**Page/line no(s).**
**Title** - Concise description of the nature and topic of the study Identifying the study as qualitative or indicating the approach (e.g., ethnography, grounded theory) or data collection methods (e.g., interview, focus group) is recommended	Page 1
**Abstract** - Summary of key elements of the study using the abstract format of the intended publication; typically includes background, purpose, methods, results, and conclusions	Page 2

Introduction

**Problem formulation** - Description and significance of the problem/phenomenon studied; review of relevant theory and empirical work; problem statement	Page 4-5
**Purpose or research question** - Purpose of the study and specific objectives or questions	Page 5

Methods

**Qualitative approach and research paradigm** - Qualitative approach (e.g., ethnography, grounded theory, case study, phenomenology, narrative research) and guiding theory if appropriate; identifying the research paradigm (e.g., postpositivist, constructivist/ interpretivist) is also recommended; rationale**	Page 8
**Researcher characteristics and reflexivity** - Researchers' characteristics that may influence the research, including personal attributes, qualifications/experience, relationship with participants, assumptions, and/or presuppositions; potential or actual interaction between researchers' characteristics and the research questions, approach, methods, results, and/or transferability	Page 8

For peer review only - http://bmjopen.bmj.com/site/about/guidelines.xhtml

**Context** - Setting/site and salient contextual factors; rationale**	Page 5-6
**Sampling strategy** - How and why research participants, documents, or events were selected; criteria for deciding when no further sampling was necessary (e.g., sampling saturation); rationale**	Page 6-7
**Ethical issues pertaining to human subjects** - Documentation of approval by an appropriate ethics review board and participant consent, or explanation for lack thereof; other confidentiality and data security issues	Page 8
**Data collection methods** - Types of data collected; details of data collection procedures including (as appropriate) start and stop dates of data collection and analysis, iterative process, triangulation of sources/methods, and modification of procedures in response to evolving study findings; rationale**	Page 8
**Data collection instruments and technologies** - Description of instruments (e.g., interview guides, questionnaires) and devices (e.g., audio recorders) used for data collection; if/how the instrument(s) changed over the course of the study	Page 8
**Units of study** - Number and relevant characteristics of participants, documents, or events included in the study; level of participation (could be reported in results)	Page 6
**Data processing** - Methods for processing data prior to and during analysis, including transcription, data entry, data management and security, verification of data integrity, data coding, and anonymization/de-identification of excerpts	Page 8
**Data analysis** - Process by which inferences, themes, etc., were identified and developed, including the researchers involved in data analysis; usually references a specific paradigm or approach; rationale**	Page 8
**Techniques to enhance trustworthiness** - Techniques to enhance trustworthiness and credibility of data analysis (e.g., member checking, audit trail, triangulation); rationale**	Page 8

Results/findings

For peer review only - http://bmjopen.bmj.com/site/about/guidelines.xhtml

**Synthesis and interpretation** - Main findings (e.g., interpretations, inferences, and themes); might include development of a theory or model, or integration with prior research or theory	Page 11-13
**Links to empirical data** - Evidence (e.g., quotes, field notes, text excerpts, photographs) to substantiate analytic findings	Page 11-13

Discussion

**Integration with prior work, implications, transferability, and contribution(s) to the field -** Short summary of main findings; explanation of how findings and conclusions connect to, support, elaborate on, or challenge conclusions of earlier scholarship; discussion of scope of application/generalizability; identification of unique contribution(s) to scholarship in a discipline or field	Page 13-14
**Limitations** - Trustworthiness and limitations of findings	Page 14

Other

**Conflicts of interest** - Potential sources of influence or perceived influence on study conduct and conclusions; how these were managed	Page 15
**Funding** - Sources of funding and other support; role of funders in data collection, interpretation, and reporting	Page 15

For peer review only - http://bmjopen.bmj.com/site/about/guidelines.xhtml

Table S3:Summary of pre-eclampsia knowledge by cluster [n (%)]

	Intervention Clusters						Control Clusters						Total
	**Hosur**	**Kanagala**	**Sangolli**	**Pattadaka**	**Amingad**	**Sutugundar**	**Aihole**	**Galagali**	**Katageri**	**Belavadi**	**Chachadi**	**Ammangi**	
Composite	122 (7.5)	17 (1.6)	62 (5.9)	4 (0.3)	221 (13.2)	13 (1.3)	6 (0.6)	94 (6.9)	12 (1.1)	3 (0.2)	26 (2.0)	10 (1.0)	590 (3.4)
Can name at least 4 symptoms	122 (7.0)	17 (1.6)	62 (5.9)	4 (0.3)	223 (13.3)	13 (1.3)	6 (0.6)	96 (7.0)	12 (1.1)	3 (0.2)	26 (2.0)	10 (1.0)	594 (4.0)
Aware women can have abnormal bleeding in pregnancy	656 (40.3)	325 (30.5)	406 (38.8)	296 (21.3)	171 (10.2)	127 (12.3)	36 (3.9)	1034 (75.4)	268 (24.1)	494 (39.0)	442 (34.2)	94 (9.5)	4349 (29.4)
Aware women can have seizure in pregnancy	624 (38.3)	316 (29.7)	345 (32.9)	276 (19.8)	145 (8.6)	108 (10.5)	19 (2.1)	810 (59.1)	260 (23.4)	334 (26.4)	236 (18.3)	32 (3.2)	3505 (23.7)
Aware women can have high blood pressure in pregnancy	817 (50.1)	584 (54.8)	553 (52.8)	264 (19.0)	732 (43.6)	193 (18.8)	38 (4.1)	1000 (72.9)	339 (30.5)	718 (56.7)	512 (39.7)	466 (47.2)	6216 (42.1)

For peer review only

Aware high blood pressure in pregnancy can be life threatening	793 (48.7)	584 (54.8)	552 (52.7)	232 (16.7)	736 (43.8)	189 (18.4)	37 (4.0)	968 (70.6)	232 (20.9)	626 (49.5)	511 (39.6)	466 (47.2)	5926 (40.1)

For peer review only

Table S4: Summary of birth preparedness by cluster [n (%)]

	Intervention Clusters						Control Clusters						Total
	Hosur	Kanagala	Sangolli	Pattadakal lu	Amingad	Sutugunda r	Aihole	Galagali	Katageri	Belavadi	Chachadi	Ammangi	
Composite	1556 (95.5)	1013 (95.1)	1044 (99.7)	869 (62.5)	466 (27.8)	639 (62.1)	702 (76.5)	878 (64.0)	827 (74.4)	1241 (98.0)	1289 (99.9)	932 (94.4)	11456 (77.5)
Arranged transport	1548 (95.0)	995 (93.4)	1044 (99.7)	1160 (83.5)	475 (28.3)	887 (86.2%)	720 (78.4)	1065 (77.7)	859 (77.3)	1262 (99.7)	1291 (100.0)	848 (85.9)	12154 (82.2)
Has permission for emergency care	1626 (99.8)	1063 (99.8)	1045 (99.8)	1372 (98.7)	1634 (97.3)	855 (83.0)	909 (99.0)	1362 (99.3)	1015 (91.4)	1264 (99.8)	1291 (100.0)	984 (99.7)	14420 (97.5)
Has money saved for emergency	1324 (81.2)	1008 (94.6)	907 (86.6)	127 (9.1)	385 (22.9)	118 (11.4)	481 (52.4)	327 (23.9)	364 (32.8)	723 (57.1)	1168 (90.5)	977 (99.0)	7909 (53.5)
Identified facility for delivery	1572 (96.5)	1058 (99.3)	1002 (95.7)	1011 (72.7)	1377 (82.0)	836 (81.2)	745 (81.2)	997 (72.7)	874 (78.7)	1210 (95.6)	1266 (98.1)	903 (91.5)	12851 (86.9)

For peer review only - http://bmjopen.bmj.com/site/about/guidelines.xhtml

Figure S1: Informational poster on danger signs of hypertensive disorders of pregnancy

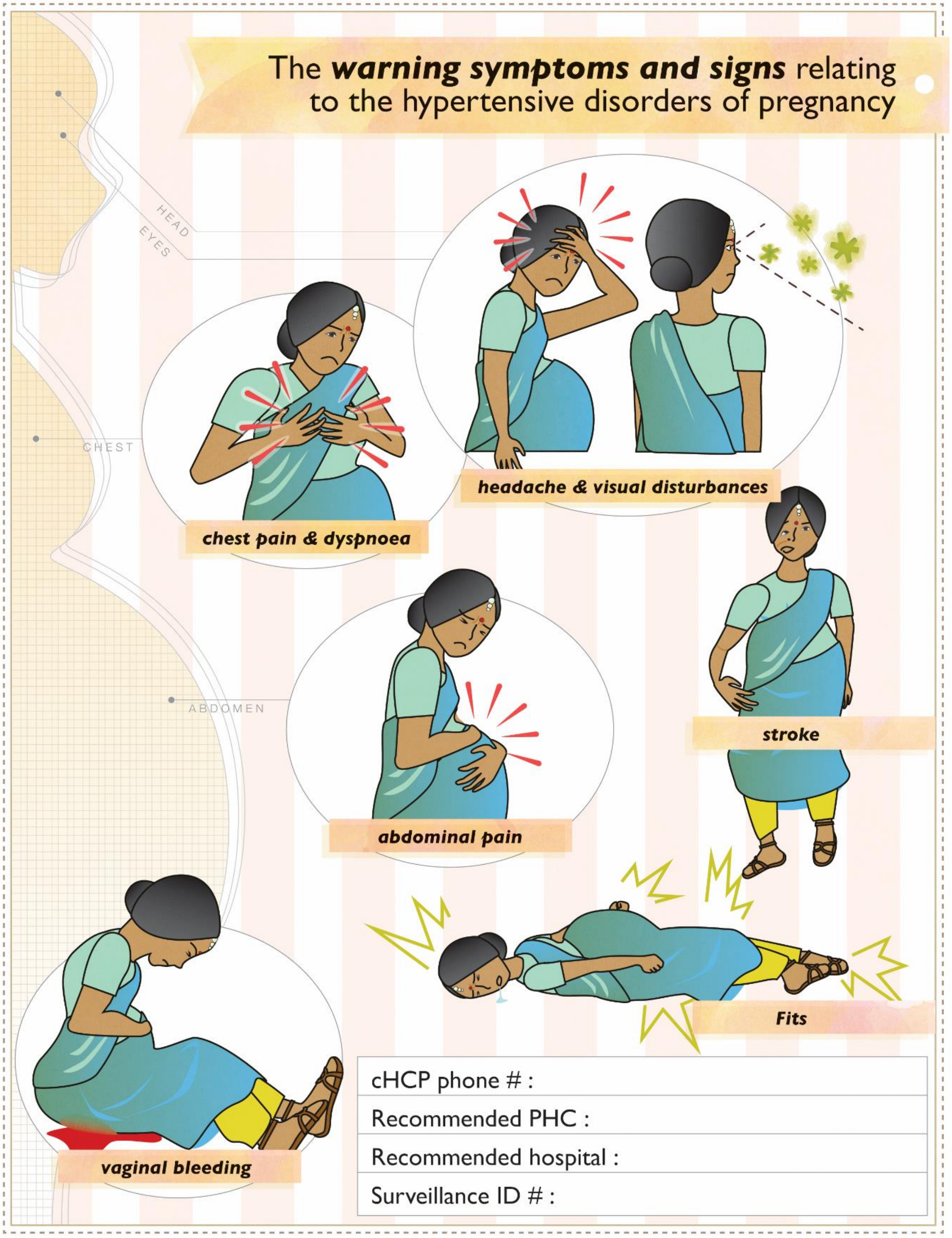

CONSORT 2010 checklist of information to include when reporting a randomised trial*

Section/Topic	Item No	Checklist item	Reported on page No
**Title and abstract**			
	1a	Identification as a randomised trial in the title	Page 1
	1b	Structured summary of trial design, methods, results, and conclusions (for specific guidance see CONSORT for abstracts)	Page 2
**Introduction**			
Background and objectives	2a	Scientific background and explanation of rationale	Page 4
	2b	Specific objectives or hypotheses	Page 4
**Methods**			
Trial design	3a	Description of trial design (such as parallel, factorial) including allocation ratio	Page 4-5
	3b	Important changes to methods after trial commencement (such as eligibility criteria), with reasons	N/A*
Participants	4a	Eligibility criteria for participants	Page 5
	4b	Settings and locations where the data were collected	Page 5
Interventions	5	The interventions for each group with sufficient details to allow replication, including how and when they were actually administered	Page 5-6
Outcomes	6a	Completely defined pre-specified primary and secondary outcome measures, including how and when they were assessed	Page 7
	6b	Any changes to trial outcomes after the trial commenced, with reasons	N/A*

For peer review only

Sample size	7a	How sample size was determined	N/A*
	7b	When applicable, explanation of any interim analyses and stopping guidelines	N/A*
Randomisation:			
Sequence generation	8a	Method used to generate the random allocation sequence	Page 5
	8b	Type of randomisation; details of any restriction (such as blocking and block size)	Page 5
Allocation concealment mechanism	9	Mechanism used to implement the random allocation sequence (such as sequentially numbered containers), describing any steps taken to conceal the sequence until interventions were assigned	N/A*
Implementation	10	Who generated the random allocation sequence, who enrolled participants, and who assigned participants to interventions	N/A*
Blinding	11a	If done, who was blinded after assignment to interventions (for example, participants, care providers, those assessing outcomes) and how	N/A*
	11b	If relevant, description of the similarity of interventions	N/A*
Statistical methods	12a	Statistical methods used to compare groups for primary and secondary outcomes	Page 6
	12b	Methods for additional analyses, such as subgroup analyses and adjusted analyses	Page 6
**Results**			
Participant flow (a diagram is strongly recommended)	13a	For each group, the numbers of participants who were randomly assigned, received intended treatment, and were analysed for the primary outcome	N/A*
	13b	For each group, losses and exclusions after randomisation, together with reasons	N/A*
Recruitment	14a	Dates defining the periods of recruitment and follow-up	Page 5

For peer review only

	14b	Why the trial ended or was stopped	N/A*
Baseline data	15	A table showing baseline demographic and clinical characteristics for each group	Page 7-8, table 1
Numbers analysed	16	For each group, number of participants (denominator) included in each analysis and whether the analysis was by original assigned groups	Page 8-9, tables 2 and 3
Outcomes and estimation	17a	For each primary and secondary outcome, results for each group, and the estimated effect size and its precision (such as 95% confidence interval)	Page 8-9, tables 2 and 3
	17b	For binary outcomes, presentation of both absolute and relative effect sizes is recommended	N/A*
Ancillary analyses	18	Results of any other analyses performed, including subgroup analyses and adjusted analyses, distinguishing pre-specified from exploratory	N/A*
Harms	19	All important harms or unintended effects in each group (for specific guidance see CONSORT for harms)	N/A*
**Discussion**			
Limitations	20	Trial limitations, addressing sources of potential bias, imprecision, and, if relevant, multiplicity of analyses	Page 13
Generalisability	21	Generalisability (external validity, applicability) of the trial findings	Page 13
Interpretation	22	Interpretation consistent with results, balancing benefits and harms, and considering other relevant evidence	Page 12-13
**Other information**			
Registration	23	Registration number and name of trial registry	Page 4
Protocol	24	Where the full trial protocol can be accessed, if available	N/A*

For peer review only

Funding	25	Sources of funding and other support (such as supply of drugs), role of funders	Page 14

*For more details on the methodology, implementation and results of the full CLIP Trial in India, please see: Bellad MB, Goudar SS, Mallapur AA, Sharma S, Bone J, Charantimath US, Katageri GM, Ramadurg UY, Mark Ansermino J, Derman RJ, Dunsmuir DT, Honnungar NV, Karadiguddi C, Kavi AJ, Kodkany BS, Lee T, Li J, Nathan HL, Payne BA, Revankar AP, Shennan AH, Singer J, Tu DK, Vidler M, Wong H, Bhutta ZA, Magee LA, von Dadelszen P; CLIP India Working Group. Community level interventions for pre-eclampsia (CLIP) in India: A cluster randomised controlled trial. Pregnancy Hypertens. 2020 Jul;21:166-175. doi: 10.1016/j.preghy.2020.05.008. Epub 2020 May 19. PMID: 32554291; PMCID: PMC7471838.

For peer review only

Standards for Reporting Qualitative Research (SRQR)*

http://www.equator-network.org/reporting-guidelines/srqr/

Title and abstract

	Page/line no(s).
**Title** - Concise description of the nature and topic of the study Identifying the study as qualitative or indicating the approach (e.g., ethnography, grounded theory) or data collection methods (e.g., interview, focus group) is recommended	Page 1
**Abstract** - Summary of key elements of the study using the abstract format of the intended publication; typically includes background, purpose, methods, results, and conclusions	Page 2

Introduction

**Problem formulation** - Description and significance of the problem/phenomenon studied; review of relevant theory and empirical work; problem statement	Page 4-5
**Purpose or research question** - Purpose of the study and specific objectives or questions	Page 5

Methods

**Qualitative approach and research paradigm** - Qualitative approach (e.g., ethnography, grounded theory, case study, phenomenology, narrative research) and guiding theory if appropriate; identifying the research paradigm (e.g., postpositivist, constructivist/ interpretivist) is also recommended; rationale**	Page 8
**Researcher characteristics and reflexivity** - Researchers' characteristics that may influence the research, including personal attributes, qualifications/experience, relationship with participants, assumptions, and/or presuppositions; potential or actual interaction between researchers' characteristics and the research questions, approach, methods, results, and/or transferability	Page 8
**Context** - Setting/site and salient contextual factors; rationale**	Page 5-6
**Sampling strategy** - How and why research participants, documents, or events were selected; criteria for deciding when no further sampling was necessary (e.g., sampling saturation); rationale**	Page 6-7
**Ethical issues pertaining to human subjects** - Documentation of approval by an appropriate ethics review board and participant consent, or explanation for lack thereof; other confidentiality and data security issues	Page 8
**Data collection methods** - Types of data collected; details of data collection procedures including (as appropriate) start and stop dates of data collection and analysis, iterative process, triangulation of sources/methods, and modification of procedures in response to evolving study findings; rationale**	Page 8

For peer review only

For peer review only - http://bmjopen.bmj.com/site/about/guidelines.xhtml

**Data collection instruments and technologies** - Description of instruments (e.g., interview guides, questionnaires) and devices (e.g., audio recorders) used for data collection; if/how the instrument(s) changed over the course of the study	Page 8
**Units of study** - Number and relevant characteristics of participants, documents, or events included in the study; level of participation (could be reported in results)	Page 6
**Data processing** - Methods for processing data prior to and during analysis, including transcription, data entry, data management and security, verification of data integrity, data coding, and anonymization/de-identification of excerpts	Page 8
**Data analysis** - Process by which inferences, themes, etc., were identified and developed, including the researchers involved in data analysis; usually references a specific paradigm or approach; rationale**	Page 8
**Techniques to enhance trustworthiness** - Techniques to enhance trustworthiness and credibility of data analysis (e.g., member checking, audit trail, triangulation); rationale**	Page 8

Results/findings

**Synthesis and interpretation** - Main findings (e.g., interpretations, inferences, and themes); might include development of a theory or model, or integration with prior research or theory	Page 11-13
**Links to empirical data** - Evidence (e.g., quotes, field notes, text excerpts, photographs) to substantiate analytic findings	Page 11-13

Discussion

**Integration with prior work, implications, transferability, and contribution(s) to the field** - Short summary of main findings; explanation of how findings and conclusions connect to, support, elaborate on, or challenge conclusions of earlier scholarship; discussion of scope of application/generalizability; identification of unique contribution(s) to scholarship in a discipline or field	Page 13-14
**Limitations** - Trustworthiness and limitations of findings	Page 14

Other

**Conflicts of interest** - Potential sources of influence or perceived influence on study conduct and conclusions; how these were managed	Page 15
**Funding** - Sources of funding and other support; role of funders in data collection, interpretation, and reporting	Page 15

*The authors created the SRQR by searching the literature to identify guidelines, reporting standards, and critical appraisal criteria for qualitative research; reviewing the reference lists of retrieved sources; and contacting experts to gain feedback. The SRQR aims to improve the transparency of all aspects of qualitative research by providing clear standards for reporting qualitative research.

For peer review only

For peer review only - http://bmjopen.bmj.com/site/about/guidelines.xhtml

**The rationale should briefly discuss the justification for choosing that theory, approach, method, or technique rather than other options available, the assumptions and limitations implicit in those choices, and how those choices influence study conclusions and transferability. As appropriate, the rationale for several items might be discussed together.

Reference:

O'Brien BC, Harris IB, Beckman TJ, Reed DA, Cook DA. **Standards for reporting qualitative research: a synthesis of recommendations.** *Academic Medicine*, Vol. 89, No. 9 / Sept 2014 DOI: 10.1097/ACM.0000000000000388

For peer review only

For peer review only - http://bmjopen.bmj.com/site/about/guidelines.xhtml
